# A Record Chromophore Density in High‐Entropy Liquids of Two Low‐Melting Perylenes: A New Strategy for Liquid Chromophores

**DOI:** 10.1002/advs.201801650

**Published:** 2019-01-15

**Authors:** Khushbu Kushwaha, Liyang Yu, Kati Stranius, Sandeep Kumar Singh, Sandra Hultmark, Muhammad Naeem Iqbal, Lars Eriksson, Eric Johnston, Paul Erhart, Christian Müller, Karl Börjesson

**Affiliations:** ^1^ Department of Chemistry and Molecular Biology University of Gothenburg Kemigården 4 41296 Gothenburg Sweden; ^2^ Department of Chemistry and Chemical Engineering Chalmers University of Technology 41296 Gothenburg Sweden; ^3^ Department of Physics Materials and Surface Theory Division Chalmers University of Technology 41296 Gothenburg Sweden; ^4^ Department of Materials and Environmental Chemistry Stockholm University Stockholm Sweden; ^5^ Sigrid Therapeutics AB Sankt Göransgatan 159 112 17 Stockholm Sweden

**Keywords:** alkylation, high quantum yield, liquid fluorophores, low melting solids, thermodynamic mixing

## Abstract

Liquid chromophores constitute a rare but intriguing class of molecules that are in high demand for the design of luminescent inks, liquid semiconductors, and solar energy storage materials. The most common way to achieve liquid chromophores involves the introduction of long alkyl chains, which, however, significantly reduces the chromophore density. Here, strategy is presented that allows for the preparation of liquid chromophores with a minimal increase in molecular weight, using the important class of perylenes as an example. Two synergistic effects are harnessed: (1) the judicious positioning of short alkyl substituents, and (2) equimolar mixing, which in unison results in a liquid material. A series of 1‐alkyl perylene derivatives is synthesized and it is found that short ethyl or butyl chains reduce the melting temperature from 278 °C to as little as 70 °C. Then, two low‐melting derivatives are mixed, which results in materials that do not crystallize due to the increased configurational entropy of the system. As a result, liquid chromophores with the lowest reported molecular weight increase compared to the neat chromophore are obtained. The mixing strategy is readily applicable to other π‐conjugated systems and, hence, promises to yield a wide range of low molecular weight liquid chromophores.

## 1 Introduction

Liquid chromophores constitute an intriguing class of organic materials, often comprising an extended π‐conjugated core, while still being liquid at room temperature.^1^ This class of materials is of interest in solvent‐free applications where material flexibility, extreme concentrations, and/or intermolecular coupling are essential. Liquid chromophores are needed for luminescent inks,^2^ organic light emitting diodes,^3^ liquid semiconductors,^4^ lasers,^5^ solar energy storage,^6^ and photon upconversion.^7^ Additionally, liquid chromophores enable high‐performance strong exciton–photon coupling, where the number of chromophores per volume is of utmost importance.^8^

Most organic chromophores contain large π‐conjugated networks, resulting in strong π interactions between molecules, which raise the melting point and are, thus, the main reason why most chromophores of interest remain solid at room temperature. A number of strategies to lower the melting point below the room temperature have been explored.^1^, ^9^ In a recent report, a series of alkoxyphenyl‐substituted pyrenes with various branched alkyl chains was synthesized.^10^ This study suggested that increasing the number of phenyl rings and bulky alkyl chains, weakened the intermolecular π interactions, resulting in a liquid material at room temperature. Another example, was the introduction of branched and hyperbranched aliphatic chains in oligo(*p*‐phenylene vinylenes) leading to highly dense but monomerically dispersed π‐cores as an emissive liquid.^11^ However, the large volume of the side groups significantly reduced the volume concentration of the chromophores. This is true for about every conjugated organic molecule synthesized today, since side chains dilute the optoelectronically active conjugated core (i.e., lower the absorption per weight or volume). Hence, it is necessary to develop liquid chromophores with a minimum of liquidizing side chains to maximize the volume concentration and to allow for orbital overlap between chromophores.

Perylene and its derivatives constitute an important class of chromophores due to their thermal and photochemical stability, high molar absorptivity, and near unity fluorescence quantum yield (94% in cyclohexane).^12^ Perylenediimides (PDI) form the most common derivatives of perylene^13^ and much effort has been dedicated to their modification, for instance by substitutions at the imide positions with long alkyl chains to increase solubility.^14^ Interestingly, it is found that substituting PDIs at any of the four bay positions results in twisting of the perylene core due to steric hindrance. The resulting deviation from a planar conformation reduces the π‐stacking interaction and leads to increased solubility. Moreover, the twisting of the core induces axial chirality resulting in (P) and (M) atropoenantiomers, which may also reduce the tendency to crystallize.^15^ Hence, there is a growing interest in such contorted perylenes.^16^ Although a significant number of studies on twisted PDIs exist, less attention has been paid to alkylated perylenes. In the 1960s, some 1‐*n*‐alkylperylenes were reported in low yields by reacting perylene with alkyllithiums, but their properties were not studied.^17^ Recently, Zeng and coworkers reported the synthesis of ortho‐tetrabromo‐bay‐tetrabutoxyperylene as a building block for obtaining octa‐substituted perylenes.^18^ Moreover, Müllen and coworkers described the synthesis of peri‐tetrabromo‐bay‐tetrachloroperylene, which can be modified further to obtain other functionalized perylenes.^19^

To investigate if small side chains introduced at the bay position would reduce the melting point of perylene, we synthesized ten perylene derivatives with a variety of aliphatic substituents. A reduction of the melting point was found to various extents in most constitutional isomers. Here, we demonstrate that solvent‐free liquids with a record chromophore density can be realized by introducing two concepts: (1) low molar mass aliphatic substituents and (2) high‐entropy mixtures of two chromophores.^20^ The resulting materials demonstrate by far the smallest substituents reported for a liquid chromophore, with a mere 17% increase of the molar mass compared to the unsubstituted chromophore, thus, maximizing chromophore concentration and allowing for significant intermolecular orbital overlap. It is further postulated that the method employed here, i.e., thermodynamic mixing of chromophores having small alkyl substituents, constitutes a general method for developing low‐molecular weight liquid chromophores.

## 2 Results and Discussions

### 2.1 Synthesis of Alkyl‐Substituted Perylenes

**Scheme 1:**
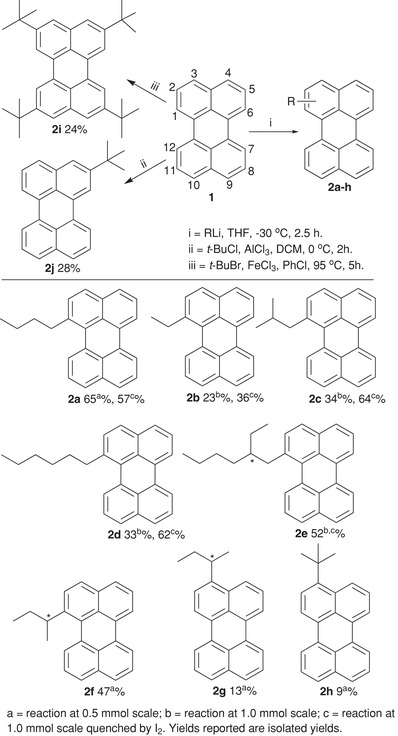
Synthesis of alkylated perylenes.

**Figure 1:**
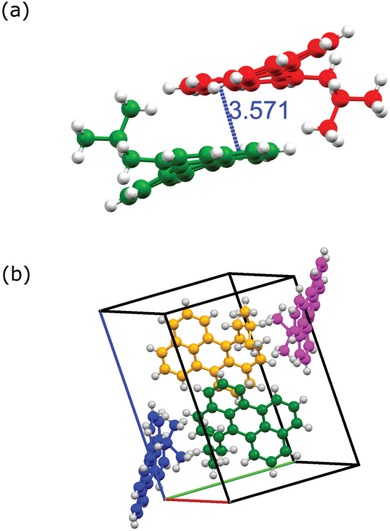
a) Structure of two enantiomers of 1‐*iso*‐butylperylene (**2c**) shown at a shortest distance (Å). b) Packing of 1‐*iso*‐butylperylene molecules in a unit cell, image viewed along 111 from refined data.

The synthesis of alkylated π‐chromophores typically requires multistep reaction sequences, which is time consuming. Therefore, we focused on one‐step reactions to obtain ten perylene derivatives having alkyl substituents at positions 1, 2, or 3 (**Scheme** **1**). There are comparably few reports on functionalization of perylene (as compared to PDI), and they mostly refer to electrophilic substitution at the 3‐position.[[qv: 17b,21]] The first cases of substitution of perylene at a position other than 3 were reported by Zieger in 1964[[qv: 17a]] and 1966,[[qv: 17b]] who described the synthesis of 1‐*n*‐butylperylene in 13% and 43% yields, respectively, by reacting perylene with *n*‐BuLi in tetrahydrofuran (THF). The position of alkylation was assigned by comparing the Infrared, ^1^H nuclear magnetic resonance (NMR), and ultraviolet spectra of the synthesized compound with known 3‐hexylperylene and 2‐ethylperylene.[[qv: 17a]] Here, we report the first single crystal X‐ray of a 1‐alkylperylene derivative (**2c**), thus, unambiguously confirming the proposed structure and demonstrating that alkylation is taking place at the 1‐position with primary alkyllithiums (**Figure** **1**a, b, see Supporting Information for coordinates). We chose a synthetic route inspired by the original procedure by Zieger et al., i.e., perylene was allowed to react with *n*‐BuLi giving the desired 1‐*n*‐butylperylene (**2a**) in 65% yield (Scheme 1). With *s*‐BuLi, both the 1‐ and 3‐isomeric *sec*‐butylperylenes were obtained, whereas reaction with *t*‐BuLi afforded only 3‐*tert*‐butylperylene (**2h**) in 9% yield. To acquire a larger amount of material, the reaction was scaled up (initial scale was 0.5 mmol). This, however, leads to a considerable reduction of the yields for synthesizing 1‐ethylperylene (**2b**), 1‐*iso*‐butylperylene (**2c**), and 1‐*n*‐hexylperylene (**2d**). In these cases, an unknown side‐product or intermediate present in 10–20% existed in the reaction mixture.

**Scheme 2:**
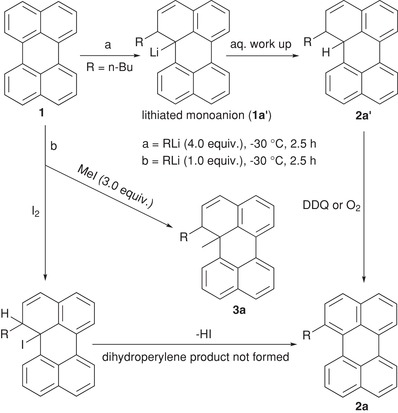
Plausible mechanistic pathway for the formation of **2a**.

To identify the side‐product a reaction of **1** with excess of *n*‐BuLi was performed (**Scheme** **2**). We were able to isolate a sufficiently large amount of the side‐product and could, therefore, identify its chemical structure: 1‐butyl‐1,12b‐dihydroperylene (**2a′**; Figures S23 and S24, Supporting Information). Furthermore, oxidation of 1‐butyl‐1,12b‐dihydroperylene (**2a′**) with 2,3‐Dichloro‐5,6‐dicyano‐*p*‐benzoquinone (DDQ) or the presence of oxygen led to the formation of 1‐*n*‐butylperylene (**2a**) (Scheme 2). We conclude that the reaction proceeds via the formation of a lithiated monoanion (**1a′**).^22^ This is supported by the fact that addition of an electrophile (MeI) to the reaction mixture of perylene and *n*‐BuLi gives methyl‐incorporated 1‐butyl‐12b‐methyl‐1,12b‐dihydroperylene (**3a**) as a sole product besides very small amounts of unreacted perylene. The structure of **3a** was confirmed by NMR and mass analysis (Figures S27—30, Supporting Information), which consequently supports the formation of **1a′** and **2a′**. Further, quenching of a reaction of *n*‐BuLi and perylene with I_2_ showed no formation of **2a′** but instead furnished *n*‐butylperylene, suggesting an addition–elimination mechanism.[[qv: 22a,b]] Overall, we argue that: (1) The lithiated monoanion (**1a′**) forms during the reaction and is particularly stable at position‐12 due to resonance and preservation of the aromatic sextet of the remaining three rings. (2) The lithiated monoanion (**1a′**) might become stabilized by solvent (THF)^23^ or alkyllithium present in the reaction. (3) The reaction proceeds via **1a′** and the major intermediate is **2a′** regardless of whether the reaction is performed with equivalent amounts or excess of *n*‐BuLi. (4) Quenching of the reaction mixture with I_2_ easily restores the aromatic system and pure alkylperylenes could be obtained in large amounts. These findings enable us to propose a mechanistic pathway and provide an excellent method to obtain 1,12‐b‐dialkylated‐1,12‐b‐dihydroperylene (**3a**) having two chiral centers, which were not known earlier. Furthermore, the reason for the interplay between reaction yield and the amount of reactants can be assigned to the differences in the amount of O_2_ present (which oxidizes **2a′** to **2a**) when performing the reaction at different scales.

**Figure 2:**
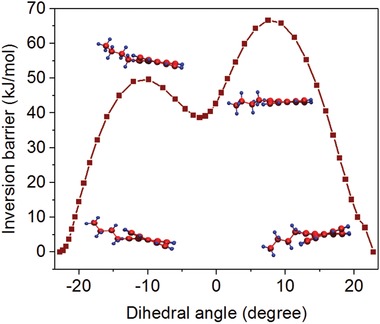
Inversion barrier of **2a** versus dihedral angle from DFT calculations.

Tetra‐ and di‐bay‐substituted PDI have shown twisting of the perylene core resulting in atropoisomers.^15^, ^16^, ^24^ Therefore, we expect the same in the case of 1‐alkylperylenes. Indeed, a twist in the perylene core is observed in single crystal X‐ray data of **2c**, which crystallizes in the monoclinic system with the space group P21/n. The unit cell contains four molecules. **2c** experiences π interactions with a longitudinal distance of 3.57 Å. Figure 1a shows two twisted **2c** molecules that are π‐stacked with a slight slipped arrangement. The shortest distance was found between the rings adjacent to the bay‐substituted ring. Figure 1b shows the extended view of the packed molecules arrangement in **2c** within a unit cell. *iso*‐Butyl groups of the π‐stacking **2c** are situated at opposite sides and the opposite direction of the saddle‐shaped perylene core. This demonstrates that even a small alkyl group at one of the bay positions can lead to a twist of the perylene core, a concept that has not been explored previously. The dihedral angles (C‐1‐C‐12a‐C‐12b‐C‐12) for **2a‐f** obtained using density functional theory (DFT) calculations (**Table** **1**) fall between 22.7° and 25° (C‐1‐C‐12a‐C‐12b‐C‐12), which are in good agreement with a measured dihedral angle of 20.64° for crystalline **2c**. For 1‐*n*‐butylperylene (**2a**), attempts to separate the two enantiomers through chiral HPLC failed. However, low‐temperature ^1^H NMR (−30 °C) revealed several peak shifts within the aromatic region and of ‐CH_2_–(*n*‐Bu) attached to the ring, indicating a locking of the conformation at −30 °C (Figure S31, Supporting Information). To rationalize the possibility for **2a‐f** to convert between left and right helices, the energy of activation for the inversion between the atropoenantiomers was computed using DFT. The dihedral angle over the bay positions was locked while geometrically optimizing the structure, yielding an energy barrier of 67 kJ mol^−1^ (694 meV) for twisting (**Figure** **2**). The activation barrier for interconversion of two enantiomers in PDIs has been shown to depend on the number and size of substituents in the bay region and an energy barrier of 106 kJ mol^−1^ is required to isolate pure atropoenantiomers at room temperature.[[qv: 24b]] Thus, the right and left helices of compound **2a** can exist in dynamic equilibrium in solution. However, as discussed before the crystal packing arrangement for compound **2c** showed the presence of enantiomeric pairs in a unit cell. Hence, it may be possible that introduction of linear or branched alkyl chains at two or all at four bay positions could potentially afford stable atropoenantiomers in solution, which is currently under investigation in our group.

**Table 1:** Measured (λ_max_) and calculated (λ_max,DFT_) absorbance maxima, molar absorptivity (ε), fluorescence quantum yield (*Φ_F_*), lifetimes (*τ_F_*), solubility in cyclohexane, and enthalpy (Δ*H_m_*) and entropy (Δ*S_m_*) of melting for as‐synthesized powders of all alkylperylenes (in brackets: material solidified from DCM). Also, calculated dihedral angle (φ), and measured melting points

Entry	λ_max_ DFT [nm]	Photophysical properties in cyclohexane				Dihedral angle [°]	Solubility [g L^‐1^]	∆*H_m_* a) [kJ mol^‐1^]	∆*S_m_* a) [J mol^‐1^ K^‐1^]	Melting point [°C]	
		λ_max_ [nm]	ε [M^‐1^ cm^‐1^]	*Φ_F_*	*τ_F_* [ns]					measuredb)	Lit.
**1**	445	434	38 500	0.94	4.01	0	0.2	30	54	278	281[[qv: 17b]]
**2a**	435	428	30 700	0.95	4.1	22.8	15.9	8 (14)	22 (41)	70 (66)	60–70[[qv: 17b]]
**2b**	435	427	30 200	0.95	4.01	23.0	12.2	17 (15)	48 (42)	82 (84)	84–85[[qv: 17b]]
**2c**	436	427	26 200	0.95	4.2	24.9	11.3	26	67	117	
**2d**	435	428	30 700	0.94	4.1	23.0	19.8	22 (26)	65 (78)	71 (73)	
**2e**	437	429	25 500	0.94	4.2	22.7	16.8	25	78	56	
**2f**	437	427	27 400	0.91	4.1	25.2	12.5	20	50	121	
**2g**	451	443	28 300	0.93	4.1	0.3	3.4	21	51	131	
**2h**	449	441	26 900	0.96	4.0	3.6	1.2	22	44	222	
**2i**	437	437	25 700	0.96	4.1	0	18.7	19	32	341	360^33^
**2j**	443	436	29 900	0.95	4.0	0	9.3	11	28	146	145–146^33^

^a)^ Calculated using Equation (1), assuming 100% crystallinity.

^b)^ Peak values. Onset and offset values are shown in Table S3, Supporting Information.

### 2.2 Photophysical Properties in Solution

**Figure 3:**
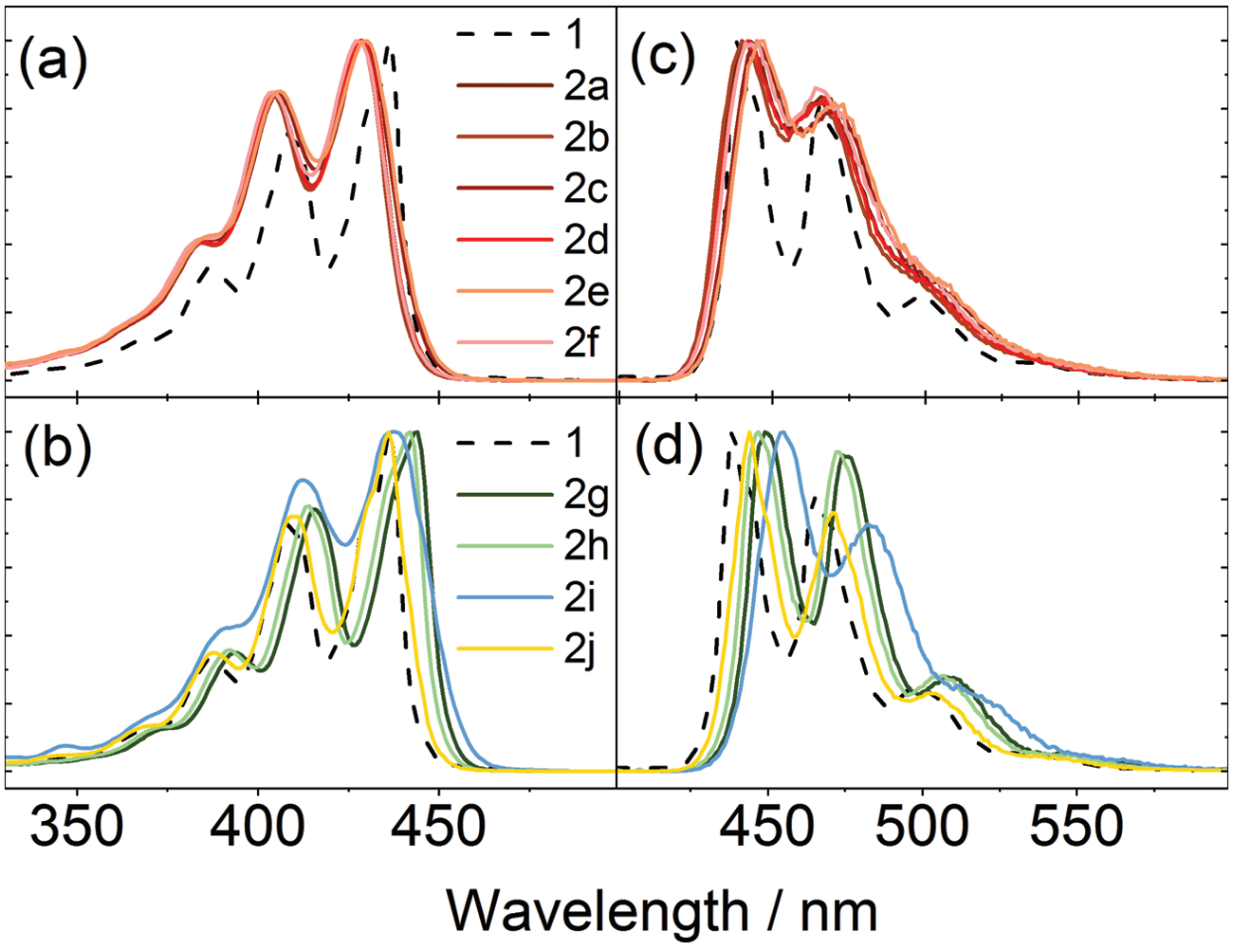
a,b) Normalized absorbance and c,d) emission spectra of a,c) 1‐alkylperylenes and b,d) 3‐ & 2‐ alkylperylenes in cyclohexane.

The photophysical properties of **2** are highly dependent on the position of substitution (**Figure** **3**). Compounds **2a‐f** exhibit an 8–9 nm hypsochromic shift of the absorbance maximum compared to unsubstituted perylene. No significant shift of the emission spectrum is observed, thus, resulting in a relatively larger Stokes shift for **2a‐f** as compared to **1**. Substituents on positions 2 and 3, on the other hand, result in no or a small bathochromic shift, respectively. Twisting the core can be viewed as a reduction of the π‐system of the molecule. With this picture in mind, a hypsochromic shift is a logical consequence when increasing the dihedral angle. Other properties that can change with a reduction of the π‐system include the strength of light matter interactions. Fluorescence quantum yield and fluorescence lifetime of **2a‐j** were therefore determined (Figure 3; Table 1). All compounds exhibit a monoexponential fluorescence decay with a lifetime in the range 4.1–4.2 ns (Figure S1, Supporting Information). The fluorescence quantum yields are roughly 90% and the molar absorptivity is about 25 000–30 000 M^−1^ cm^−1^. Thus, the strength of the light‐matter interaction, i.e., the radiative rate constant is, within error limits, the same for all derivatives. In conclusion, the twist of the perylene core results in a hypochromic shift but does not significantly affect the oscillator strength of the transition or the nonradiative rate constant deactivating the electronically excited state.

### 2.3 Properties of Neat Materials

**Figure 4:**
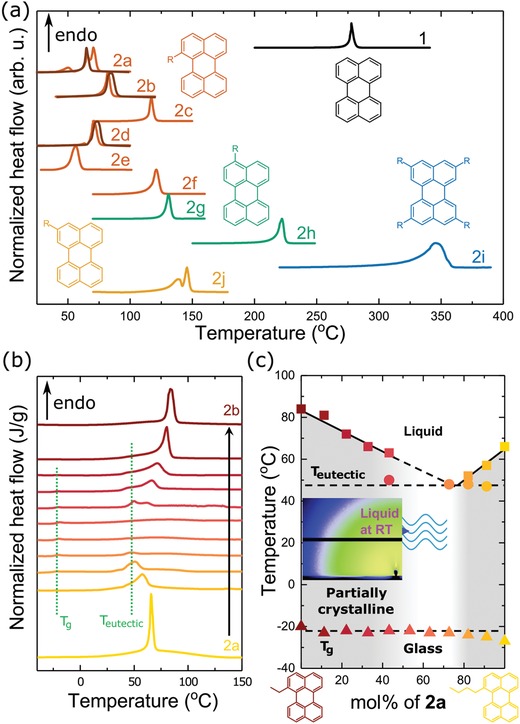
a) Differential scanning calorimetry (DSC) thermograms of **1** and **2a‐j** representing the initial heating half‐cycle of as‐synthesized powder and of material solidified from DCM (kept at −50 °C for 30 min before the scan; darker lines). The heat flow is normalized to the peak height of each melting endothermic transition. The insets show molecular structures with substituent positions. b) DSC thermograms representing the initial heating half‐cycle for blends of **2a** and **2b** solidified from DCM (kept at −50 °C for 30 min prior to the first heating scan). Glass transition (*T*_g_) and eutectic transition (*T*_eutectic_) temperatures are marked by vertical dotted lines. c) Phase diagram of mixtures of **2a** and **2b**. The liquidus (solid lines) are sketched following the peak melting temperature (squares) of each blend. The glass transition and eutectic transition (dashed lines) are sketched following the enthalpy relaxation peak associated with the glass transition (triangles) and the peak temperature for the eutectic point (circles), respectively. The lightly gray area indicates the region where the blend remains liquid. The gray area indicates the region where the blend is partially crystalline and the remaining area indicates the region where the blend is a liquid or a glass. The inset shows a grazing‐incidence wide angle X‐ray scattering (GIWAXS) diffractogram of a 1:1 molar ratio blend of **2a** and **2b** kept at room temperature for 1 month.

To examine if a liquid chromophore is achieved through substitution, the melting temperatures were assessed using differential scanning calorimetry (DSC; **Figure** **4**a). We found that even a small functional group, such as an ethyl substituent at position 1 (**2b**), lowers the melting point significantly compared to **1**, i.e., *T_m_* = 82 °C instead of 278 °C. Not surprisingly, these molecules are nearly an order of magnitude more soluble than unsubstituted **1** (in cyclohexane; Table 1). The melting point is given by the relative change of enthalpy and entropy, ∆*H_m_* and ∆*S_m_*, upon melting:

(1) $$T_{m}=\Delta H_{m}/\Delta S_{m}$$

Changes in the type, number, and position of substituents can have subtle influences on both ∆*H_m_* and ∆*S_m_* (cf. Table 1), which prevent us from providing a general rationale for the measured melting points.

To assess the photophysical properties of pristine **2**, thin films were fabricated by spin coating. All compounds produced films that contain small crystals. However, films of **2a‐f** were generally easier to make and reproduce. The absorption properties of the films, even though slightly broadened, resemble those in dilute solution (Figure S2, Supporting Information). The fluorescence quantum yield from the films was low and below the limit that can be accurately measured for all compounds. Furthermore, both monomeric and excimer emissions were detected, indicating a heterogeneous system containing crystals, as well as more amorphous regions.

### 2.4 Liquefying through Thermodynamic Mixing

**Figure 5:**
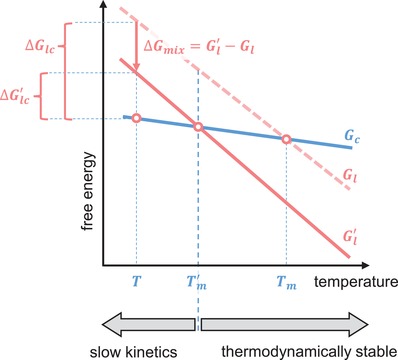
Illustration of the impact of mixing on the melting temperature, which decreases from *T_m_* to $T_{m}^{\prime }$, and driving force for crystallization at temperature *T*, which decreases from Δ*G*_lc_ to $\Delta G_{\mathrm{lc}}^{\prime }$, where *G_c_*, *G_l_*, and $G_{l}^{\prime }$ are the Gibbs free energy of the crystalline state (*G_c_* of only one component is shown for clarity), of the single‐component liquid, and of the multicomponent liquid, respectively. Above $T_{m}^{\prime }$, the multicomponent liquid is thermodynamically stable, whereas below $T_{m}^{\prime }$ the crystallization kinetics are slowed due to a reduced $\Delta G_{\mathrm{lc}}^{\prime }$.

To our knowledge, no perylene derivative has been reported in a liquid state at room temperature. Despite the general reduction in melting point of the molecules reported above, none of them has a melting temperature below 50 °C. Most of these molecules do not crystallize immediately when quenched from the liquid phase and show no crystallization exotherm during the subsequent DSC cooling thermogram. It is, however, unlikely that any of these molecules remain amorphous at room temperature for an extended time above their respective glass transition temperature (*T_g_* ≈ −25 °C for **2a**, **2b**, and **2d**; Figure S33, Supporting Information). Since all perylene derivatives with substitutions at position 1 exhibit more or less identical photophysical properties, mixing of two or more of these molecules should not significantly influence the photophysical properties of the combined system in solution. Mixing can, however, prevent crystallization, which impacts the photophysics of the system. Indeed, mixing of several materials near equimolar composition is used for producing so‐called high entropy alloys, amorphous metals (“metallic glasses”),^25^ as well as amorphous drugs.^26^ It is also found that mixing small organic molecules with similar chemical structures, such as fullerene C_60_ and C_70_ or rubrene and 9,10‐diphenylanthracene, prevents or at least slows down the crystallization, resulting in a liquid that can be frozen below *T_g_*.^26^, ^27^ In the limit of an ideal or regular solution, mixing of two (or more) components increases the entropy of the liquid state by:

(2) $$\Delta S_{mix}=-Nk_{B}\sum_{i}\phi_{i}ln\phi_{i}$$

where φ_*i*_ is the molar fraction of component *i* and *N* is the total number of molecules. For an ideal solution, for which the enthalpy of mixing Δ*H_mix_* is zero, the Gibbs free energy of the solution is hence decreased by $\Delta G_{mix}=\;G_{l}^{\prime }-G_{l}=-T\Delta S_{mix}$. As a result, the melting temperature decreases from *T_m_* to $T_{m}^{\prime }$ and the driving force for crystallization at *T* decreases from Δ*G_lc_* to $\Delta G_{lc}^{\prime }$ (**Figure** **5**). The former implies that there is a range of temperatures below the original *T_m_*, where the mixed liquid is thermodynamically stable. The depression of the driving force reduces both the rate of crystal nucleation and growth below $T_{m}^{\prime }$, meaning that the blend components will crystallize more slowly. For a nonideal solution, we must distinguish between Δ*H_mix_* < 0, e.g., due to π interactions, and Δ*H_mix_* > 0, which will augment or oppose the *T*Δ*S_mix_* term, respectively.

**Figure 6:**
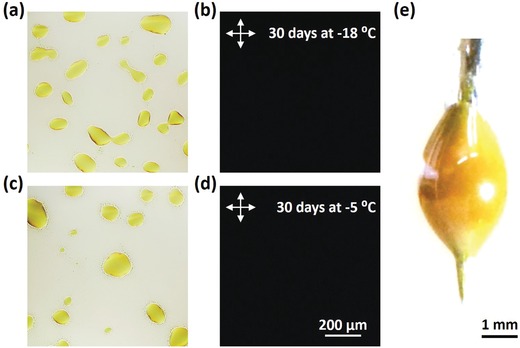
a,c) Bright‐field and b,d) cross‐polarized optical micrographs of a drop cast 1:1 molar ratio blend of **2a** and **2b** after keeping at a,b) −18 °C and c,d) −5 °C for 30 days. e) A photograph of a liquid droplet comprising a 1:1 molar ratio blend of **2a** and **2b** on a needle tip at room temperature.

Here, we exploit this configurational entropy effect and study blends of **2a**:**2b** (Figure 4), as well as **2a**:**2d** and **2b**:**2d** (Figure S32, Supporting Information). All three mixtures show indeed a reduction of the melting temperature and the enthalpy of melting relative to the neat components. Binary phase diagrams of the investigated mixtures reveal no clear endothermic melting transition, as the liquidus lines merge for stoichiometries around 50 mol% (corresponding to 1:0.91 wt:wt for **2a:2b**). This indicates that for equimolar blends, for which the configurational entropy of mixing is maximal (cf. Equation (2)), the ability of the material to crystallize is impaired.^28^ The amorphous nature is further supported by the more pronounced glass transition at −25 °C in blends with a molar ratio close to 1:1, as compared to the neat materials (glass transitions can only be observed when a large fraction of the material remains amorphous,^29^ but are present in all samples to some extent). We conclude that the two blend components in (close to) equimolar mixtures do not tend to crystallize below the eutectic temperature, remain liquid (even at room temperature), and are frozen into a glassy state upon further cooling below *T_g_* ≈ −25 °C. We note that the crystalline phase can be absent because the liquid state is thermodynamically stable or, at lower temperatures, since crystallization is kinetically suppressed, i.e., the rate of nucleation and/or growth of both components is sufficiently reduced so that crystallization is exceedingly slow (Figure 5). To explore whether the liquids crystallize if given more time, we kept a drop cast equimolar blend of **2a** and **2b** at −18 °C, −5 °C, and room temperature for 1 month. We chose these temperatures because nucleation is greatly promoted right above *T_g_* (for the case of −18 °C) and the total crystallization rate is maximized at temperatures approximately half‐way between the *T_g_* and *T_eutectic_* ≈ 50 °C (for samples kept at −5 °C and room temperature).^30^ In the bright‐field and polarized optical micrographs of the thin films kept at −18 °C and −5 °C, one can clearly discern bright droplets corresponding to dewetting of the materials on the glass substrate (**Figure** **6**). More importantly, the images show no sign of crystal formation. The liquid nature of the mixture was further confirmed by grazing incidence wide angle X‐ray scattering (GIWAXS) for the material kept at room temperature for 1 month, showing no crystalline diffraction (inset in Figure 4c).

The fluorescence quantum yield of the liquid mixture of **2a**:**2b** is relatively high, i.e., 22%, and the average excited state lifetime is long, i.e., 27 ns. These values are significantly larger than those of solid neat films. The envelope of emission, however, does not resemble that of perylene in dilute solution, but rather that of excimer emission (Figure S5, Supporting Information). Given the short distance between chromophores, emission from an excimeric state is not surprising. Furthermore, in applications that require liquid chromophores, there is also a demand for close packed molecules, maximizing the chromophore concentration. Obtaining a liquid chromophore at the expense of a vastly increased molecular weight is, therefore, not desirable. Compounds **2a** and **2b** have very small substituents, and on average, increase in the molar weights of the two perylene derivatives is a mere 17%. This is, to the best of our knowledge, the smallest molecular weight of the liquefying side chains of any liquid chromophore.

Crucially, the materials obtained here serve as a prototype for a general pathway for obtaining liquid chromophores with minimal weight increase relative to the parent compounds. Specifically, we have demonstrated that one can stabilize a liquid state by blending photophysically identical chromophores with small but different aliphatic substituents. This approach relies on an increase in the configurational entropy of the system by mixing and is, thus, based on a universal thermodynamic principle that transcends the underlying chemistry.

## 3 Conclusions

A set of alkylated perylenes was synthesized and their physical and photophysical properties were examined. As desired, the introduction of alkyl groups did not bring a drastic change in the photophysical properties. Substitution with linear or branched alkyl groups at position 1 shows a remarkable decrease in melting point due to twisting of the molecular core. Furthermore, perylene blends that are liquid at room temperature were achieved by mixing two derivatives with similar photophysical properties. The resulting materials represent, to the best of our knowledge, the liquid chromophores with the smallest weight increase due to solubilizing substituents (17%). Our analysis suggests that the approach of chemical modification in combination with thermodynamic mixing is general. It should, therefore, be applicable to any parent compound, yielding liquid chromophores with minimal weight increase.

## 4 Experimental Section

*Spectroscopy*: Absorbance was measured using a Lambda 650 (Perkin Elmer) spectrophotometer. Fluorescence measurements for solution samples were done with a Spex Fluorolog (JY Horiba) spectrofluorometer. Fluorescence quantum yields in solution were determined relative to perylene as an average of two samples. The excitation wavelength for the quantum yield measurements was 360 nm and the absorbance was around 0.03–0.05 at the excitation wavelength. Fluorescence lifetimes in cyclohexane were determined on a time‐correlated single‐photon counting setup using a PicoQuant laser diode (405 nm) and a micro‐channel plate (MCP) detector. Fluorescence measurements for mixed films were measured using an FLS1000 (Edinburgh Instruments) spectrophotometer equipped with an integrating sphere for quantum yield determination. The quantum yield was determined as an average of four measurements (turning plate 90° between each measurement) against the reference (clean glass plate) measured two times (tuning plate 180°).

*Spin Coating*: A total of 1 mM solutions of alkylperylenes in cyclohexane were spin coated (Laurell) on glass substrates (25 × 25 mm), which were precleaned by sonication for 15 min in alkaline solution (0.5% of Hellmanex in distilled water), then rinsed with water and sonicated for 1 h in water and ethanol, respectively. The cleaned glass substrates were dried in an oven overnight before use.

*Drop Casting*: Pristine samples or mixtures of 2a and 2b (3.5 mM in DCM) were dropcasted on glass substrates (12.5 × 12.5 mm), which were precleaned as above. The films were vacuum dried overnight.

*Differential Scanning Calorimetry*: DSC measurements were carried out with a Mettler Toledo DSC2. Mettler 20 µL Al crucible light sample pans were used; the samples weighed around 5 mg. DSC measurements of neat alkylperylenes were carried out with as‐synthesized powder. For the blends, prior to DSC, the material was dissolved in DCM (10 g L^−1^), dried and then taken up with tissue paper, which was placed into DSC pans. Samples were first cooled to −50 °C at a rate of −10 °C min^−1^ and kept isothermal for 30 mins, followed by two heating and cooling scans between −50 °C and 200 °C at 10 °C min^−1^; melting points were extracted from first heating scans and glass transition temperatures were extracted from second heating scans.

*GIWAXS*: Grazing incidence wide‐angle X‐ray scattering (GIWAXS) measurements were performed at the D‐line, Cornell High Energy Synchrotron Source (CHESS) at Cornell University. An X‐ray beam with a wavelength of 1.162 Å was directed at the thin film at an incident angle of 0.15°. A Pilatus 200 k detector located at 177.2 mm from the sample was used to record the image during 3 s of exposure.

*Optical microscopy*: Transmission optical micrographs were recorded with a Zeiss Axio Scope A1 equipped with a pair of crossed polarizers.

*DFT Calculations*: Molecular configurations were relaxed and absorption spectra were calculated within the framework time‐dependent density functional theory employing the B3LYP functional as implemented in the NWChem suite.^31^ All of these calculations were carried out using a 6−311+G*basis set.^32^ The inversion barrier of **2a** in vacuum was calculated using the nudged elastic band method at the B3LYP/6−31+G(d) level. The molecular structures of the initial and final states of fragmentation were optimized using DFT B3LYP/6−31+G(d).

[CCDC 1836678 contains the supplementary crystallographic data for this paper. These data can be obtained free of charge from The Cambridge Crystallographic Data Centre via www.ccdc.cam.ac.uk/data_request/cif.]

## Conflict of Interest

The authors declare no conflict of interest.

## Supporting information

Supplementary
