# Supplementary material for: A Record Chromophore Density in High‐Entropy Liquids of Two Low‐Melting Perylenes: A New Strategy for Liquid Chromophores
Source: Adv Sci (Weinh). 2019 Jan 15;6(4):1801650. doi: 10.1002/advs.201801650 (PMC6382313; doi:10.1002/advs.201801650)
Supplement: Supplementary file 1 — Supplementary [file ADVS-6-1801650-s001.pdf]

Copyright WILEY-VCH Verlag GmbH & Co. KGaA, 69469 Weinheim, Germany, 2018.

ADVANCED SCIENCE
Open Access

Supporting Information

for *Adv. Sci.,* DOI: 10.1002/advs.201801650

A Record Chromophore Density in High-Entropy Liquids of Two Low-Melting Perylenes: A New Strategy for Liquid Chromophores

*Khushbu Kushwaha, Liyang Yu, Kati Stranius, Sandeep Kumar Singh, Sandra Hultmark, Muhammad Naeem Iqbal, Lars Eriksson, Eric Johnston, Paul Erhart, Christian Müller, and Karl Börjesson**

Copyright WILEY-VCH Verlag GmbH & Co. KGaA, 69469 Weinheim, Germany, 2016.

Supporting Information

Record chromophore density in a high-entropy liquid of two low-melting perylenes: A new strategy for liquid chromophores

Khushbu Kushwaha, Liyang Yu, Kati Stranius, Sandeep Kumar Singh, Sandra Hultmark, Muhammad Naeem Iqbal, Lars Eriksson, Eric Johnston, Paul Erhart, Christian Müller and Karl Börjesson*

Table of Contents

		Page
1.	Spectroscopy	2-3
2.	GIWAXS images of compounds **2d, 2f, 2j** and **2h**	4
3.	Synthesis and characterization of compounds **2a-h**	5-25
4.	Synthesis and characterization of compounds **2i-j**	26-31
5.	Experiments related to mechanistic pathway	32-43
6.	Low temperature NMR for **2a**	44
7.	Single crystal X-ray of **2c**	45-48
8.	DSC of **2a**:**2d** and **2b**:**2d** mixtures	49
9.	References	51

1. Spectroscopy

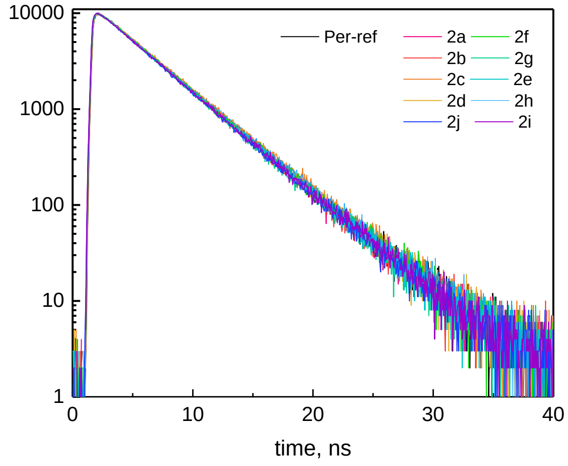

Figure S1. Fluorescence decays for compounds **2a-j** in cyclohexane, excited at 405 nm and monitored at the fluorescence maximum.

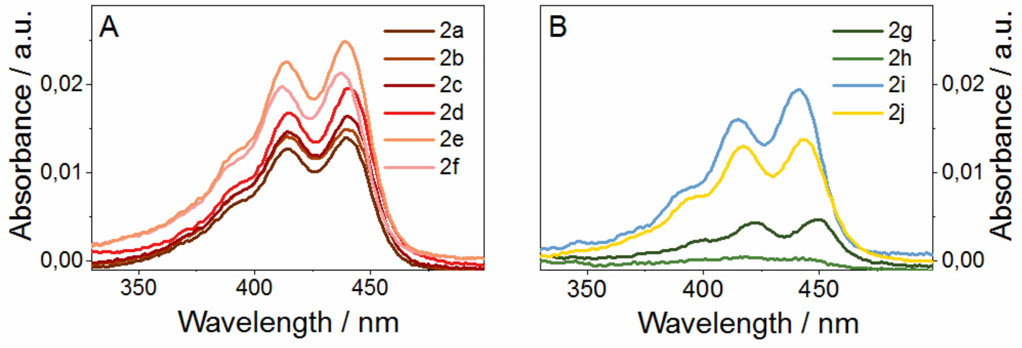

Figure S2. Absorbance spectra of pristine 1-alkylperylenes (A) and 3- & 2- alkylperylenes (B) films spin-coated on a glass plate using 1 mM solution in cyclohexane.

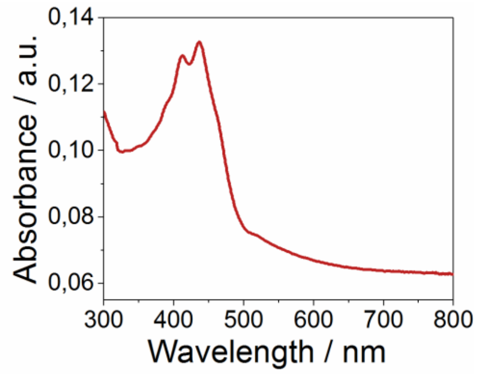

Figure S3. Absorption spectrum of pristine mixed sample (**2a**:**2b**; 1:1) film drop-coated on a glass plate.

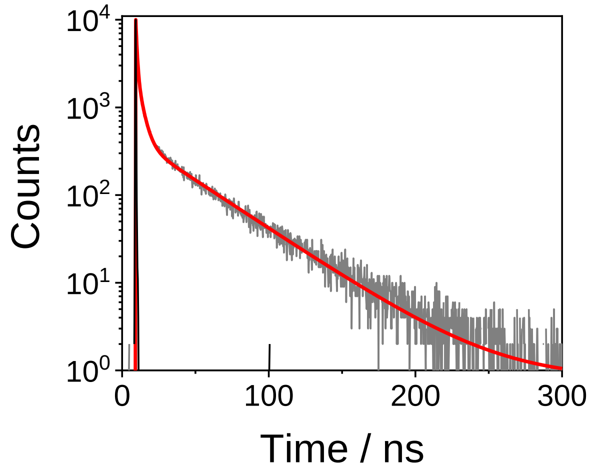

Figure S4. Emission decay at 530 nm of mixed film excited at 405 nm. Red line shows the 3-exponential fitting giving τ_1=0.8 ns, τ_2=3.8 ns and τ_3=39 ns.

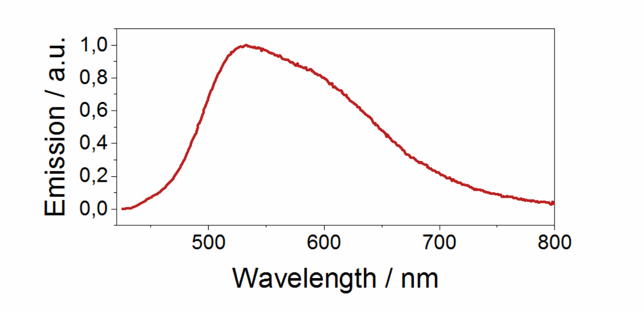

Figure S5. Emission spectrum of perylene blend (**2a**:**2b**; 1:1 wt:wt) drop-cast on a glass plate.

Table S1. Quantum Yield for mixed sample 1:1 blend of 2a and 2b

Measured QY for mixed film excited at 405 nm. QY were measured for mixed sample 4 times by turning the sample 90° between measurements. The reference (clean glass-plate) was measured 2 times by turning sample 180°.

	ref 1	ref 2
1	25.26	26.33
2	18.14	17.91
3	18.99	18.94
4	23.34	24.16

2. GIWAXS images

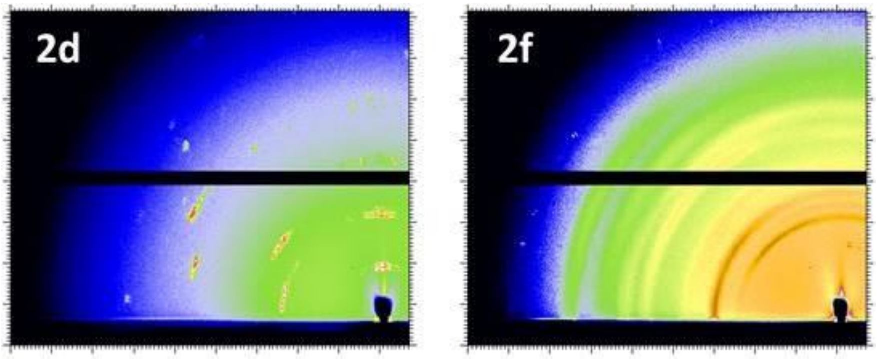

Figure S6. GIWAXS image of drop cast films containing **2d** and **2f**. The notable difference between these two images suggest distinguishable packing styles.

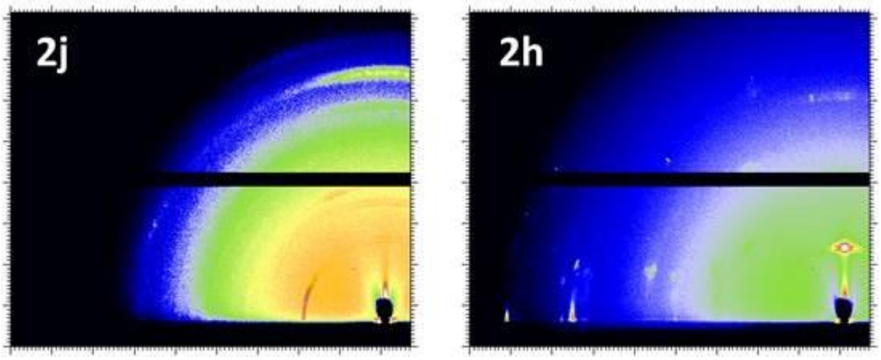

Figure S7. GIWAXS image of drop cast films containing **2j** and **2h**. The notable difference between these two images suggest distinguishable packing styles.

3. Synthesis and characterization of compounds 2a-h

Methods and materials: All alkyl lithium reagents were purchased from Sigma-Aldrich Chemical Co. Dry THF was obtained through solvent purifying machine (MBRAUN SPS 800) and used as such for carrying out reactions. Products were purified by column chromatography using basic alumina (Al_2O_3) purchased from Merck. ^{1}H (^{13}C) NMR spectra were recorded at 400/500 (100/126) MHz on a Bruker spectrometer using $CDCl_3$ as solvent. *J* values are given in Hertz (Hz) and chemical shifts are given in ppm. The ^{13}C and ^{1}H chemical shifts were referenced to residual solvent signals at δ_C 77.00 and δ_H at 7.26 for $CDCl_3$. 2D Experiments (COSY, NOSEY, HSQC and HMBC) were carried out for some compounds (**2b** and **3a**) and data are given for supporting the structure of the final compounds. X-ray analysis data is provided for compound **2c**. GC-MS (Agilent 7820A, column HP-5MS 30 mm x 250 µm x 0.25 µm) were recorded for all compounds. FT-IR (ATR) were carried out on Perkin-Elmer Frontier. For high resolution mass spectrometric analysis, all samples dissolved in DCM to approximately 1 mg/mL. Then 20 µL of sample was dissolved in 1.5 mL of MeCN for injection. All samples were analyzed by APCI source in positive mode.

General experimental procedure (I) for the synthesis of 1-alkylperylenes (2a-h): A schlenk flask was charged with Perylene (0.5 mmol-1.0 mmol, 1.0 equiv.), connected to a schlenk line and three vacuum/nitrogen cycles were performed. Then, dry THF was added to the flask preparing approximately 0.025 M solution, under nitrogen atmosphere and the temperature was reduced to -30 °C. To this, alkyllithium (1.1-1.5 equiv.) was slowly added during 1.5 h and the temperature was maintained at -30 °C for an additional 1 h. Afterwards, the reaction mixture was poured onto dry ice in small portions. The organic layer was separated, diluted with DCM, followed by washing with saturated NH_4Cl solution. The combined organic layer was dried using sodium sulphate, filtered and the filtrate was evaporated under vacuum. The crude product was purified by column chromatography on basic alumina using cyclohexane:toluene solvent system.

General experimental procedure (II) for the synthesis of 1-alkylperylenes (2a-h): A schlenk flask was charged with Perylene (252 mg, 1.0 mmol), connected to a schlenk line and three vacuum/nitrogen cycles were performed. Then, dry THF (40 mL) was added to the flask, preparing approximately 0.025 M solution, under nitrogen atmosphere and the temperature was reduced to -30 °C. To this, alkyllithium (1.1-1.5 equiv.) was slowly added during 1.5 h and the temperature was maintained at -30 °C for an additional 1 h. Afterwards, reaction was quenched

with I_2 (3.0 equiv.) and continued to stir for 15 minutes. The contents was poured onto a saturated solution of sodium thiosulfate. The organic layer was separated, followed by washing with water. The combined organic layer was dried using sodium sulphate, filtered and the filtrate was evaporated under vacuum. The crude product was purified by column chromatography on basic alumina using cyclohexane:toluene solvent system.

Synthesis of 2a in large amounts: upscaling of reaction

A two neck 250 mL round bottom flask was charged with Perylene (1.0 g, 4.0 mmol, 1.0 equiv.), and three vacuum/nitrogen cycles were performed. Then, 160 mL (approx.) of dry THF was added *via* cannula to the schlenk-flask under nitrogen and the temperature was reduced to -30 °C. To this, *n*-BuLi (2.3 mL, 5.1 mmol, 1.3 equiv.) was slowly added during 1.5 h and the temperature was maintained at -30 °C for an additional 1 h. Afterwards, reaction was quenched with I_2 (3.0 g, 3.0 equiv.) and continued to stir for 15 minutes. Followed by usual workup and column chromatography on basic alumina using cyclohexane:toluene (9:1) as an eluent 57 % (689 mg) of **2a** was obtained. Later fractions showed formation of 3-*n*-butylperylene, indicated by ^1H NMR but it could not be isolated in sufficient purity.

3. Synthesis and characterization of 2a-j

1-*n*-Butylperylene (2a)

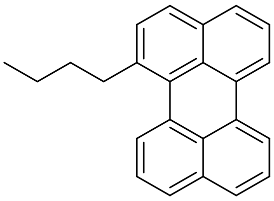

2a

According to the general procedure (I), Perylene (**1a**) (126 mg, 0.5 mmol), *n*-BuLi (2.2 M in hexane, 0.65 mmol, 0.30 mL) were reacted in THF (20 mL) under nitrogen at -30 °C for 2.5 h. Aqueous work up with ammonium chloride solution followed by column chromatography using cyclohexane:toluene (9:1) as an eluent afforded 1-*n*-butylperylene (**2a**) as a yellow solid. mp (DSC): 63 °C (onset); Lit[1] 60-70 °C, yield (100 mg, 65%), R_f = 0.48 in cyclohexane:toluene (9:1).

^1H NMR (400 MHz, $CDCl_3$): δ = 1.04 (t, 3H, J = 7.6 Hz, $CH_2CH_2CH_2C\mathbf{H_3}$), 1.55 (sextet, overlapped with H_2O peak, 2H, J = 7.6 Hz, $CH_2CH_2C\mathbf{H_2}CH_3$), 1.95-1.91 (m, 2H, $CH_2C\mathbf{H_2}CH_2CH_3$), 3.20-3.16 (m, 2H, $C\mathbf{H_2}CH_2CH_2CH_3$), 7.56-7.46 (m, 4H), 7.76-7.65 (m, 4H),

7.87 (dd, 1H, J_1 = 7.6 Hz, J_2 = 1.0 Hz), 8.17 (2 × dd, 2H, J_1 = 7.6 Hz, J_2 = 1.2 Hz). **^{13}C NMR (100 MHz, $CDCl_3$):** δ = 14.0 (<u>**C**</u>H_3, *n*-Bu), 23.0 (<u>**C**</u>H_2CH_3, *n*-Bu), 33.2 (Ar-CH_2<u>**C**</u>$H_2CH_2CH_3$), 35.7 (Ar-<u>**C**</u>$H_2CH_2CH_2CH_3$), 120.0, 120.8, 125.6, 125.8, 126.2, 126.4, 127.0, 127.02, 127.2, 127.3, 129.5, 130.0, 130.7, 130.9, 131.1, 131.4, 132.6, 134.2, 138. **IR (ATR cm^{-1}):** ν = 3048, 2953, 2924, 2858, 820, 769. **GC-MS** (retention time = 13.01 min) m/z: 308 (M^+), 293.1 (M^+ - CH_3). **HRMS (APCI):** *m/z* calcd for $(C_{24}H_{20}+H)^+$: 309.1638; found: 309.1642.

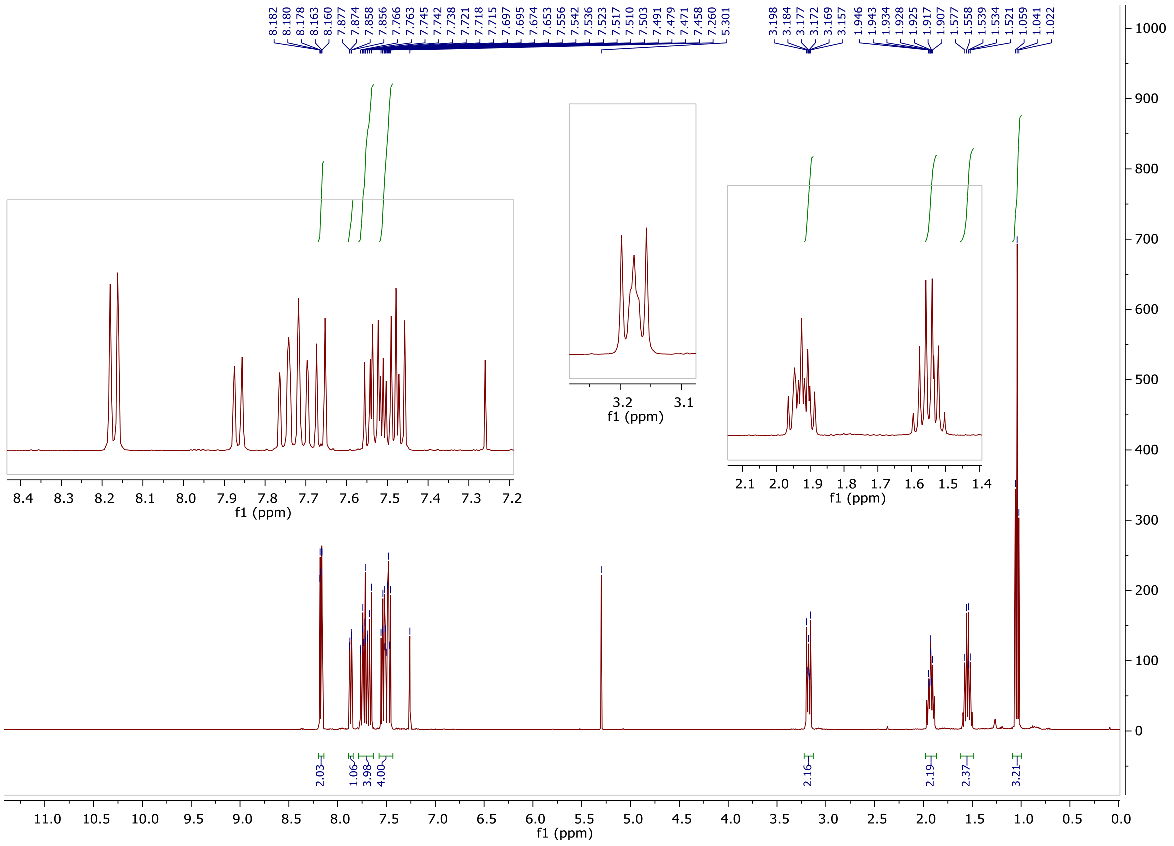

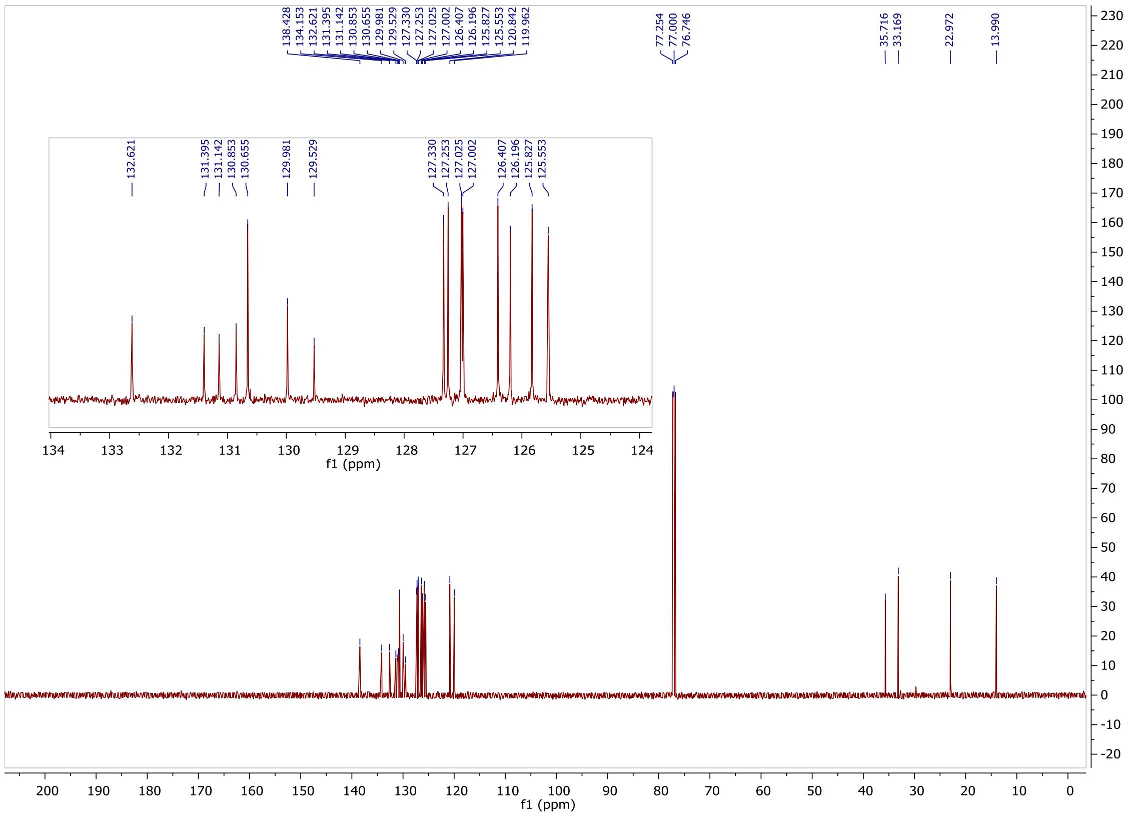

Figure S8. ^{1}H (400 MHz) and ^{13}C (100 MHz) NMR spectra of **2a** in $CDCl_3$.

1-Ethylperylene (2b)

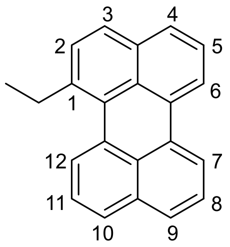

2b

According to the general procedure (I), Perylene (**1a**) (1.0 mmol, 252 mg), EtLi (0.5 M in benzene, 1.5 mmol, 3.0 mL) were reacted in THF (40 mL) under nitrogen at -30 °C for 2.5 h. Aqueous work up with ammonium chloride solution followed by column chromatography using cyclohexane:toluene (9:1) as an eluent afforded 1-ethylperylene (**2b**) as a yellow solid. mp (DSC): 78 °C (onset); Lit[1] 84-85 °C, yield (64 mg, 23%). R_f = 0.46 in cyclohexane:toluene (9:1).

According to the general procedure (II), Perylene (**1a**) (1.0 mmol, 252 mg), EtLi (0.5 M in benzene, 1.5 mmol, 3.0 mL) were reacted in THF (40 mL) under nitrogen at -30 °C for 2.5 h. Reaction was quenched with I_2 (3.0 equiv.) followed by work up and column chromatography

using cyclohexane:toluene (9:1) as an eluent to afford 1-ethylperylene (**2b**) as a yellow solid. Yield (101 mg, 36%).

^{1}H NMR (500 MHz, CDCl$_3$): δ = 1.55 (t, 2H, J = 7.6 Hz, CH$_2$C**H$_3$**), 3.23 (q, 3H, J = 7.6 Hz, C**H$_2$**CH$_3$), 7.55-7.47 (m, 4H, H-2, H-5, H-8, H-11), 7.76-7.67 (m, 4H, H-3, H-4, H-9, H-10), 7.89 (dd, 1H, J_1 = 7.5 Hz, J_2 = 1.0 Hz, H-12), 8.18 (dd, 2H, J_1 = 7.5 Hz, J_2 = 1.1 Hz, H-6, H-7). **^{13}C NMR (126 MHz, CDCl$_3$):** δ = 15.6 (CH$_2$**C**H$_3$), 28.9 (**C**H$_2$CH$_3$), 120.0, 120.9, 125.6, 125.9, 126.2, 126.4 , 127.05, 127.14, 127.25, 127.3, 129.5, 129.91, 129.97, 130.1 , 130.9, 131.1, 131.3, 132.6, 134.1, 139.6. **IR (ATR cm^{-1}):** ν = 3053, 2958, 2930 (C-H), 822, 768. **GC-MS** (retention time = 11.599 min) m/z: 280.1 (M$^+$). **HRMS (APCI):** *m/z* calcd for $(C_{22}H_{16}+H)^+$: 281.1325; found: 281.1336.

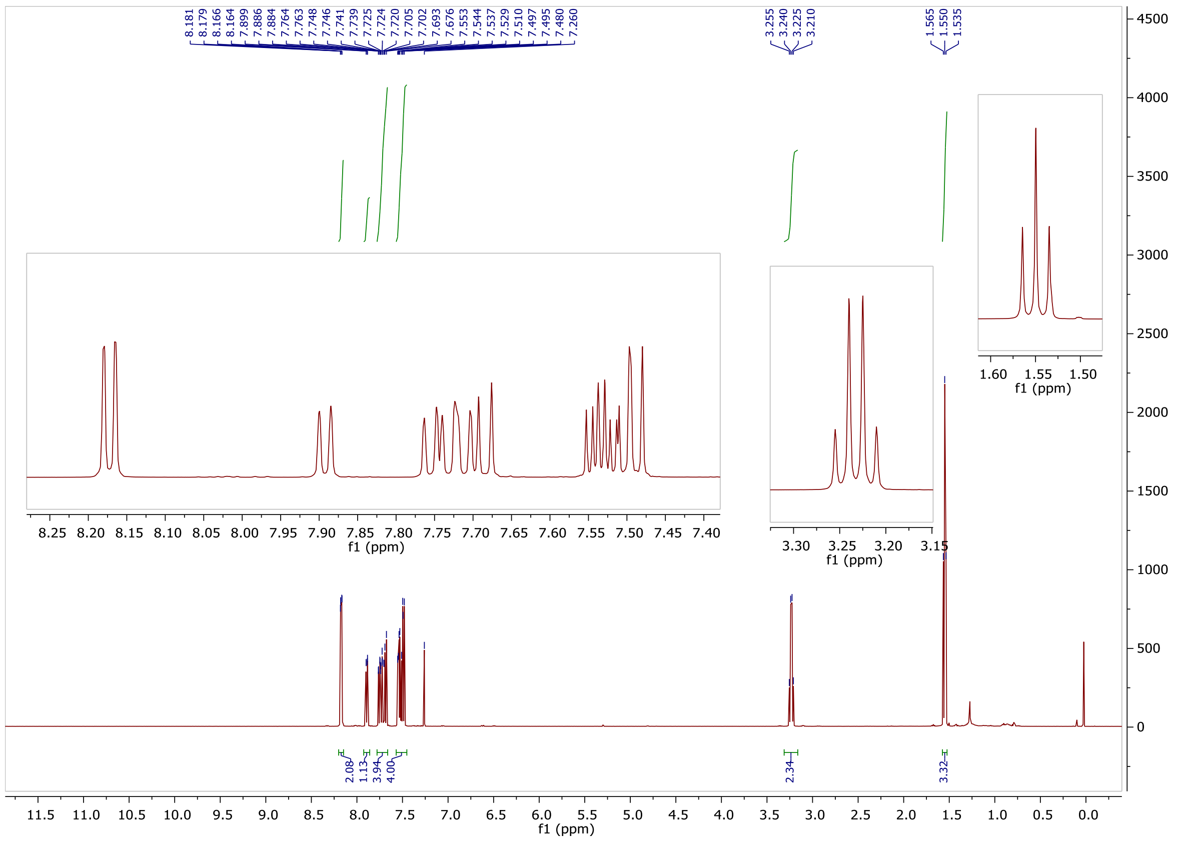

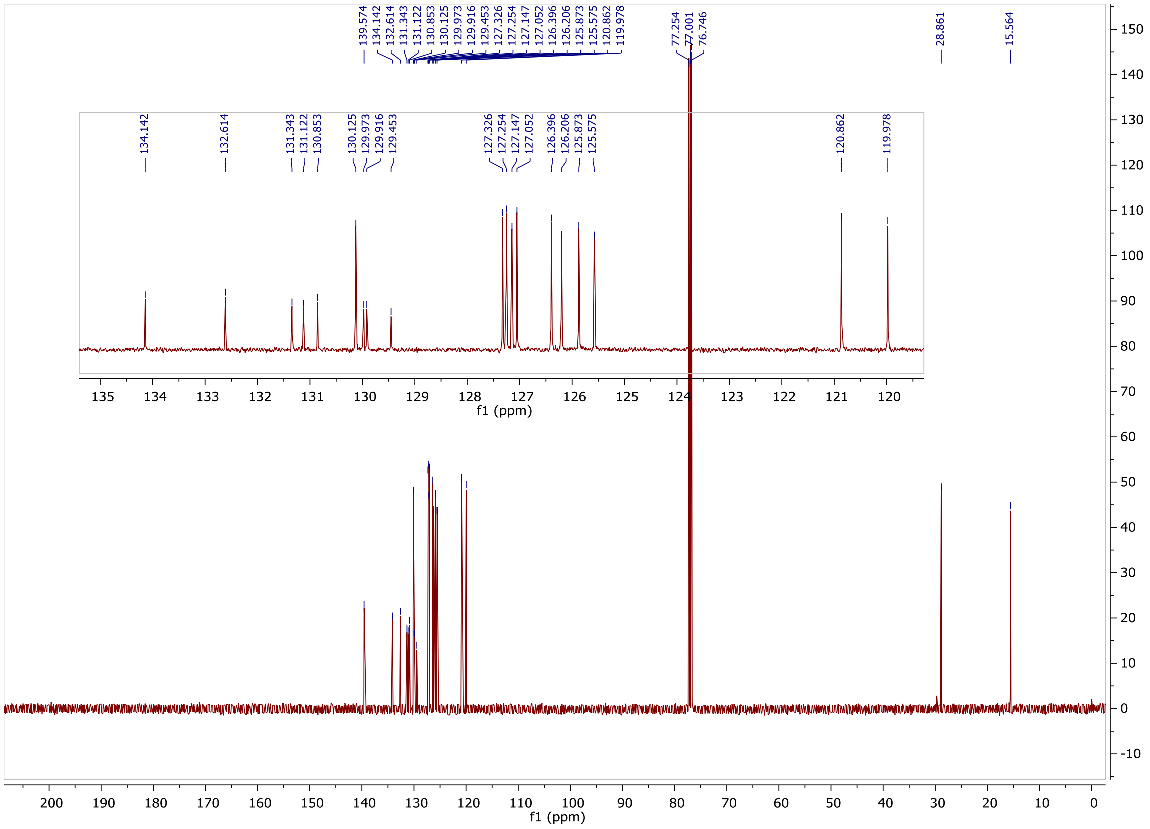

Figure S9. ^{1}H (500 MHz) and ^{13}C (126 MHz) NMR spectra of **2b** in $CDCl_3$.

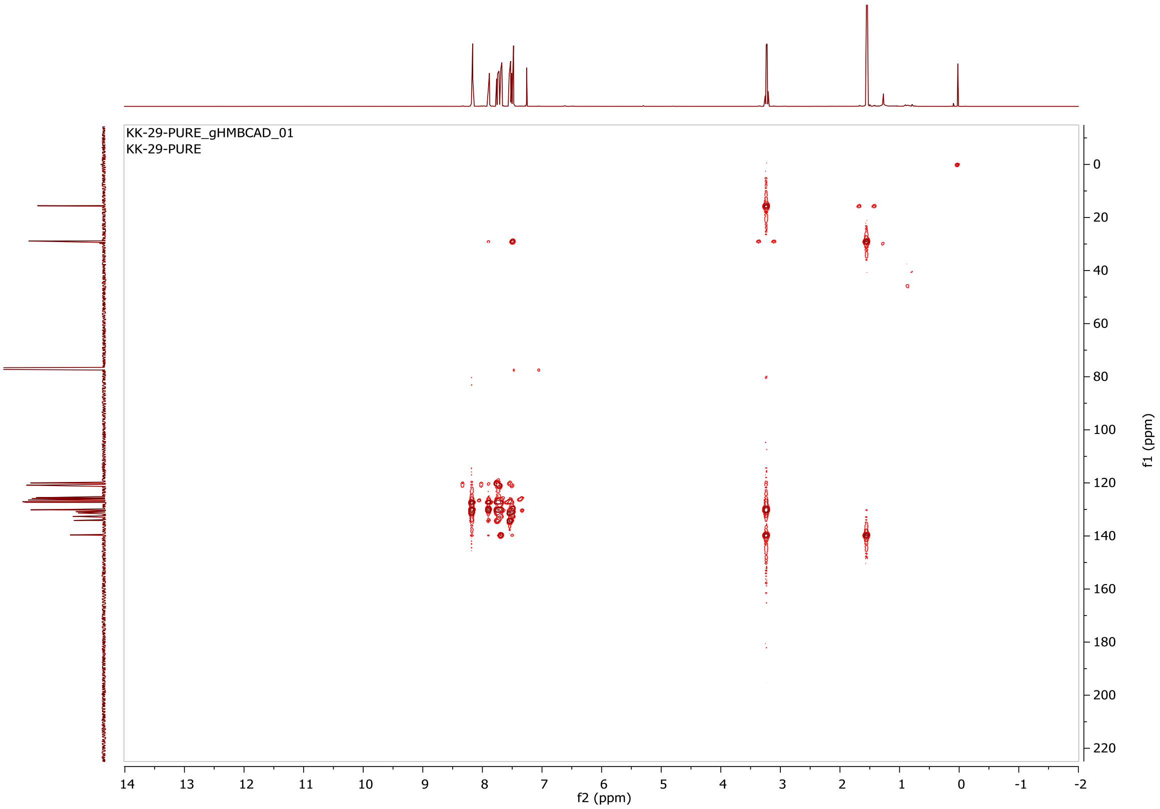

Figure S10. HMBC 2D NMR spectra of **2b** in $CDCl_3$.

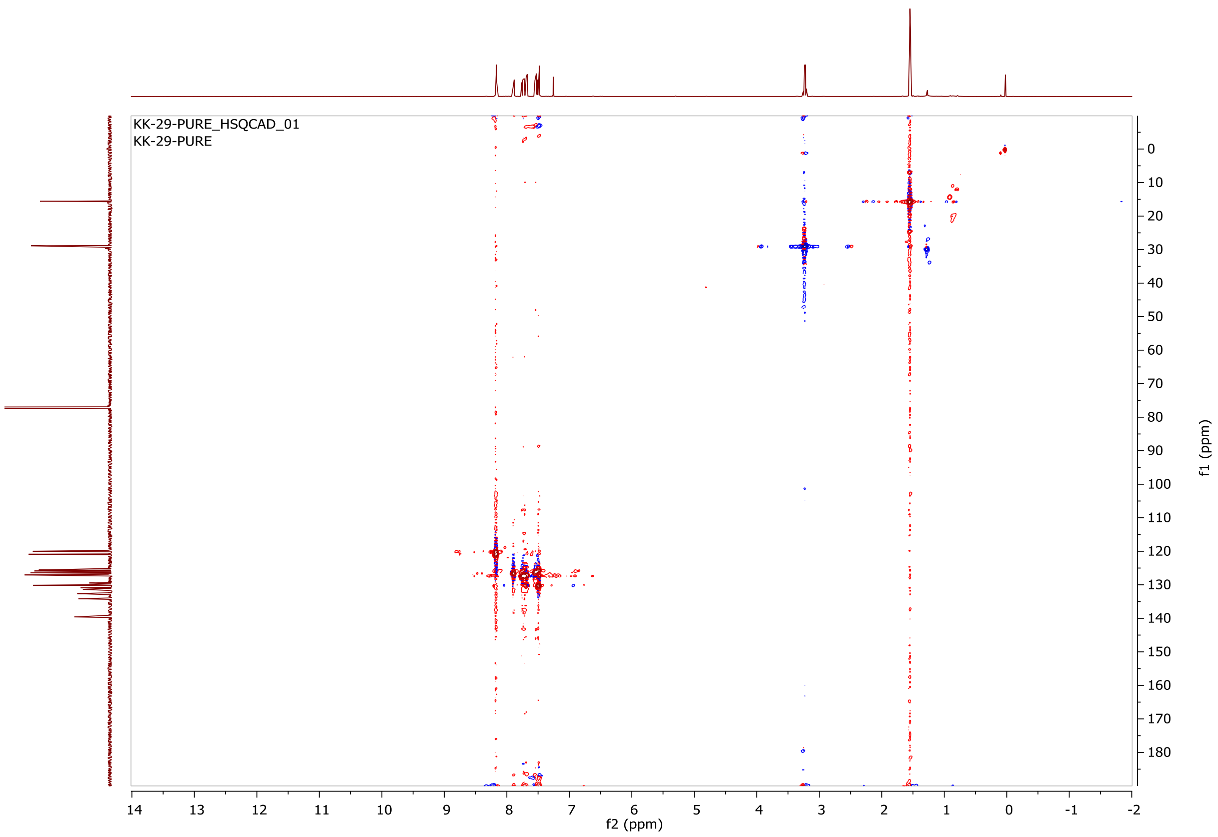

Figure S11. HSQC 2D NMR spectra of **2b** in $CDCl_3$.

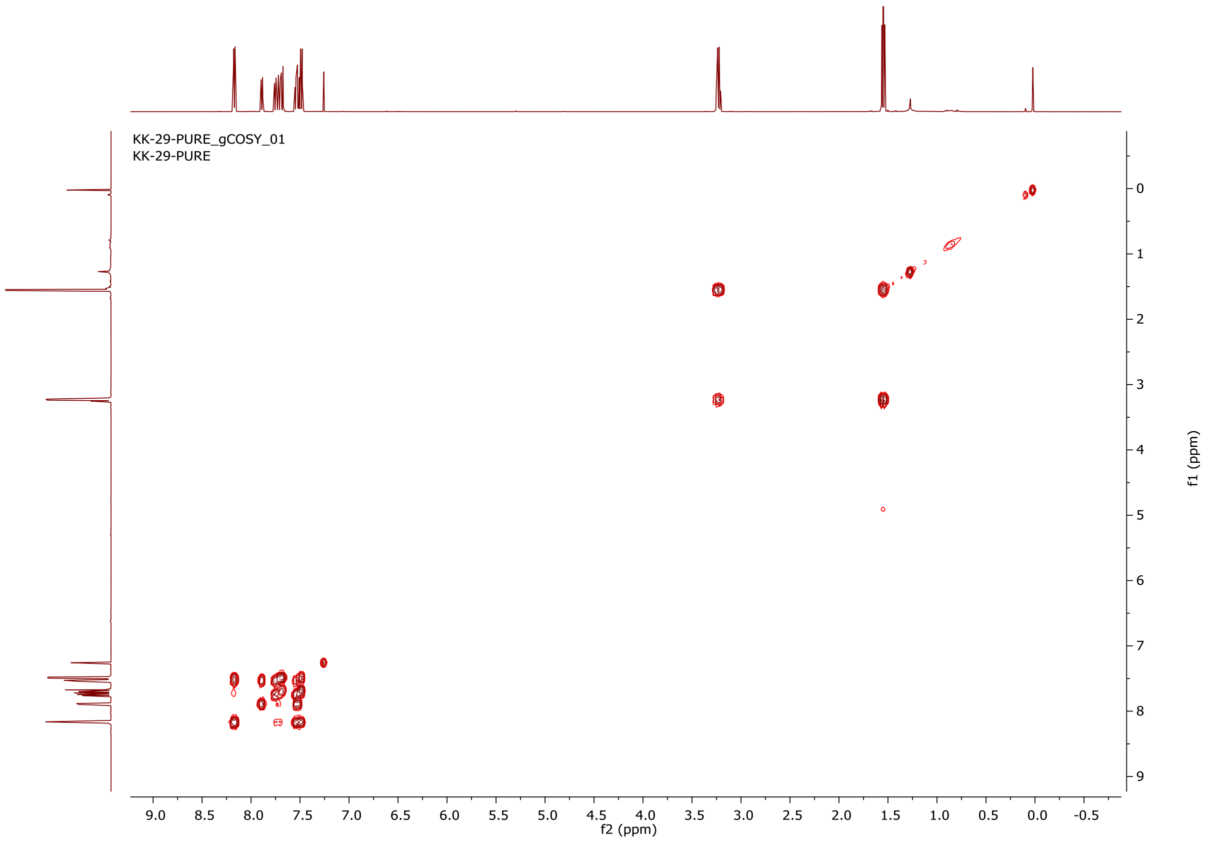

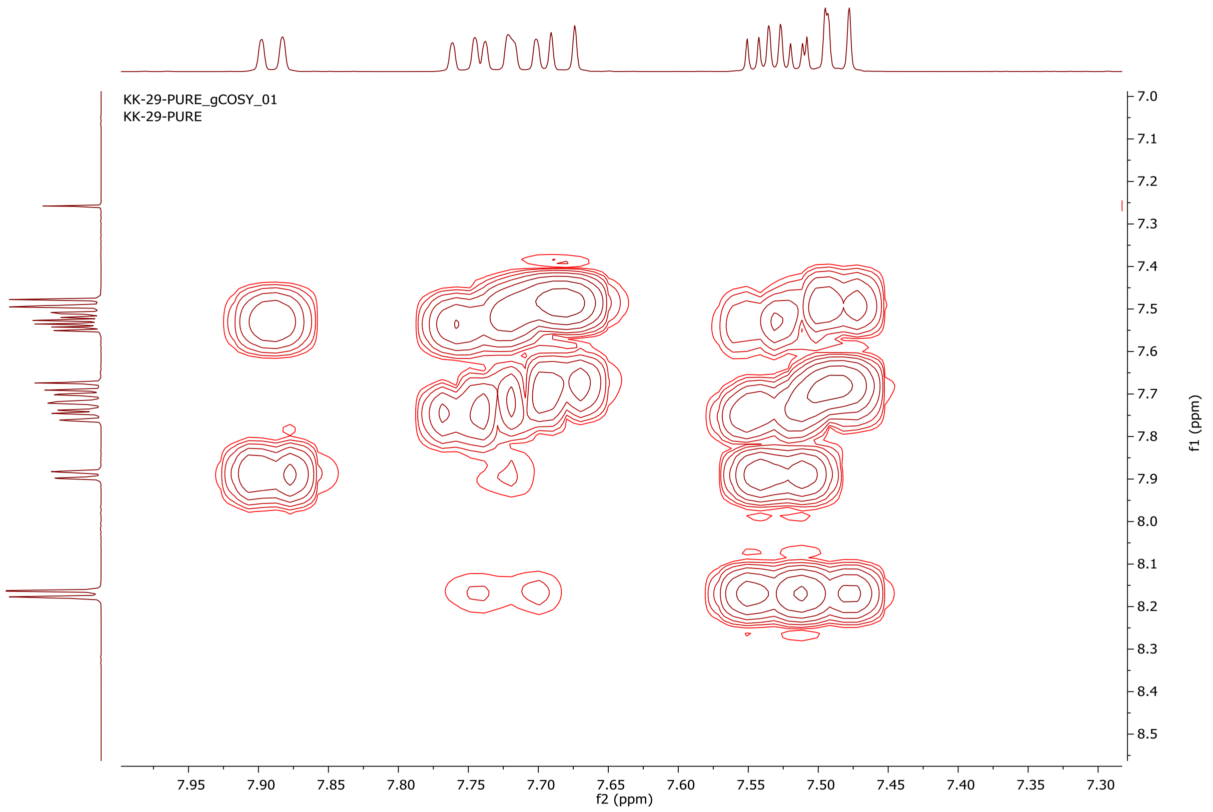

Figure S12. HSQC 2D NMR spectra of **2b** in $CDCl_3$.

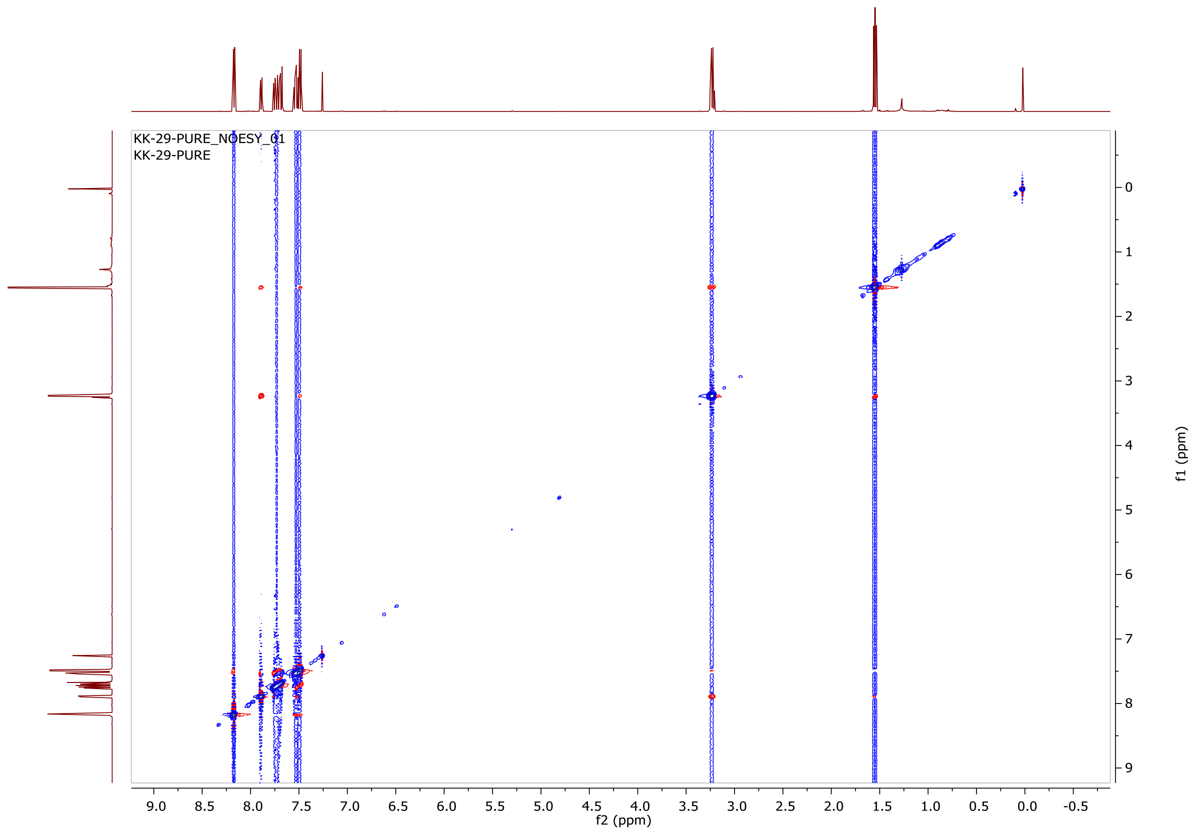

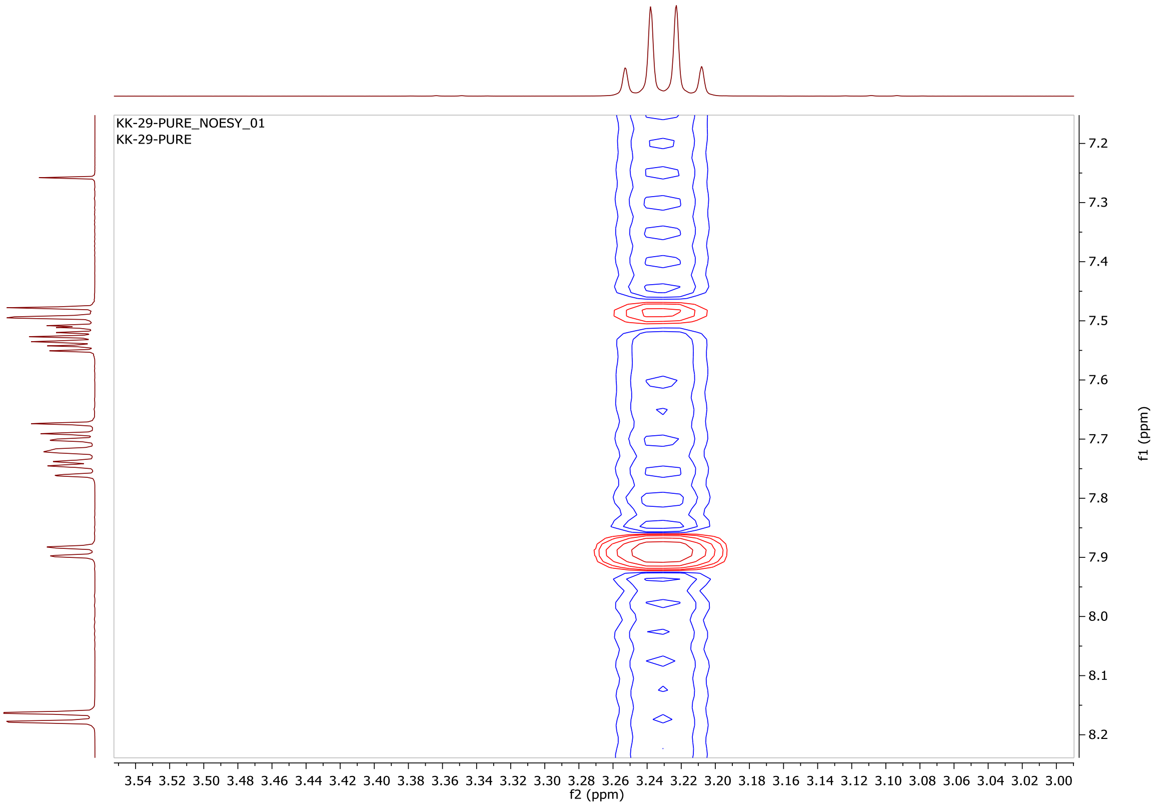

Figure S13. NOSEY 2D NMR spectra of **2b** in $CDCl_3$.

1-*iso*-Butylperylene (2c)

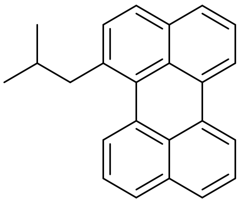

2c

According to the general procedure (I),

Perylene (**1a**) (1.0 mmol, 252 mg), *iso*-BuLi (1.7 M in heptane, 1.2 mmol, 0.70 mL) were reacted in THF (40 mL) under nitrogen at -30 °C for 2.5 h. Aqueous work up with ammonium chloride solution followed by column chromatography using cyclohexane:toluene (9:1) as an eluent afforded 1-*iso*-butylperylene (**2c**) as a yellow powder. mp (DSC): 114 °C (onset), yield (105 mg, 34%). R_f = 0.48 in cyclohexane:toluene (9:1).

According to the general procedure (II), Perylene (**1a**) (1.0 mmol, 252 mg), *iso*-BuLi (1.7 M in heptane, 1.2 mmol, 0.70 mL) were reacted in THF (40 mL) under nitrogen at -30 °C for 2.5 h. Reaction was quenched with I_2 (3.0 equiv.) followed by work up and column chromatography using cyclohexane:toluene (9:1) as an eluent to afford 1-*iso*-butylperylene (**2c**) as a yellow solid. Yield (196 mg, 64%).

^{1}H NMR (400 MHz, $CDCl_3$): 0.95 (d, 6H, J = 6.8 Hz, -CH(C**H**$_3$)$_2$, *i*-Bu), 2.21 (tp, 1H, J_1 = 13.4 Hz, J_2 = 6.8 Hz, -C**H**(CH$_3$)$_2$, *i*-Bu), 3.19 (d, 2H, J = 7.0 Hz, -C**H**$_2$CH(CH$_3$)$_2$, *i*-Bu), 7.56-7.47 (m, 4H), 7.66 (d, 1H, J = 8.4 Hz), 7.77-7.70 (m, 3H), 7.96 (dd, 1H, J_1 = 7.6 Hz, J_2 = 1.0 Hz,), 8.17 (ddd, 2H, J_1 = 7.6 Hz, J_2 = 2.4 Hz, J_3 = 1.2 Hz).**^{13}C NMR (100 MHz, $CDCl_3$):** δ = 22.7, 28.8, 45.2, 119.9, 120.8, 125.6, 125.7, 126.2, 126.8, 126.97, 126.98, 127.3, 130.0, 130.90, 130.91, 131.2, 131.5, 132.7, 134.1, 137.3. **IR (ATR cm^{-1}): ν =** 3051, 2953, 1591, 1464, 1364, 834, 813, 768. **GC-MS** (retention time = 11.859 min) m/z: 308.1 (M^+), 265.1 (M^+ - CH(CH$_3$)$_2$). **HRMS (APCI):** *m/z* calcd for $(C_{24}H_{20}+H)^+$: 309.1645; found: 309.1638.

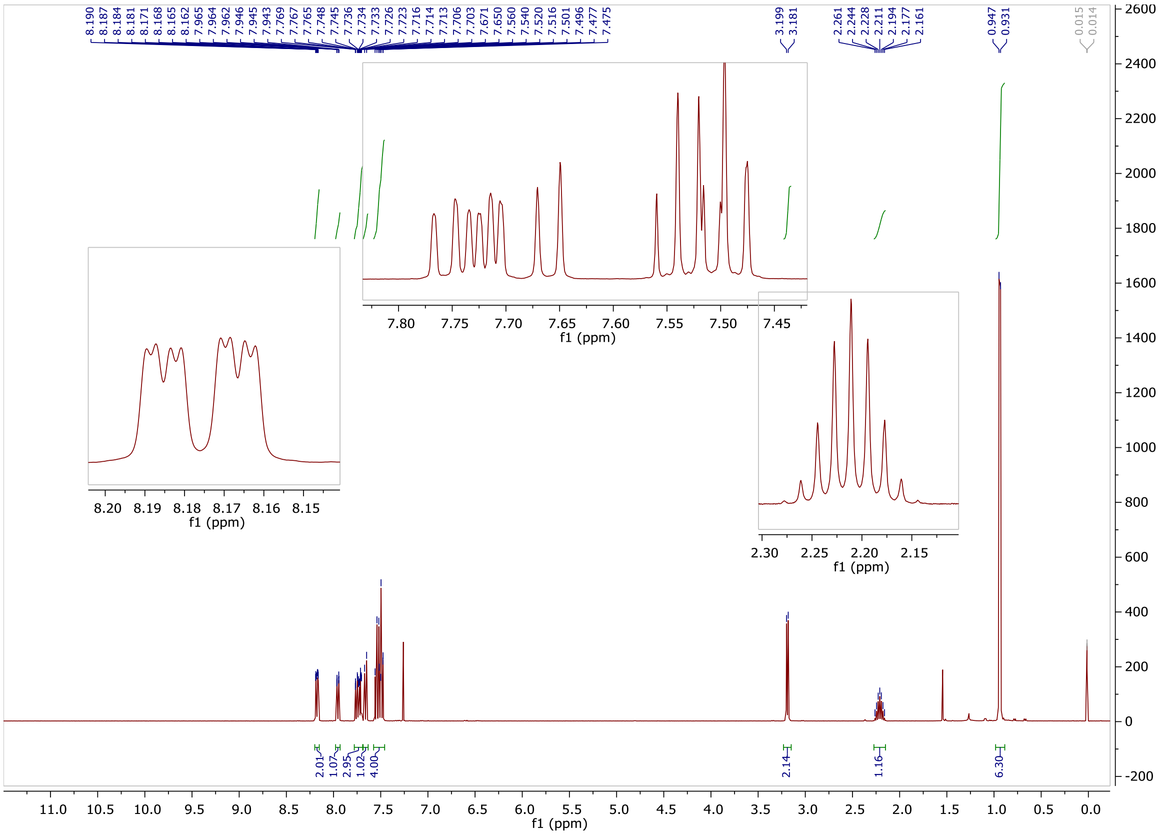

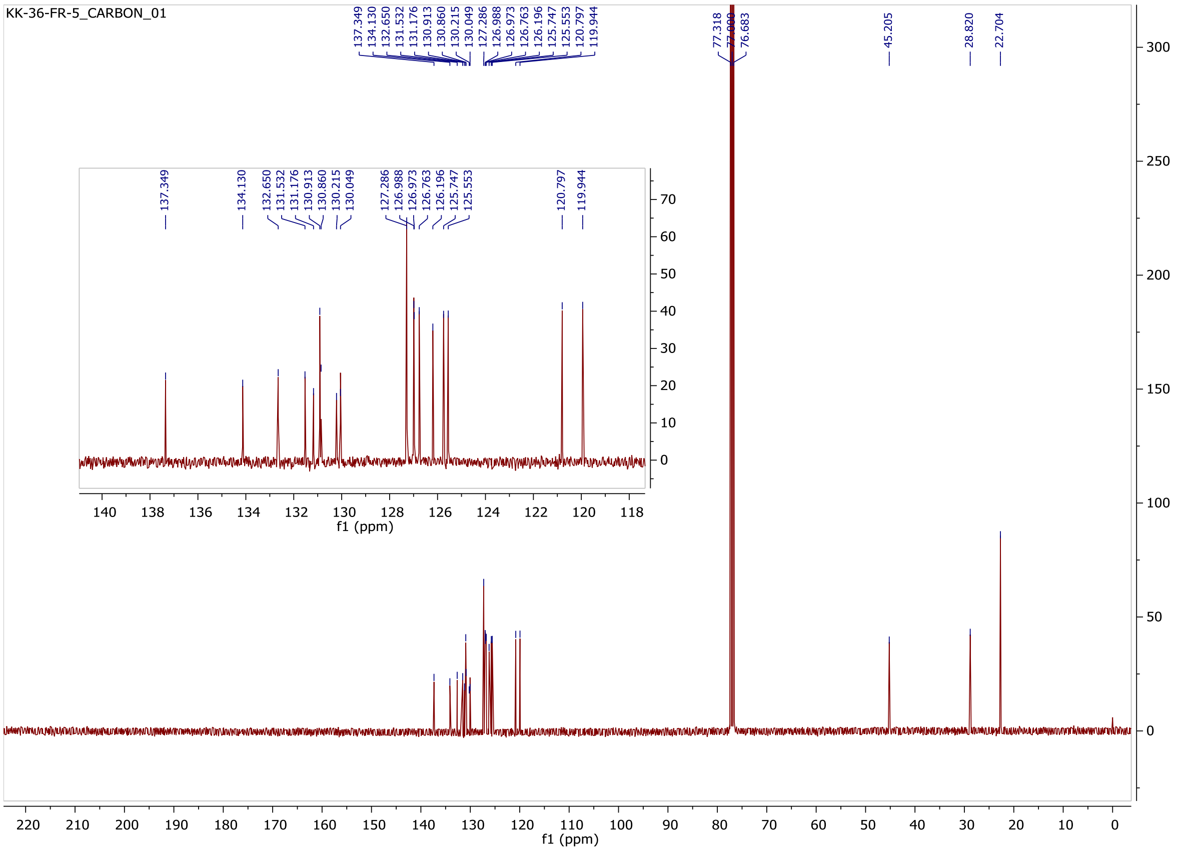

Figure S14. ^{1}H (400 MHz) and ^{13}C (100 MHz) NMR spectra of **2c** in $CDCl_3$.

1-*n*-Hexylperylene (2d)

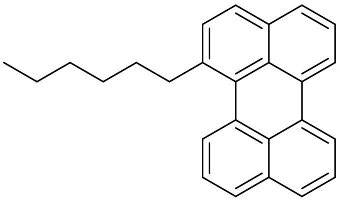

2d

According to the general procedure I, Perylene (**1a**) (1.0 mmol, 252 mg), *n*-Hexyllithium (2.3 M in Hexane, 1.5 mmol, 0.65 mL) were reacted in THF (40 mL) under nitrogen at -30 °C for 2.5 h. Aqueous work up with ammonium chloride solution followed by column chromatography using cyclohexane:toluene (9:1) as an eluent afforded 1-*n*-hexylperylene (**2d**) as a yellow-orange crystalline solid. mp (DSC): 66 °C (onset), yield (111 mg, 33%). R_f = 0.57 in cyclohexane:toluene (9:1).

According to the general procedure II, Perylene (**1a**) (1.0 mmol, 252 mg), *n*-Hexyllithium (2.3 M in Hexane, 1.5 mmol, 0.65 mL) were reacted in THF (40 mL) under nitrogen at -30 °C for 2.5 h. Reaction was quenched with I_2 (3.0 equiv.) followed by work up and column

chromatography cyclohexane:toluene (9:1) as an eluent to afford 1-*n*-hexylperylene (**2d**) as an yellow-orange crystalline solid. Yield (207 mg, 62%).

^{1}H NMR (500 MHz, CDCl$_3$): δ = 0.93 (t, 3H, J = 7.0 Hz, C**H$_3$**, *n*-Hex), 1.44-1.33 (m, 4H, 2 × CH$_2$, *n*-Hex), 1.55-1.49 (m, 2H, overlap with H_2O peak, *n*-Hex), 1.96-1.89 (m, 2H, Ar-CH$_2$C**H$_2$**), 3.18-3.15 (m, 2H, Ar-C**H$_2$**), 7.66 (d, 1H, J = 8.5 Hz), 7.73-7.69 (m, 2H), 7.75 (dd, 1H, J_1 = 8.0 Hz, J_2 = 1.0 Hz), 7.86 (dd, 1H, J_1 = 7.5 Hz, J_2 = 1.0 Hz), 8.18 (dd, 2H, J_1 = 7.0 Hz, J_2 = 1.0 Hz). **^{13}C NMR (126 MHz, CDCl$_3$):** δ = 14.1, 22.7, 29.6, 31.0, 31.7, 36.1, 120.0, 120.9, 125.6, 125.9, 126.2, 126.4, 127.03, 127.01, 127.26, 127.3, 129.5, 130.0, 130.7, 130.9, 131.2, 131.4, 132.6, 134.2, 138.5. **IR (ATR cm^{-1}):** ν = 3052, 2955, 2928, 2855, 822, 769. **GC-MS** (retention time = 13.175 min) m/z: 336.2 (M^+), 265.1 (M^+ - CH(CH$_3$)$_2$). **HRMS (APCI):** *m*/*z* calcd for $(C_{26}H_{24}+H)^+$: 337.1951; found: 337.1962.

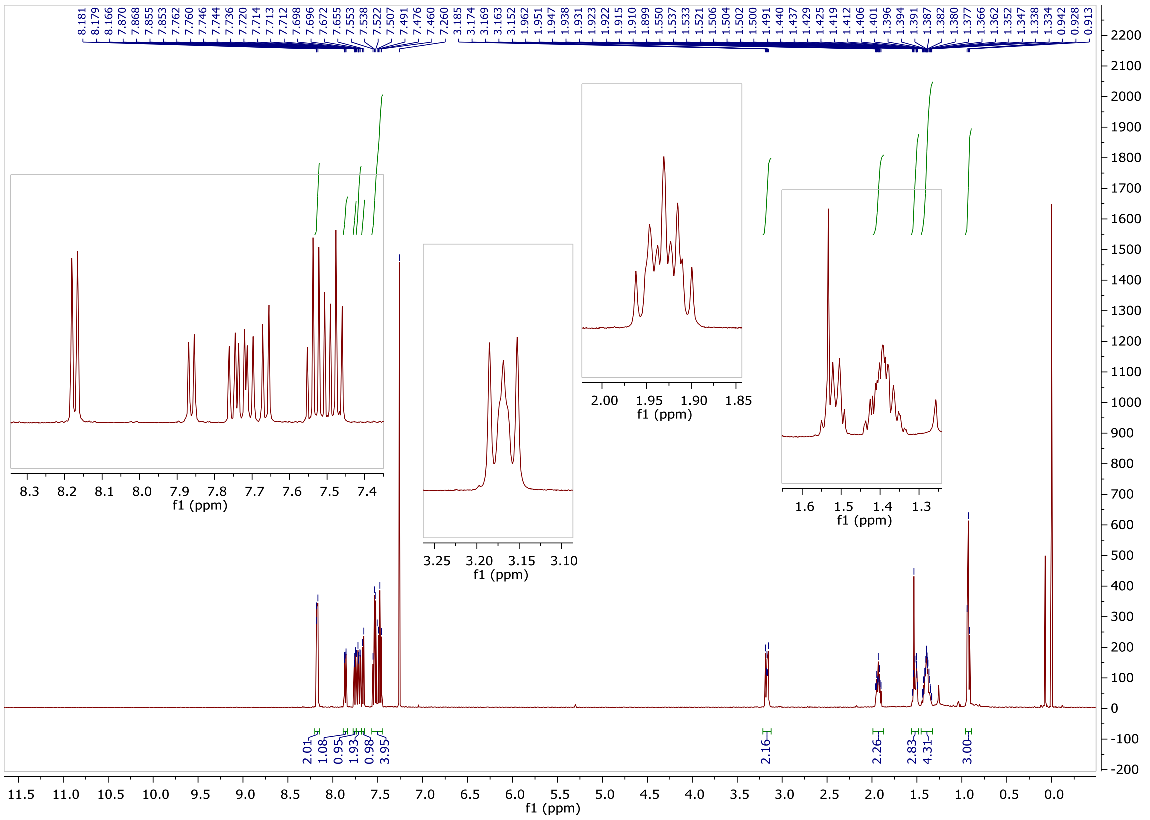

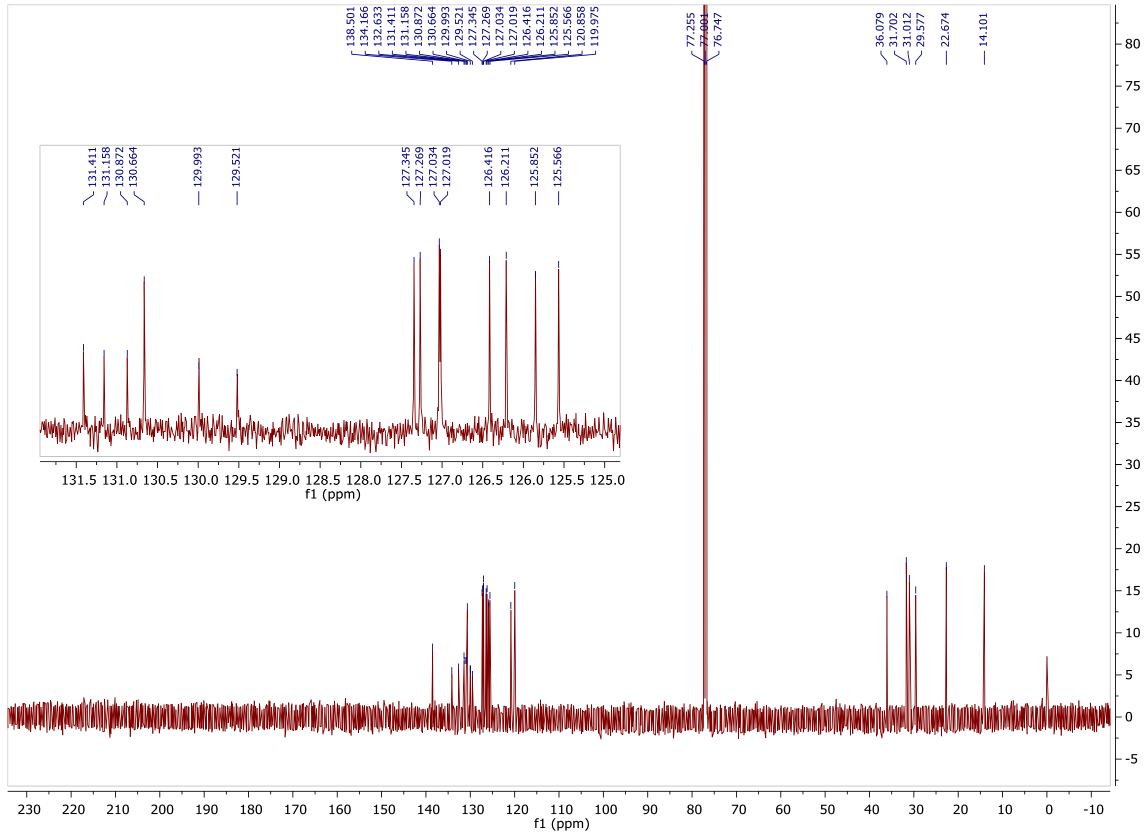

Figure S15. ^{1}H (500 MHz) and ^{13}C (126 MHz) NMR spectra of **2d** in $CDCl_3$.

1-*n*-(2-EthylHexyl)perylene (2e)

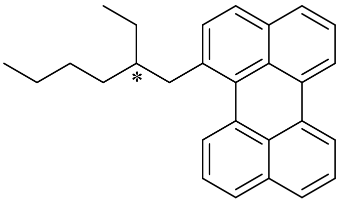

2e

According to the general procedure II, Perylene (**1a**) (1.0 mmol, 252 mg), *n*-(2-ethylhexyl)lithium (30 weight percent in heptane, 1.5 mmol, 0.82 mL) were reacted in THF (40 mL) under nitrogen at -30 °C for 2.5 h. Reaction was quenched with I_2 (3.0 equiv.) followed by work up and column chromatography cyclohexane:toluene (9:1) as an eluent to afford 1-*n*-(2-ethylhexyl)perylene (**2e**) as a crystalline yellow solid. mp (DSC): 50 °C (onset), yield (189 mg, 52%). R_f = 0.58 in cyclohexane:toluene (9:1).

^{1}H NMR (400 MHz, $CDCl_3$): 0.85-0.79 (m, 6H), 1.31-1.19 (m, 8H), 1.93-1.88 (m, 1H), 3.29 (d, 2H, J = 7.2 Hz), 7.56-7.48 (m, 4H), 7.66 (d, 1H, J = 8.4 Hz), 7.77-7.71 (m, 3H), 8.01 (dd, 1H, J_1 = 7.5 Hz, J_2 = 1.0 Hz), 8.17 (ddd, 2H, J_1 = 7.6 Hz, J_2 = 1.2 Hz). **^{13}C NMR (100 MHz, $CDCl_3$):** δ = 10.6, 14.1, 23.0, 25.7, 28.7, 32.7, 39.5, 40.2, 119.9, 120.8, 125.5, 125.7, 126.2,

126.6, 126.9, 127.1, 127.2, 130.0, 130.03, 130.4, 130.8, 130.9, 131.2, 131.5, 132.5, 134.1, 137.6. **IR (ATR cm^{-1}):** ν = 2955, 2923, 2855, 1725, 1587, 1456, 1378, 1262, 1015, 804, 754, 735, 540. **GC-MS** (retention time = 12.753 min) m/z: 364.2 (M^+), 265.1 (M^+ - C_7H_{15}). **HRMS (APCI):** *m*/*z* calcd for ($C_{24}H_{20}$+H)$^+$: 365.2264; found: 365.2265.

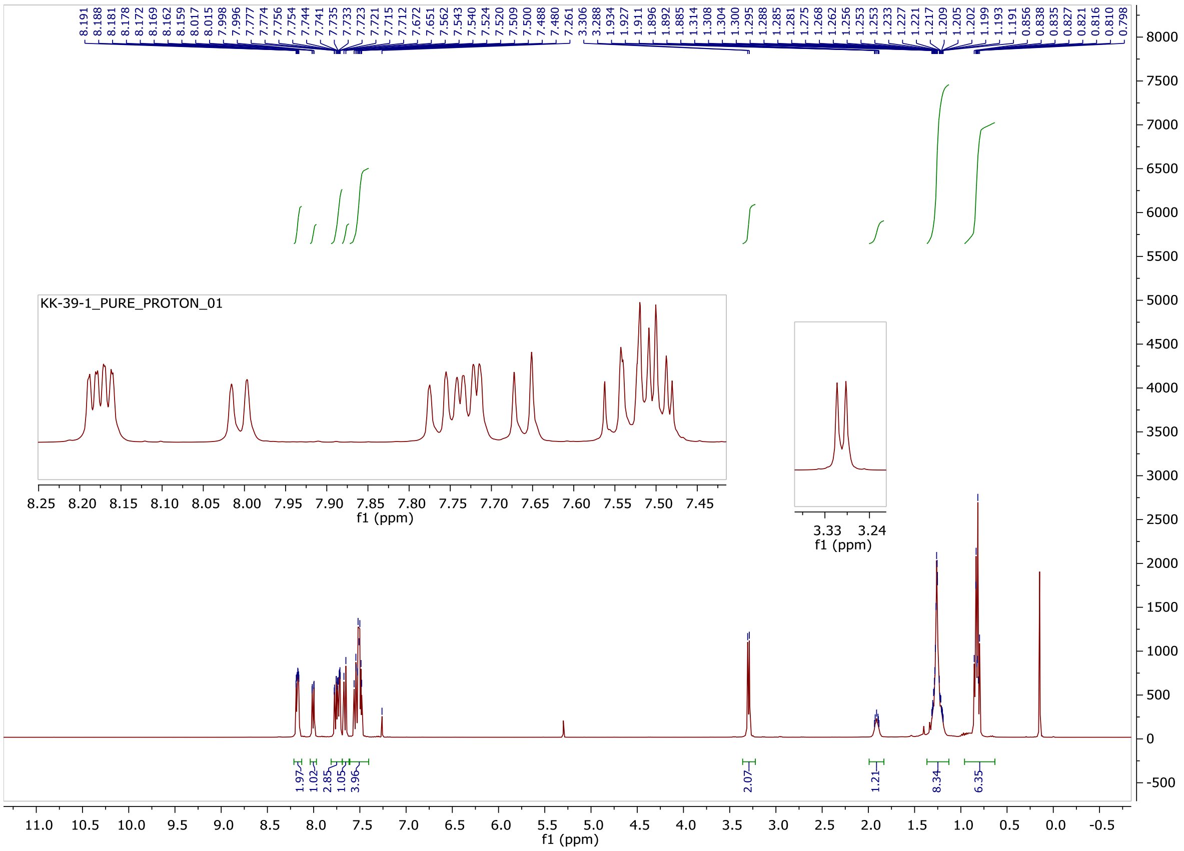

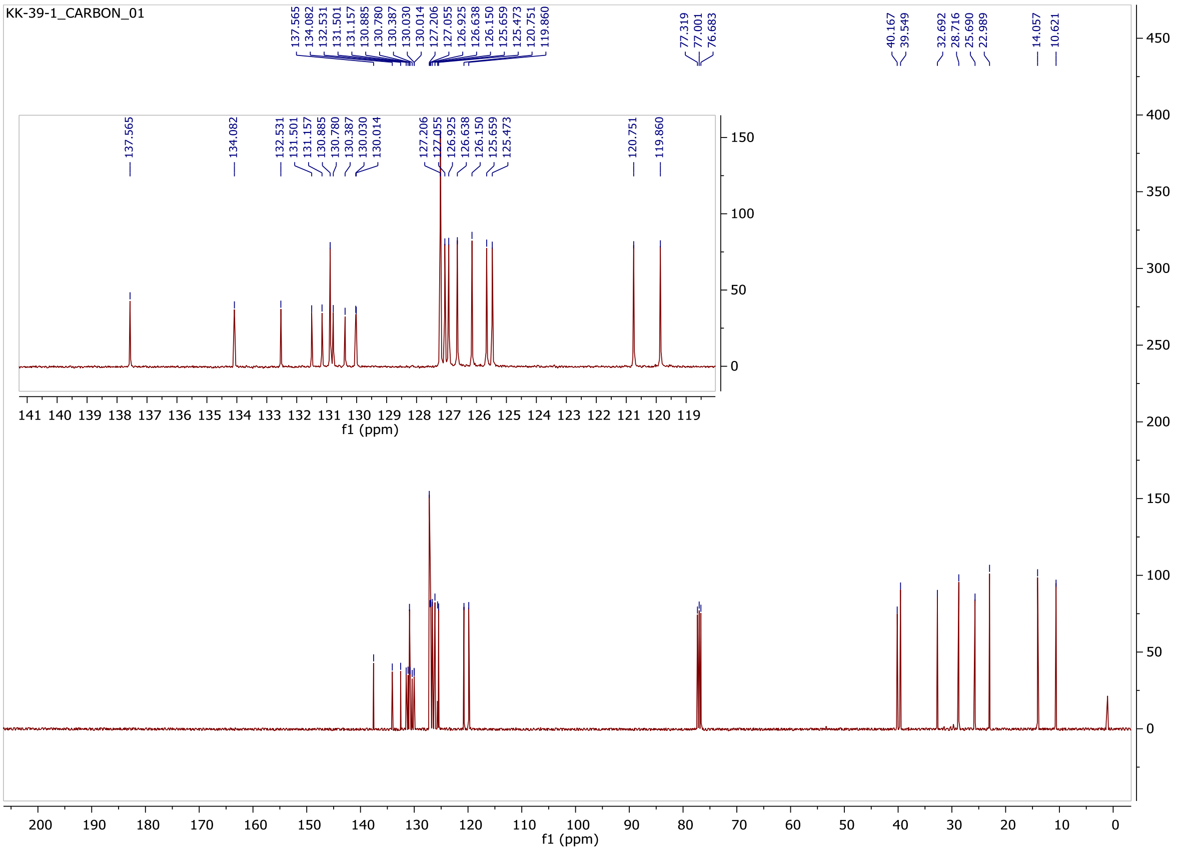

Figure S16. ^{1}H (400 MHz) and ^{13}C (100 MHz) NMR spectra of **2e** in $CDCl_3$.

1-*sec*-Butylperylene (2f)

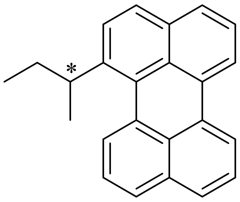

2f

According to the general procedure (I), Perylene (**1a**) (0.5 mmol, 126 mg), *s*-BuLi (1.4 M in cyclohexane, 0.60 mmol, 0.42 mL) were reacted in dry THF (20 mL) under nitrogen at -30 °C for 2.5 h. Aqueous work up with ammonium chloride solution followed by column chromatography using cyclohexane:toluene (9:1) as an eluent afforded 1-*sec*-butylperylene (**2f**) as a yellow solid. mp (DSC): 116 °C (onset), yield (72 mg, 47%). R_f = 0.55 in cyclohexane:toluene (9:1).

^{1}H NMR (500 MHz, $CDCl_3$): 1.52 (d, 3H, J = 6.5 Hz), 1.73 (ddq, 2H, J_1 = 14.5 Hz, J_2 = 6.5 Hz, J_3 = 3.0 Hz, C$\mathbf{H_aH_b}$CH), 3.73 (dq, 1H, CH_aH_bC**H**CH_3, J_1 = 14.0 Hz, J_2 = 7.0 Hz), 7.56-7.48 (m, 4H), 7.72-7.69 (m, 2H), 7.73 (d, 1H, J = 8.0 Hz), 7.76 (d, 2H, J = 8.0 Hz), 8.16 (ddd, 2H, J_1 = 8.5 Hz, J_2 = 7.5 Hz, J_3 = 1.0 Hz).**^{13}C NMR (126 MHz, $CDCl_3$):** δ = 12.2, 22.0, 32.0,

37.3, 120.0, 120.9, 125.6, 125.8, 126.2, 126.5, 126.92, 126.94, 127.1, 127.3, 129.4, 129.7, 130.2, 130.8, 131.1, 131.2, 132.2, 134.1, 143.7. **IR (ATR cm^{-1}):** **ν =** 2957, 2923, 2870, 810, 764. **GC-MS** (retention time = 11.926 min) m/z: 308 (M^+), 279.1 (M^+ - CH_2CH_3). **HRMS (APCI):** *m*/*z* calcd for $(C_{24}H_{20}+H)^+$: 309.1645; found: 309.1638.

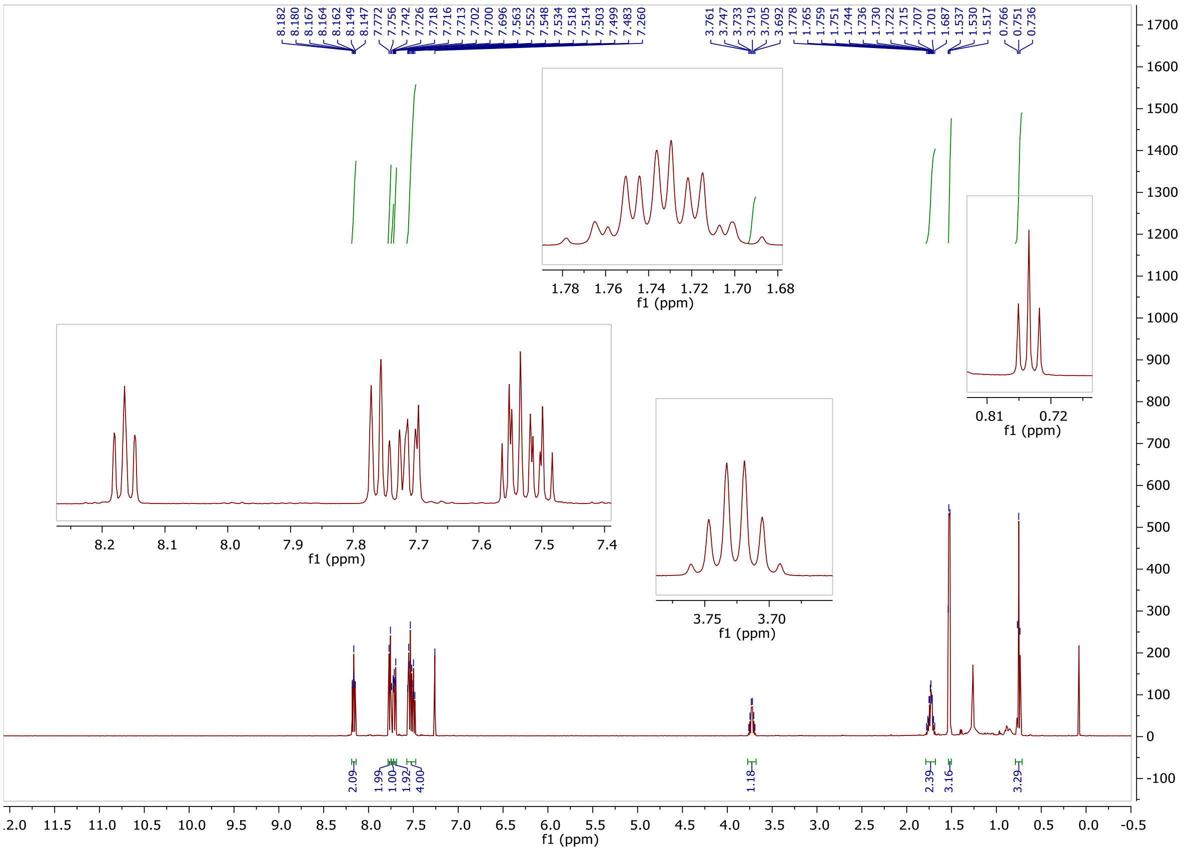

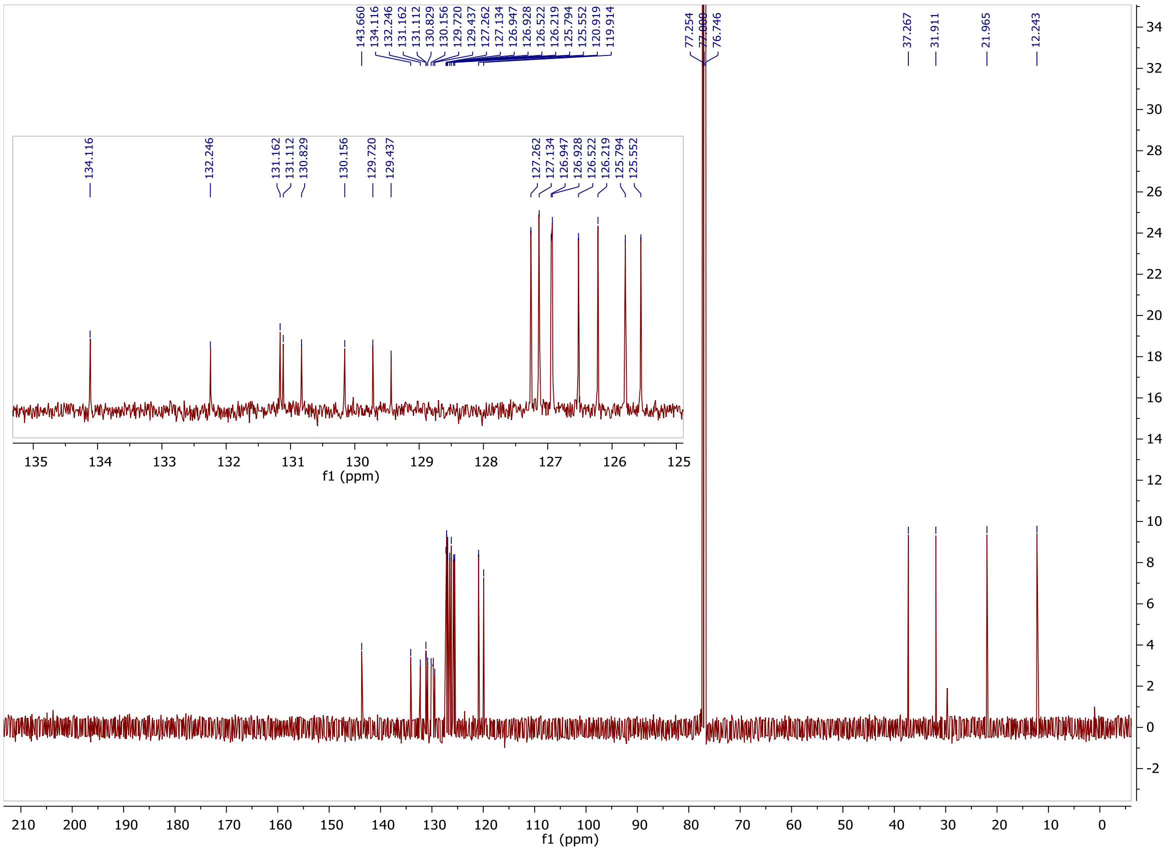

Figure S17. ^{1}H (500 MHz) and ^{13}C (126 MHz) NMR spectra of **2f** in $CDCl_3$.

3-*sec*-Butylperylene (2g)

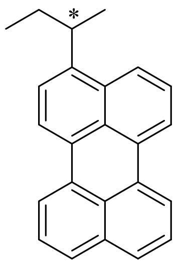

2g

Elutions using cyclohexane:toluene (85:15) afforded 3-*sec*-butylperylene (**2g**) as a yellow solid. mp (DSC): 131 °C (Peak), yield (20 mg, 13%). R_f = 0.48 in cyclohexane:toluene (9:1).

^{1}H NMR (500 MHz, $CDCl_3$): δ = 0.96 (t, 3H, J = 7.5 Hz, $CH_aH_bC\mathbf{H_3}$, *s*-Bu), 1.39 (d, 3H, $C\mathbf{H_3}CH$, *s*-Bu), 1.75 (dq, 1H, J_1 = 14.5 Hz, J = 7.0 Hz, $C\mathbf{H_a}H_bCH_3$), 1.87 (dq, 1H, J_1 = 14.0 Hz, J = 7.5 Hz, $CH_a\mathbf{H_b}CH_3$), 3.45 (dq, 1H, $CH_3C\mathbf{H}CH_aH_b$, J_1 = 14.0 Hz, J = 7.0 Hz,), 7.41 (d, 1H, J = 8.0 Hz), 7.47 (td, 2H, J_1 = 8.0 Hz, J_2 = 1.5 Hz), 7.51 (dd, 1H, J_1 = 8.5 Hz, J_2 = 7.5 Hz), 7.65 (t, 2H, J = 8.5 Hz), 7.98 (d, 1H, J = 8.5 Hz), 8.16 (dd, 1H, J_1 = 7.5 Hz, J_2 = 1.0 Hz), 8.18 (dd, 2H, J_1 = 7.5 Hz, J_2 = 1.5 Hz), 8.21 (dd, 1H, J_1 = 7.5 Hz, J_2 = 1.0 Hz). **^{13}C NMR (126 MHz, $CDCl_3$):** δ = 12.2, 21.0, 30.4, 35.5, 119.6, 120.0, 120.1, 120.3, 123.2, 123.3, 126.2, 126.5, 126.6,

127.2, 127.6, 128.5, 129.0, 129.1, 131.5, 131.6, 131.8, 133.0, 134.6, 143.7. **IR (ATR cm^{-1}): ν** = 3053, 2961, 2925, 822, 770. **GC-MS** (retention time = 12.93 min) m/z: 308 (M^+), 279.1 (M^+ -C_2H_5). **HRMS (APCI):** *m/z* calcd for $(C_{24}H_{20}+H)^+$: 309.1638; found: 309.1643.

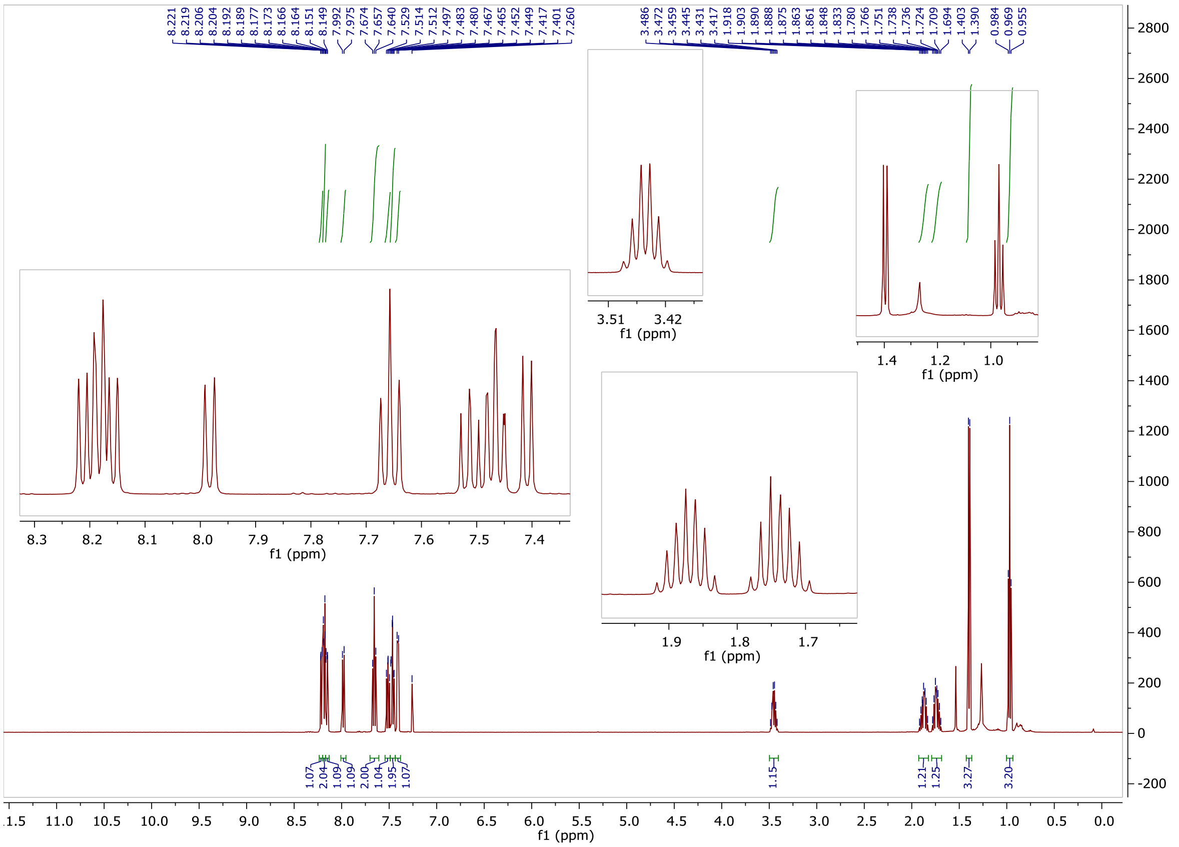

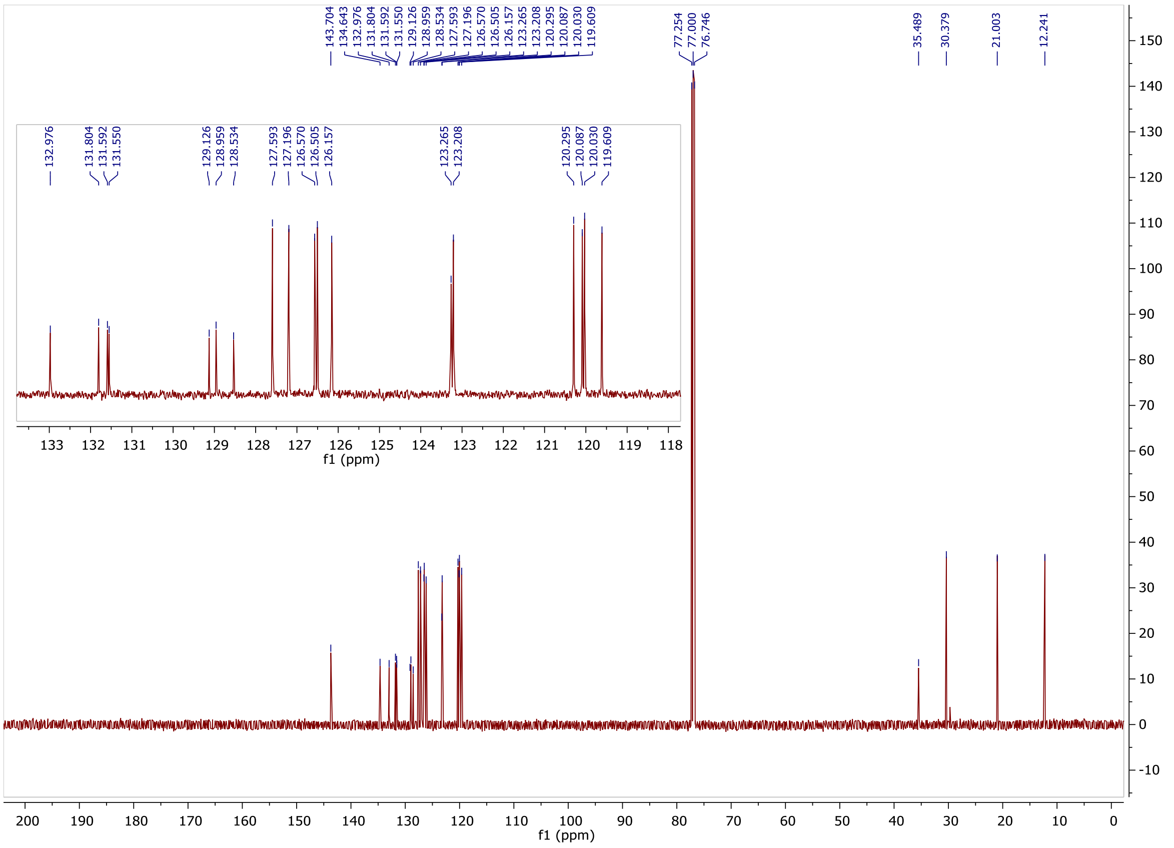

Figure S18. ^{1}H (500 MHz) and ^{13}C (126 MHz) NMR spectra of **2g** in $CDCl_3$.

3-*tert*-butylperylene (2h)

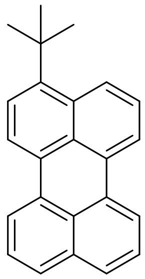

2h

According to the general procedure (I), Perylene (**1a**) (0.5 mmol, 126 mg), 3-*tert*-BuLi (1.9 M in pentane, 0.65 mmol, 0.32 mL) were reacted in THF (20 mL) under nitrogen at -30 °C for 2.5 h. Aqueous work up with ammonium chloride solution followed by column chromatography using cyclohexane:toluene (88:12) as an eluent afforded 3-*tert*-butylperylene (**2h**) as a yellow solid. mp (DSC): 217 °C (onset); yield (14 mg, 9%). R_f = 0.46 in cyclohexane:toluene (9:1). **^{1}H NMR (400 MHz, $CDCl_3$):** δ = 1.65 (s, 9H, 3 × C**H_3**, *t*-Bu), 7.52-7.45 (m, 3H), 7.67 (dd, 1H, J_1= 7.6 Hz, J_2 = 0.8 Hz), 8.18 (ddd, 2H, J_1 = 8.4 Hz, J_2 = 7.5 Hz, J_2 = 1.1 Hz), 8.22 (dd, 1H, J_1 = 7.6 Hz, J_2 = 0.8 Hz), 8.33 (dd, 1H, J_1 = 8.8 Hz, J_2 = 1.2 Hz). **^{13}C NMR (100 MHz,**

CDCl3): δ = 31.8 (C(**<u>C</u>**H3)3), 36.0 (**<u>C</u>**(CH3)3), 119.7, 119.8, 120.07, 120.09, 124.1, 125.0, 126.57, 126.59, 126.9, 127.3, 127.5, 128.3, 129.6, 130.1, 131.4, 131.7, 132.8, 134.5, 146.0. **IR (ATR cm^{-1}):** ν = 2956, 2921, 808, 766. **GC-MS** (retention time = 13.01 min) m/z: 308 (M^+), 293.1 (M^+ -CH_3). **HRMS (APCI):** *m/z* calcd for ($C_{24}H_{20}$+H)$^+$: 309.1638; found: 309.1641.

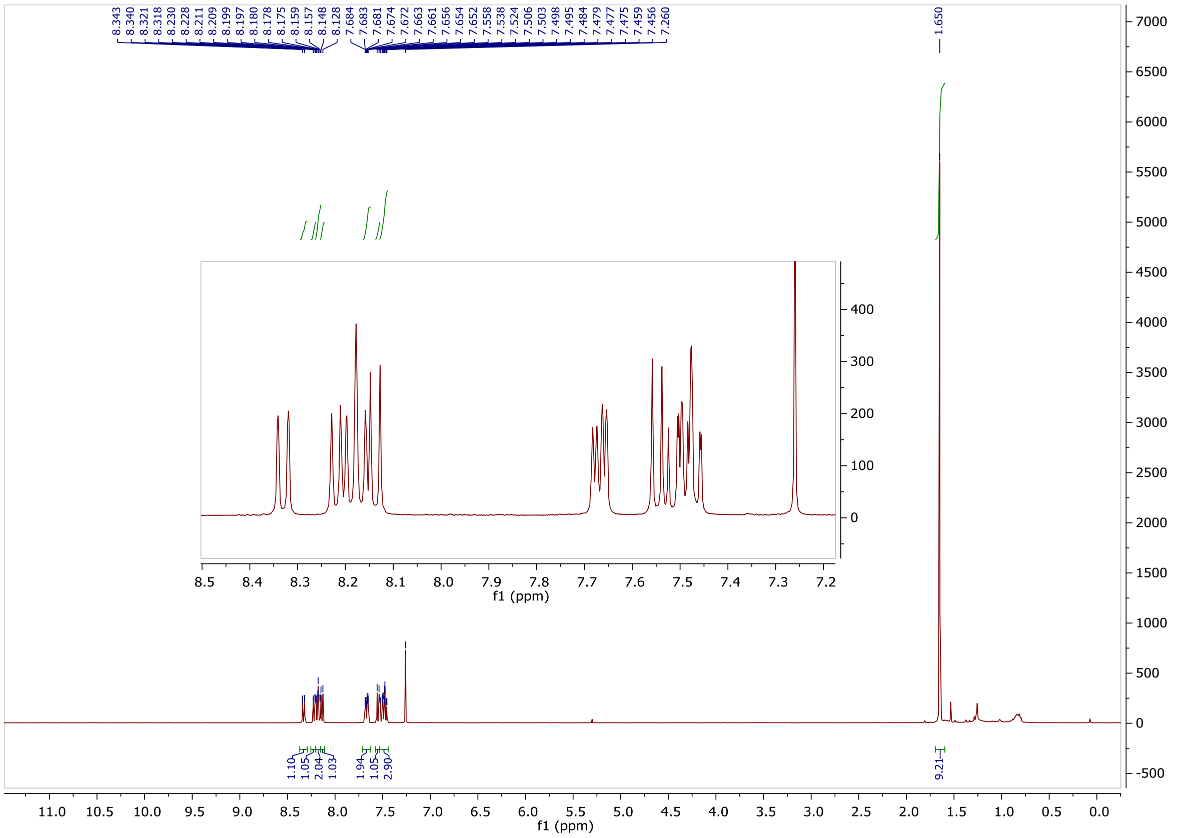

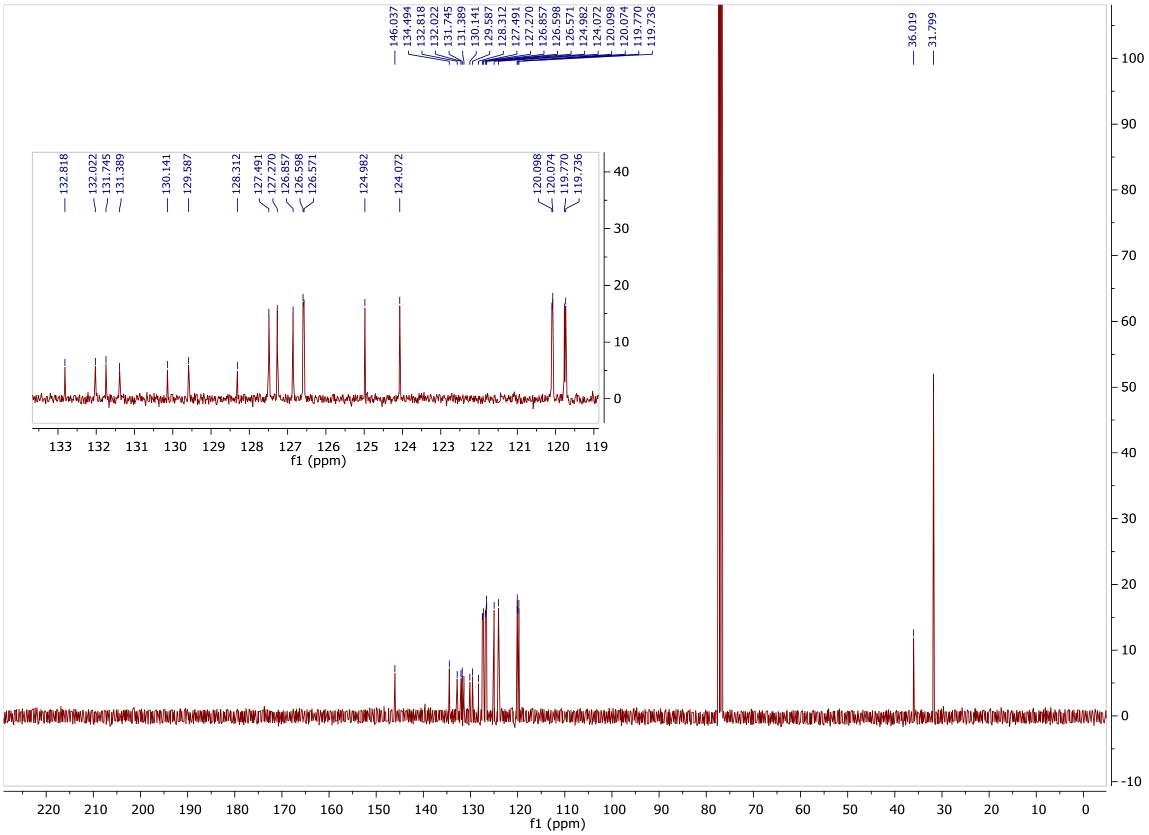

Figure S19. ^{1}H (400 MHz) and ^{13}C (100 MHz) NMR spectra of **2h** in $CDCl_3$.

4. Synthesis and characterization of compounds 2i-j

Perylene (1.26 g, 5.0 mmol), $AlCl_3$ (0.200 g, 1.5 mmol) and *tert*-butylchloride (1.85 g, 20 mmol) was reacted in dry chlorobenzene (200 mL) at rt for 72 h according to a literature procedure. Following the work-up as reported and column chromatography on alumina and elutions with cyclohexane yielded 2,5,8,11-tetra-*tert*-butylperylene (**2j**) in (0.030 g) 12.6 % yield. Further, elutions with cyclohexane gave mixtures of trisubstituted perylenes as indicated by GC-MS. Further, mixtures of disubstituted *tert*-butylperylenes and 2-*tert*-butylperylene (**2j**) were obtained in cyclohexane:toluene (90:10). Only very small amounts of **2j** (0.034, 2.3%) could be isolated and pure 2,5-di-*tert*-butylperylene (traces) could be obtained.

2,5,8,11-tetra-*tert*-Butylperylene (2i)

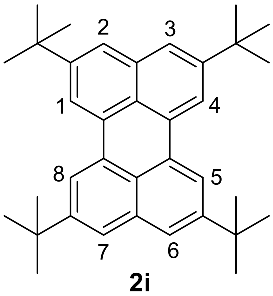

2i

Large amounts of **2i** was synthesized using a literature procedure[2] according to which a 20 mL microwave vial was charged with Perylene (**1a**) (3.9 mmol, 1.0 g), ferric chloride (5.15 mmol, 835 mg) under nitrogen. Chlorobenzene (7 mL) was added to the above mixture followed by addition of *t*-BuBr (43.9 mmol, 4.93 mL) and reaction was allowed to stir at 95 °C for 5h.[2] Afterwards, DCM was added (50 mL) and contents were passed through a short column of basic alumina. Organic layer was extracted with H_2O thrice, solvent was removed under vacuum. Column chromatography using basic alumina and cyclohexane as an eluent resulted in pure 2,5,8,11-tetra-*tert*-butylperylene (**2i**) as a yellow solid. mp (DSC): 321 °C (onset); Lit[2] 340 °C, Lit[3] 360 °C, yield (440 mg, 24.4%), R_f = 0.68 in cyclohexane:toluene (9:1).

^{1}H NMR (500 MHz, $CDCl_3$): δ = 7.62 (d, 4H, J_m = 2.0 Hz, H-3, H-4, H-9, H-10), 8.24 (d, 4H, J_m = 2.0 Hz, H-1, H-6, H-7, H-12). **^{13}C NMR (126 MHz, $CDCl_3$):** δ = 31.3, 34.9, 117.7, 123.2, 125.7, 130.7, 134.9, 148.7. **IR (ATR cm^{-1}):** ν = 2961, 1613, 1363, 876, 635. **GC-MS** (retention time = 16.899 min) m/z: 476.4 (M^+). **HRMS (APCI):** *m*/*z* calcd for $(C_{24}H_{20}+H)^+$: 476.3516; found: 477.3520.

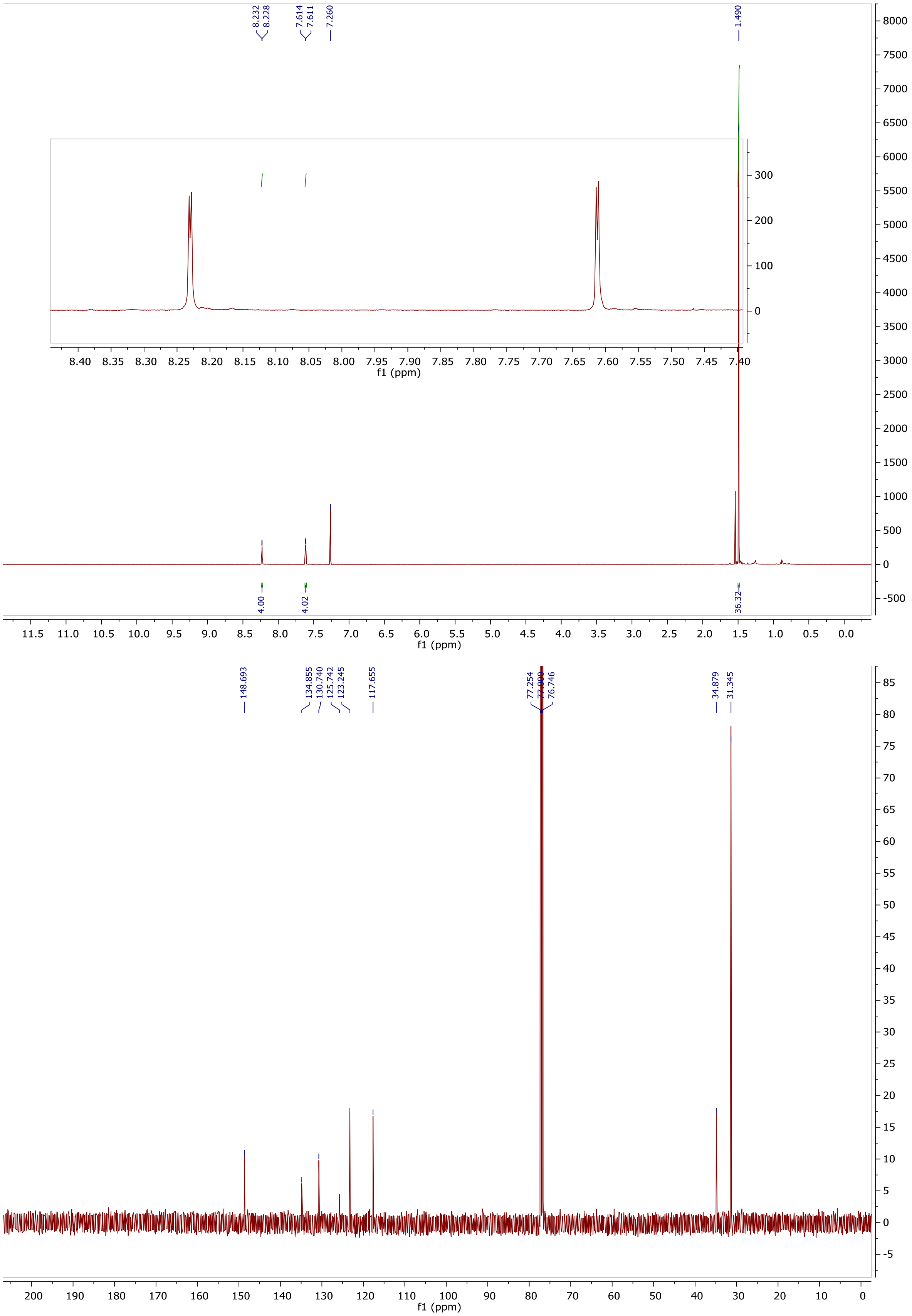

Figure S20. ^{1}H (500 MHz) and ^{13}C (126 MHz) NMR spectra of **2i** in $CDCl_3$.

2-*tert*-Butylperylene (2j)

We tried to optimize conditions to get **2j** as the major product. In most of the cases crude ^{1}H NMR indicated large amount of starting material perylene and minor peaks for 2-*tert*-butylperylene (**2j**) with small amounts of 2,5-di-tert-butylperylene (**2j'**). However reaction under dilute conditions and with excess of *tert*-butylchloride resulted in much better yields for **2j**.[2, 3]

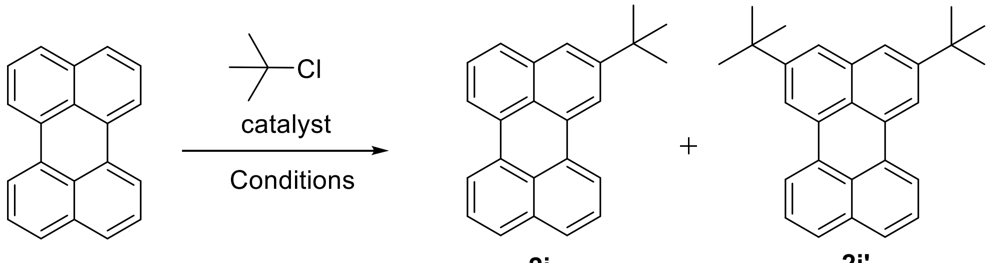

2j **2j'**

Table S2: Reaction of Perylene with *t*-butylchloride under different conditions.

S. no.	Perylene (amount)	*t*-BuCl (equiv.)	Catalyst/ Equiv.	Solvent	T (°C)/ Time (h)	Crude ^{1}H NMR
1	63 mg	1.0	$AlCl_3$/2.0	CH_3NO_2 (5 mL)	rt to 40/ 16	SM only
2	63 mg	4.2	$AlCl_3$/4.5	CH_2Cl_2 (10 mL)	0 for 10 min to rt/ 2	SM only
3	63 mg	0.5	$AlCl_3$/4.5	CH_2Cl_2 (10 mL)	0 for 10 min to rt/ 16	SM (major) + **2j** (minor) + di-product (traces)
4	63 mg	10.0	$AlCl_3$/6.0	CH_2Cl_2 (10 mL)	rt / 16	SM (major) + **2j** (traces)
5	63 mg	10.0	$AlCl_3$/6.0	CH_2Cl_2 (10 mL)	0 for 35 min to rt/ 16	SM (major) + **2j** & **2j'** + additional signals
6	38 mg	10.0	$AlCl_3$/5.0	CH_2Cl_2 (10 mL)	0 for 2.5 h	**2j** + SM (major) **2j'** (minor)

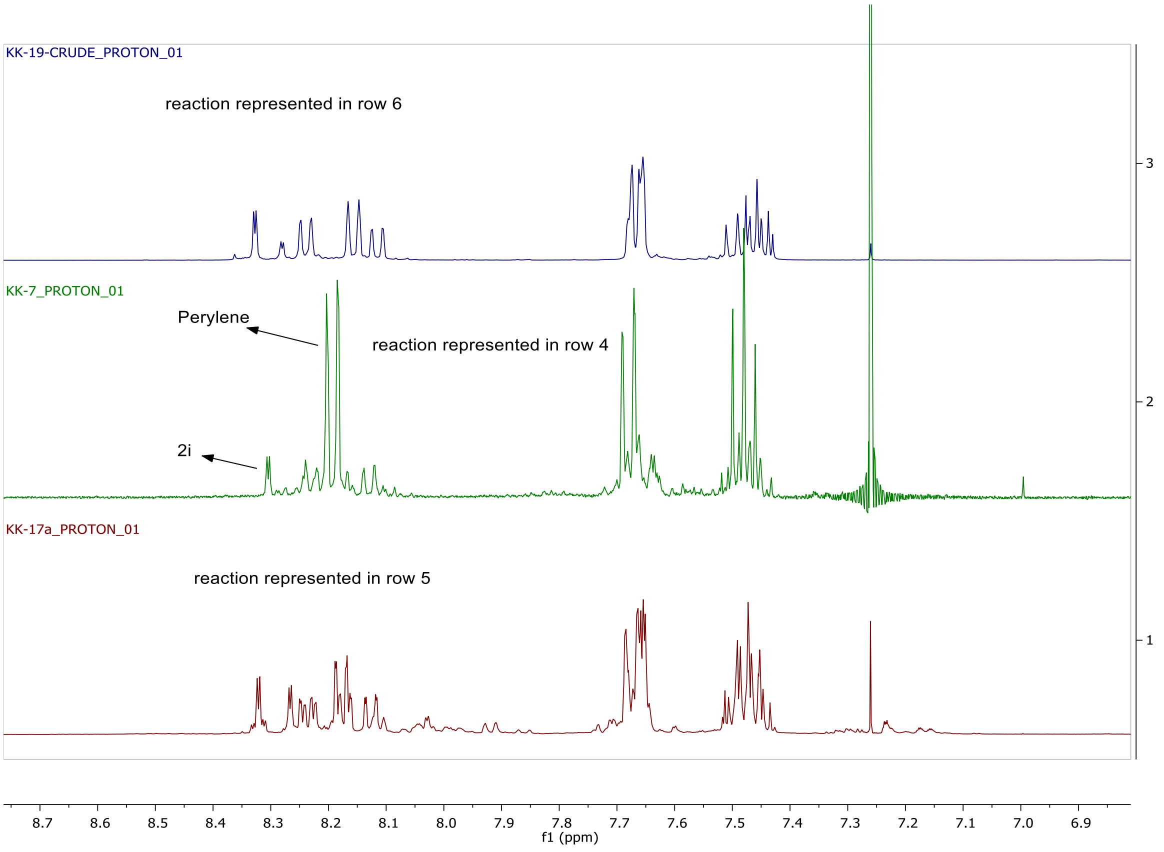

Figure S21. Crude ^{1}H NMR spectrum represented by topmost (row-6, table S2), middle (row 4, table S2) and bottom (row 5, table S2).

New procedure for synthesis of 2j

A 10 mL microwave vial was charged with Perylene (**1a**) (0.15 mmol, 38 mg), $AlCl_3$ (0.75, 97 mg) in glove box and dissolved in dry DCM (8 mL) under nitrogen. To the above mixture, *t*-butylchloride (1.5 mmol, 0.14 mL) in 2 mL of dry DCM was added slowly under nitrogen at 0 °C in 30 minutes. The reaction was maintained at the same temperature for additional 2 h. Afterwards, the reaction mixture was diluted with DCM (20 mL) and washed with $NaHCO_3$ solution twice. Combined organic layers were dried using sodium sulphate, filtered and solvent was evaporated under vacuum. Purification by preparative TLC using cyclohexane:toluene (6:1) afforded 2-*t*-butylperylene (**2j**) as yellow solid. Alternatively flash column chromatography using biotage SNAP ultra 25g (flow rate 12 mL/min, solvent system cyclohexane:toluene 90:10) also resulted in pure **2j**. mp (DSC): 146 °C (onset); Lit[3] 145-146 °C, yield (13 mg, 28%), R_f = 0.48 in cyclohexane:toluene (9:1).

^{1}H NMR (400 MHz, $CDCl_3$): δ = 1.48 (s, 9H, 3 × C**H**$_3$, *t*-Bu), 7.43-7.50 (m, 4H), 7.63-7.68 (m, 4H), 8.13 (dd, 1H, J_o = 7.6 Hz, J_m = 1.2 Hz), 8.17 (dd, 1H, J_o = 7.6 Hz, J_m = 1.2 Hz), 8.23 (dd, 1H, J_o = 7.6 Hz, J_m = 1.2 Hz), 8.31 (d, 1H, J_m = 2.0 Hz). **^{13}C NMR (100 MHz, $CDCl_3$):** δ

= 31.3, 34.9, 118.9, 119.6, 119.9, 120.1, 123.4, 126.4, 126.50, 126.52, 127.1, 127.68, 127.69, 127.9, 128.9, 130.80, 130.87, 131.3, 131.5, 134.76, 134.8, 148.9. **IR (ATR cm^{-1}): ν** = 3050, 2960, 1593, 1463, 1367, 1259, 869, 836, 812, 799, 767. **GC-MS** (retention time = 12.225 min) m/z: 308.1 (M^+), 293.1 (M^+ -CH_3). **HRMS (APCI):** *m/z* calcd for $(C_{24}H_{20}+H)^+$: 309.1638; found: 309.1638.

2,5-di-*tert*-butylperylene (**2j'**) could not be obtained in sufficient purity and its photophysical properties were not studied. **^{1}H NMR (400 MHz, $CDCl_3$):** δ = 1.47 (s, 18H, 2 × C**H**$_3$, *t*-Bu), 7.46-7.49 (m, 2H, H-8 & H-11), 7.63 (d, 2H, J_m = 1.6 Hz, H-3 & H-4), 7.67-7.65 (dd, 2H, J_o = 8.4 Hz, J_m = 1.2 Hz, H-9 & H-10), 8.23-8.21 (dd, 2H, J_o = 7.6 Hz, J_m = 1.2 Hz, H-7 & H-12), 8.24 (d, 2H, J_m = 2.0 Hz, H-1 & H-6). **GC-MS** (retention time = 13.29 min) m/z: 364.2 (M^+).

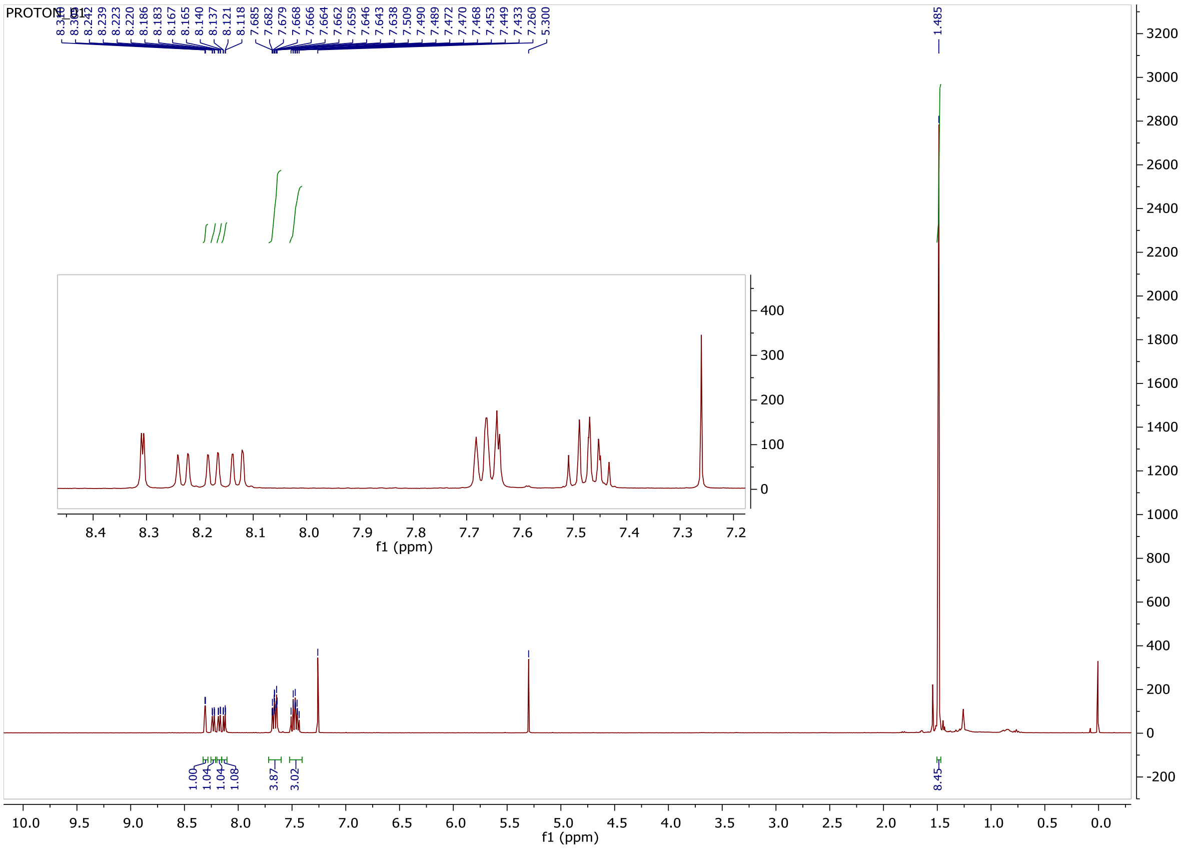

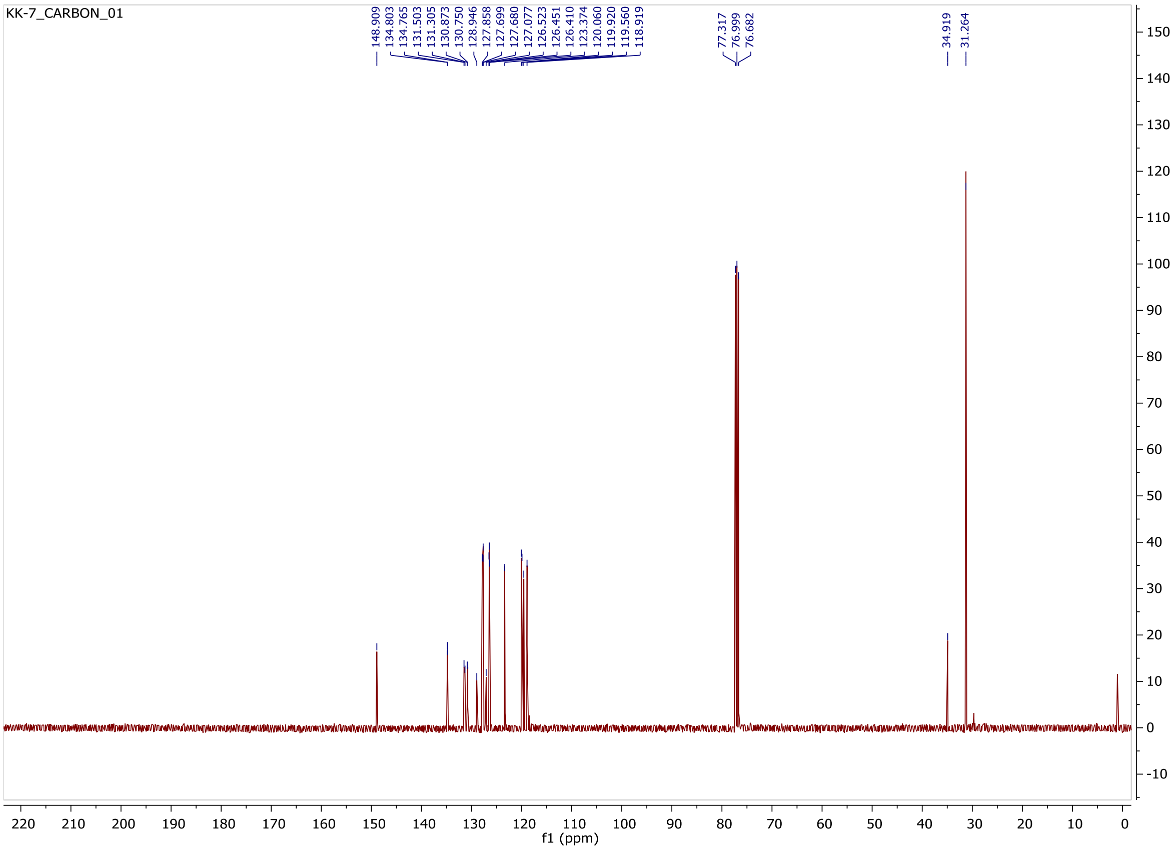

Figure S22. ^{1}H (400 MHz) and ^{13}C (100 MHz) NMR spectra of **2j** in $CDCl_3$.

5. Experiments related to mechanistic pathway

Perylene (**1a**) (500 mg, 2.0 mmol), *n*-BuLi (2.2 M in hexane, 8.0 mmol, 3.6 mL) were reacted in THF (80 mL) under nitrogen at -30 °C for 2.5 h. Aqueous work up followed by flash column chromatography on silica gel (80 g column, flow rate 35 mL/min) using Hexane:DCM (90:10) afforded 60 mg of 1-butyl-1,12b-dihydroperylene (**2a'**) in sufficient purity for characterization. Later fractions gave mixture of **2a** and **2a'**. The structure was confirmed by detailed 1D and 2D NMR and GC-MS experiments.

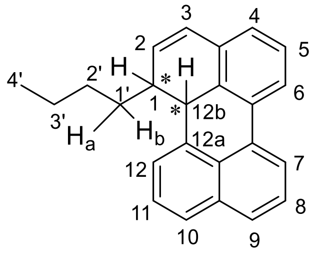

2a'

^{1}H NMR (400 MHz, CDCl$_3$): δ = 0.78 (t, 3H, J = 7.2 Hz, H-4'), 1.06-1.34 (m, 6H, H-1', H-2' & H-3'), 3.00-3.06 (m, 1H), 4.83 (d, 1H, J = 6.0 Hz, H-12b), 6.51 (dd, 1H, J_1 = 9.6 Hz, J_2 = 6.4 Hz, H-2), 6.64 (d, 1H, J_1 = 9.6 Hz, H-3), 7.10 (dd, 1H, J_1 = 7.2 Hz, J_2 = 1.2 Hz), 7.33 (ddd, 1H, J_1 = 8.0 Hz, J_2 = 7.2 Hz, J_3 = 0.8 Hz), 7.50-7.57 (m, 3H), 7.74-7.76 (m, 1H), 7.79-7.77 (m, 1H), 8.01 (m, 1H, J_1 = 8.0 Hz, J_2 = 0.8 Hz), 8.06 (m, 1H). **^{13}C NMR (100 MHz, CDCl$_3$):** δ = 14.0 (**C**-4'), 22.8, 27.9, 29.8, 38.5 (**C**-1), 41.0 (**C**-12b), 118.6, 121.7, 123.6, 125.4, 125.8, 126.0, 126.1, 126.8, 127.0, 127.8, 128.8, 130.2, 131.0, 131.49, 131.5, 134.1, 134.3, 134.7, 134.9. **GC-MS** (retention time = 11.030 min) m/z: 310 (M^+).

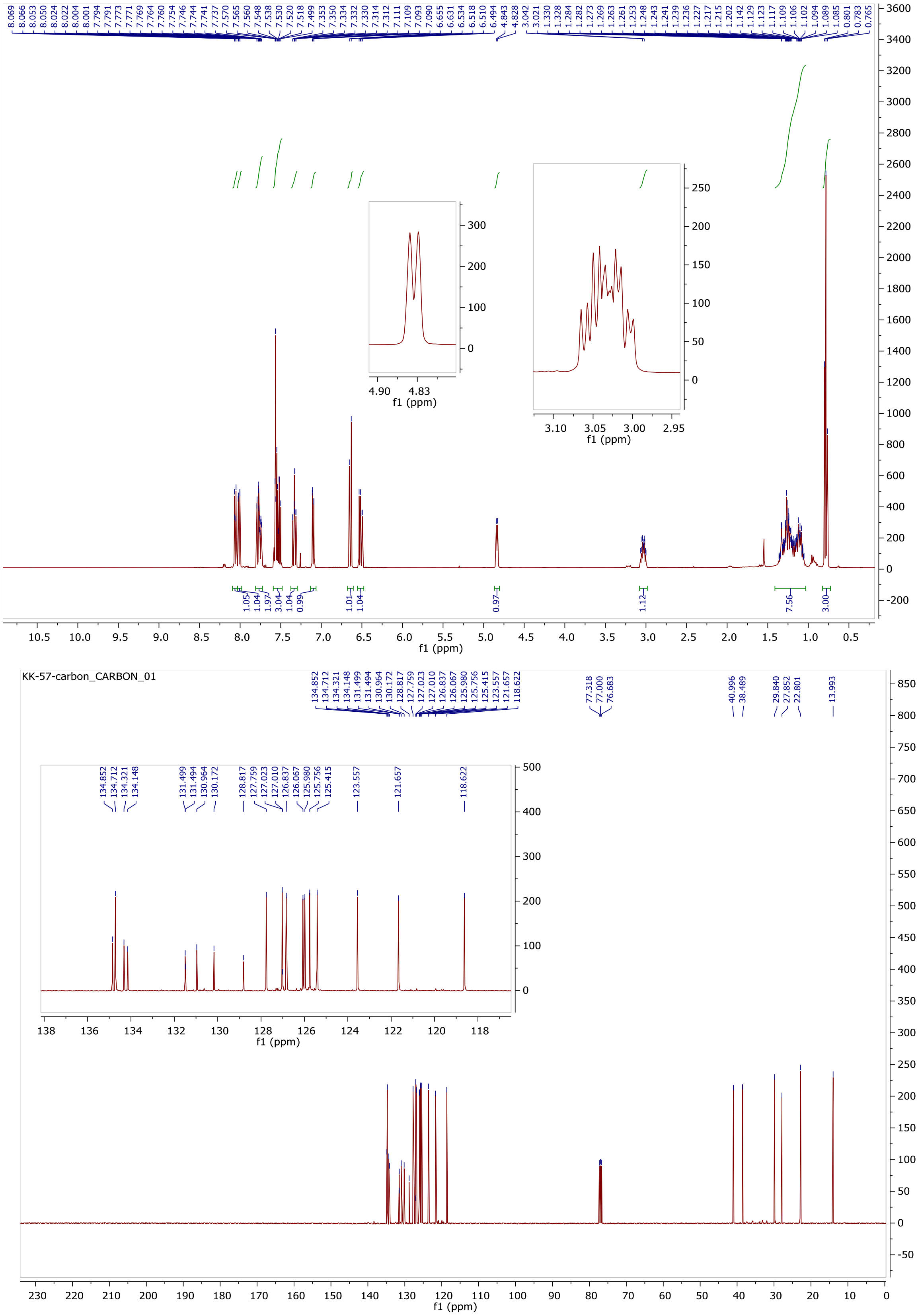

Figure S23. ^{1}H (400 MHz) and ^{13}C (100 MHz) NMR spectra of **2a'** in $CDCl_3$.

```
File        :D:\MassHunter\GCMS\All users\Data\Karl Borjesson\khushbu kus
...          hwaha\KK-57-Carbon.D
Operator    : alex
Instrument  :    5977
Acquired    : 18 Oct 2017  10:45      using AcqMethod Khushbu_method.M
Sample Name: Khushbu
Misc Info   :
```

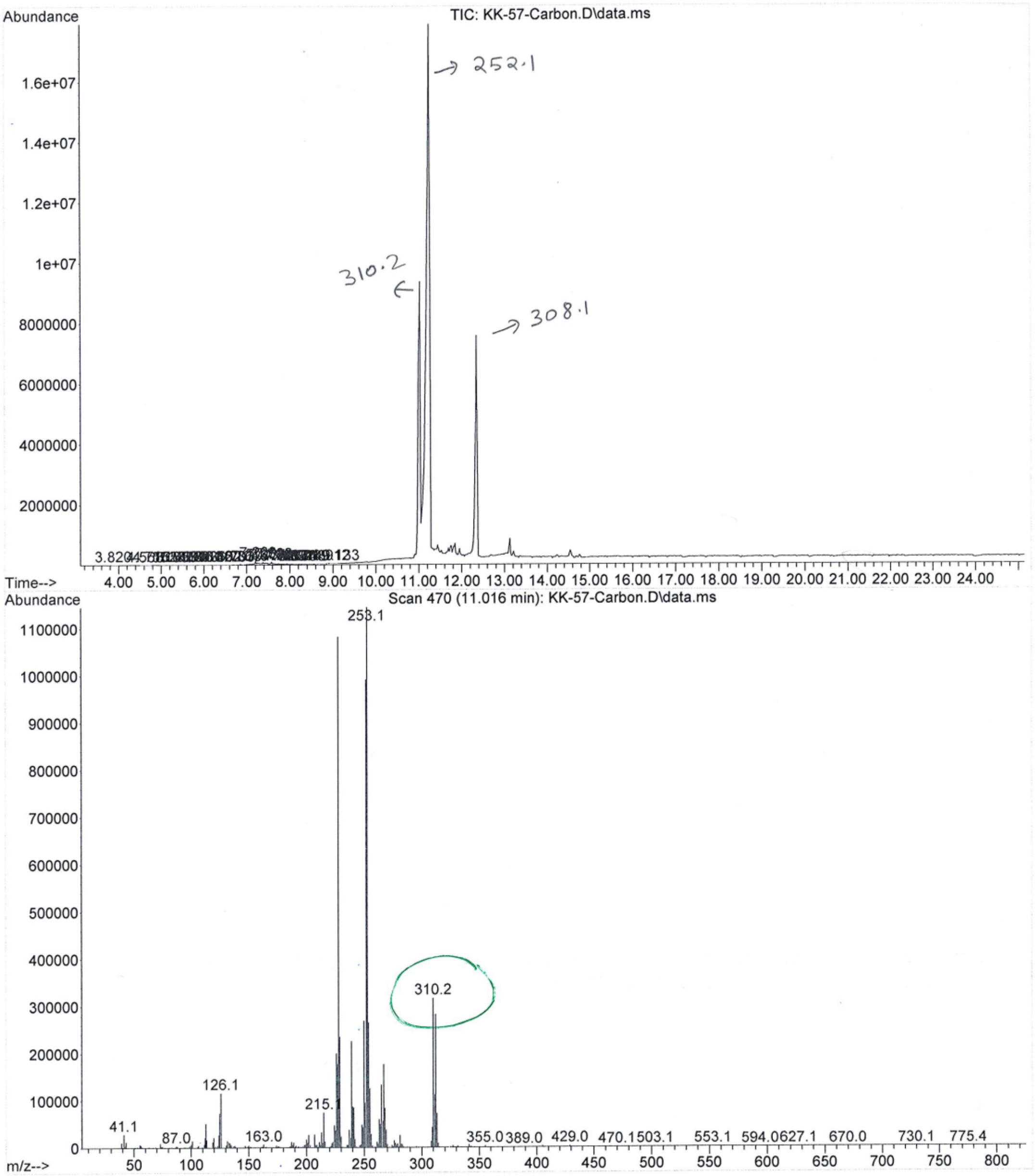

Figure S24. GC-MS spectrum of **2a'** in $CDCl_3$.

Reaction of 2a' with Oxygen

1-Butyl-1,12b-dihydroperylene (**2a'**) (25 mg) was dissolved in hexane (5 mL) and connected to O_2 balloon. After 12 h, ^{1}H NMR was checked which showed signals corresponding to **2a'** decreased and an increase in signals for **2a** is observed.

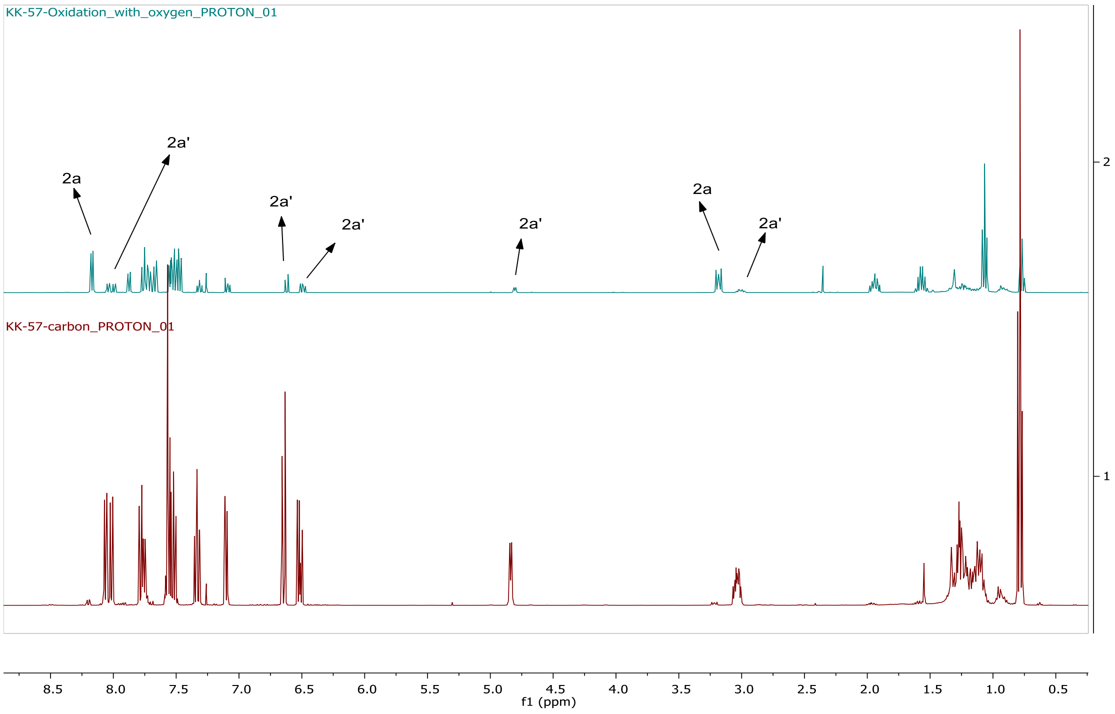

Figure S25. Overlay of spectrum before and after reaction with oxygen in $CDCl_3$.

Reaction with DDQ

1-Butyl-1,12b-dihydroperylene (**2a'**) (25 mg, 1.0 equiv.) was dissolved in Benzene (5 mL) and DDQ (22 mg, 1.2 equiv.) was added. After 5 h, crude ^{1}H NMR showed signals for **2a**.

Experiment for trapping Lithiated monoanion (1a') with MeI

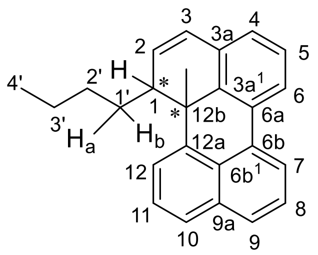

3a

Perylene (**1a**) (126 mg, 0.5 mmol), *n*-BuLi (2.2 M in hexane, 0.5 mmol, 0.19 mL) were reacted in dry THF (80 mL) under nitrogen at -30 °C for 2.5 h following the general procedure. Afterwards, excess of MeI (212 mg, 3.0 equiv.) was added dropwise. Color of the reaction

changed from greenish to red-orange after 10 minutes. Reaction was allowed to come at 0 °C in approximately 1 h. Flash column chromatography on silica gel (80 g column, 50 mL/min) cyclohexane:toluene (90:10) resulted in pure cream solid **3a**. mp (DSC): 126 °C (), yield (80 mg, 50%).

^{1}H NMR (400 MHz, $CDCl_3$): δ = 0.75 (t, 3H, J = 7.2 Hz, H-4'), 0.95-1.42 (m, 6H), 1.44 (s, 3H, C**H_3**-C-12b), 2.93 (ddd, 1H, J_1 = 11.5 Hz, J_2 = 6.4 Hz, J_3 = 3.2 Hz, H-1), 6.38 (dd, 1H, J_1 = 9.5 Hz, J_2 = 6.4 Hz, H-2), 6.59 (d, 1H, J_1 = 9.5 Hz, H-3), 7.09 (dd, 1H, J_1 = 7.3 Hz, J_2 = 1.2 Hz, H-4), 7.32 (dd, 1H, J_1 = 8.0 Hz, J_2 = 7.3 Hz, H-5), 7.58-7.51 (m, 3H, H-8, H-11, H-10), 7.75 (dd, 1H, J_1 = 7.6 Hz, J_2 = 1.6 Hz, H-12), 7.83-7.80 (m, 1H, H-9), 8.05 (dd, 1H, J_1 = 8.2 Hz, J_2 = 1.2 Hz, H-6), 8.13-8.11 (m, 1H, H-7). **^{13}C NMR (100 MHz, $CDCl_3$):** δ = 14.0 (**C-4'**), 22.8, 30.0, 31.3, 34.1(**C**H_3-C-12b), 42.2 (q, **C-12b**), 43.9 (**C-1**), 118.9 (**C-7**), 122.2 (**C-6**), 122.7 (**C-11**), 125.6 (**C-12**), 125.9, 126.3, 126.36, 126.39, 126.7 (**C-5**), 127.1 (q), 128.0 (**C-9**), 129.5 (q, **C-6b**), 131.0 (q, **C-6a**), 132.8 (**C-2**), 133.0 (q, **C-3a**), 134.0 (q), 136.3 (q, **C-3b**), 141.0 (q, **C-12a**). **IR (ATR cm^{-1}): ν** = 2924, 1580, 1454, 1375, 1263, 812, 774, 734, 558. **GC-MS** (retention time = 10.684 min) m/z: 324.2 (M^+), 309.2 (M^+ -CH_3). **HRMS (APCI):** *m*/*z* calcd for $(C_{25}H_{22}+H)^+$: 325.1951; found: 325.1964.

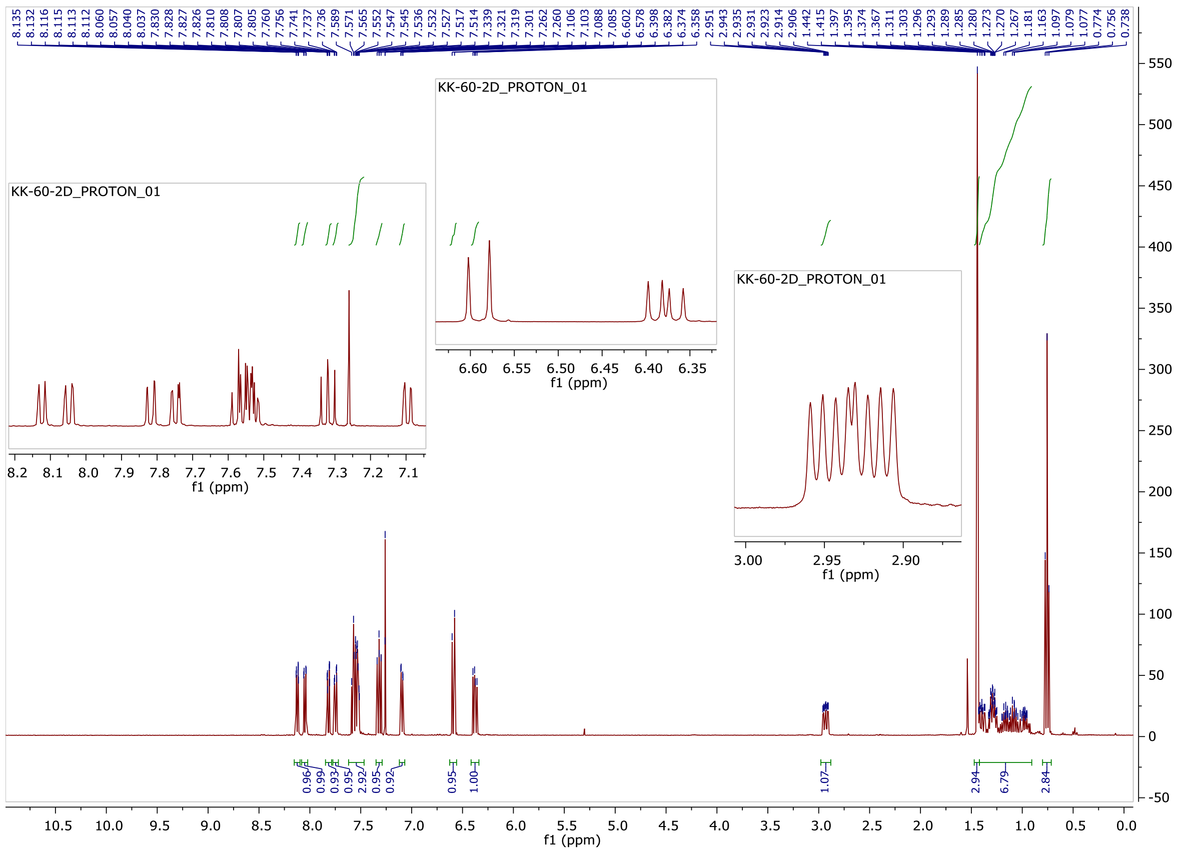

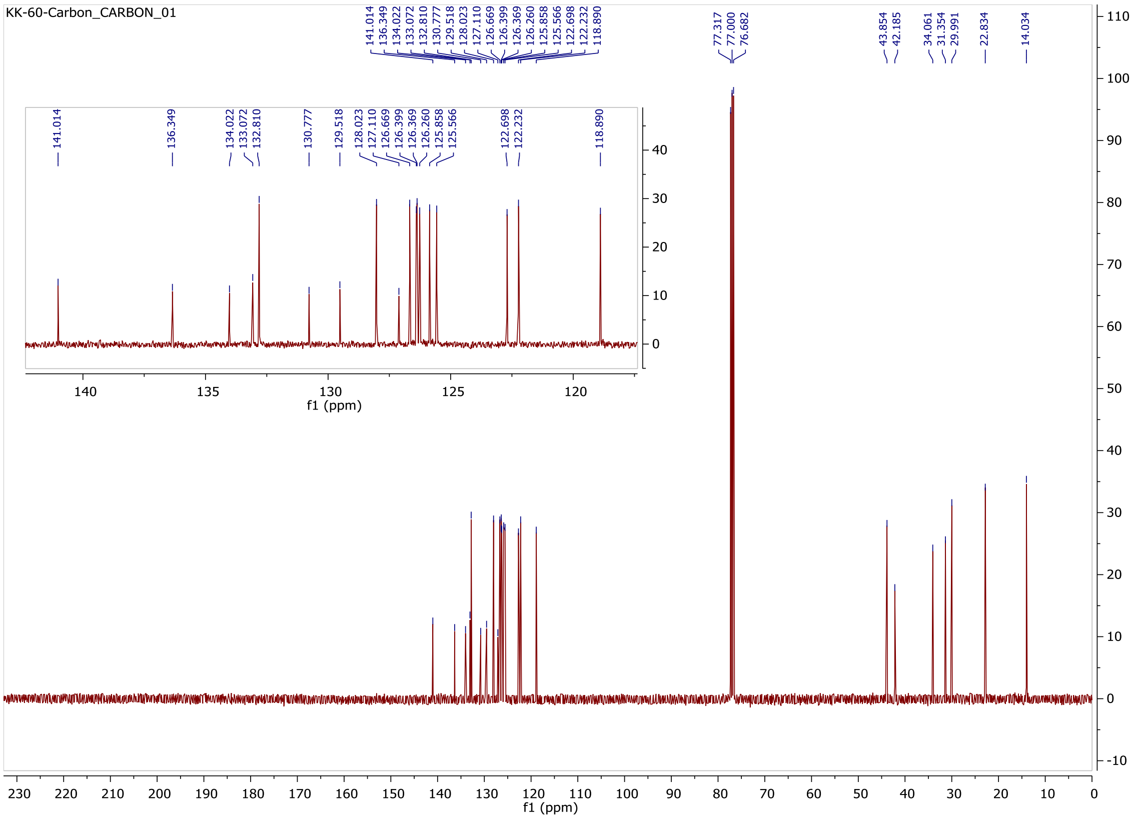

Figure S26. ^{1}H (400 MHz) and ^{13}C (100 MHz) NMR spectra of **3a** in $CDCl_3$.

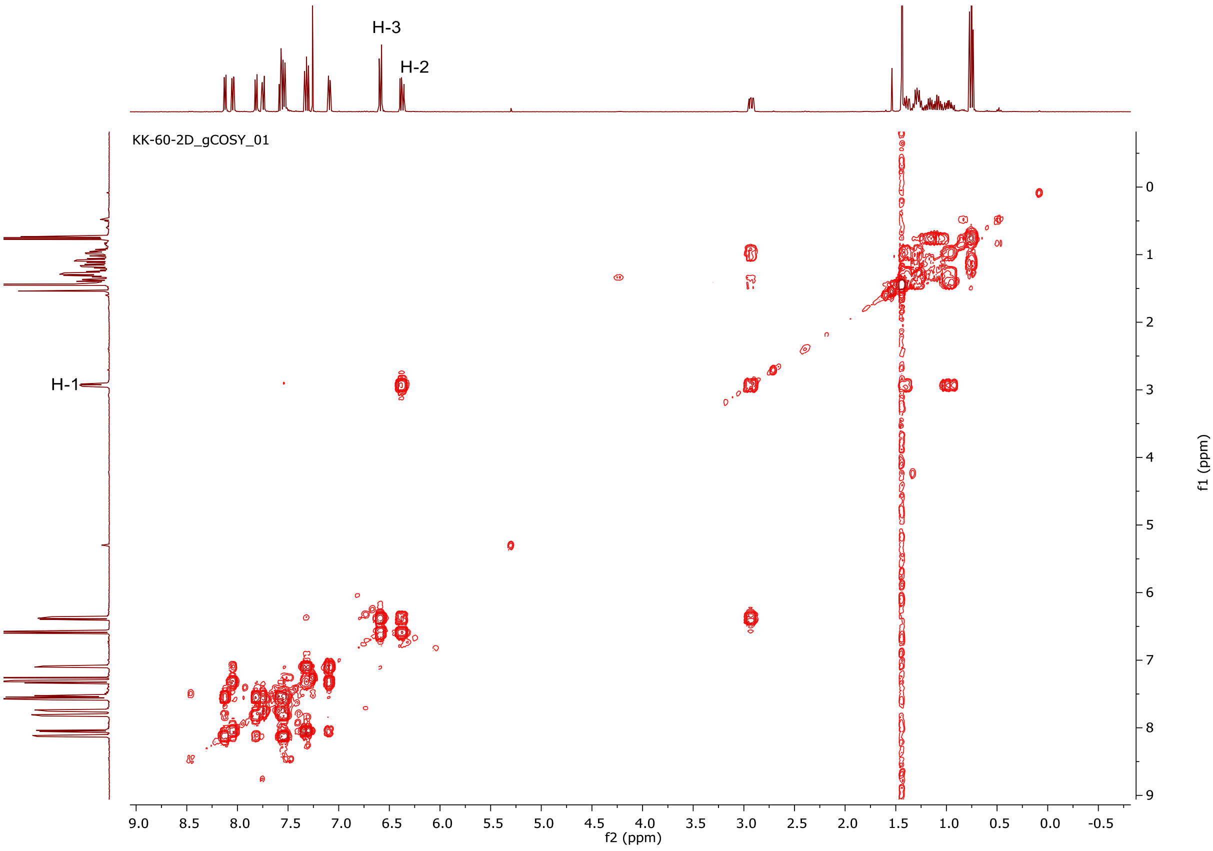

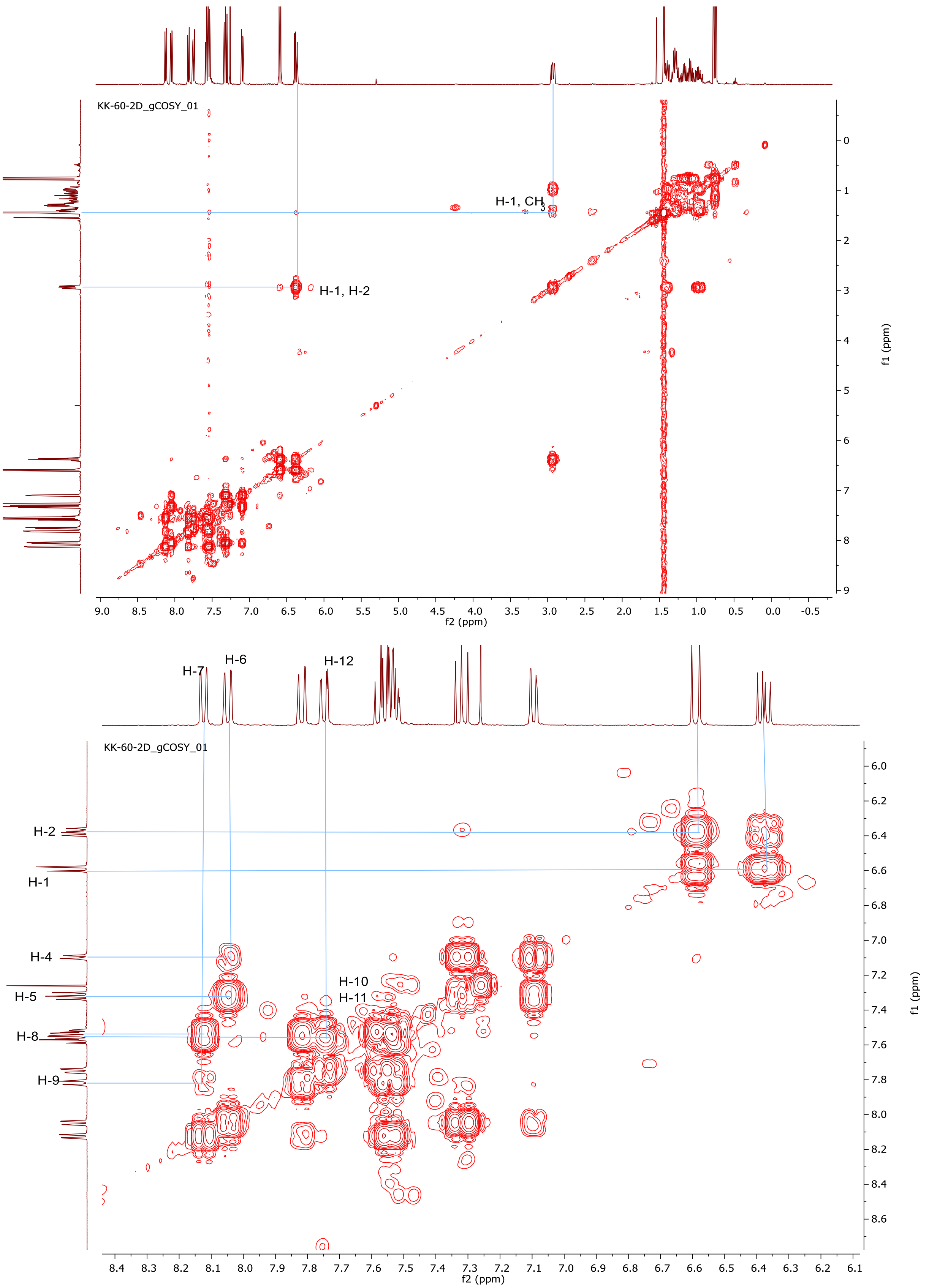

Figure S27. COSY NMR spectra of **3a** in $CDCl_3$.

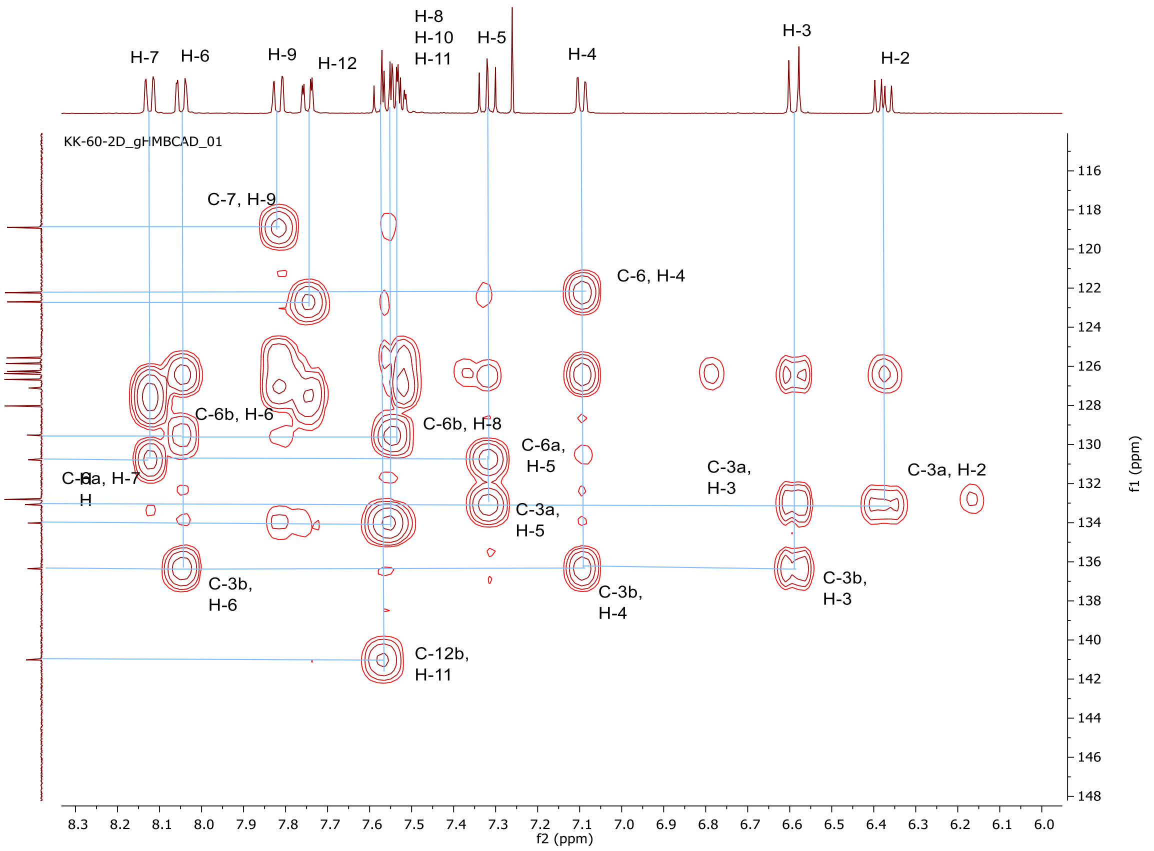
KK-60-2D_gHMBCAD_01
H-7
H-6
H-9
H-12
H-8
H-10
H-11
H-5
H-4
H-3
H-2
C-7, H-9
C-6, H-4
C-6b, H-6
C-6b, H-8
C-6a, H-5
C-3a, H-3
C-3a, H-2
C-3a, H-5
C-3b, H-6
C-3b, H-4
C-3b, H-3
C-12b, H-11
f2 (ppm)
f1 (ppm)

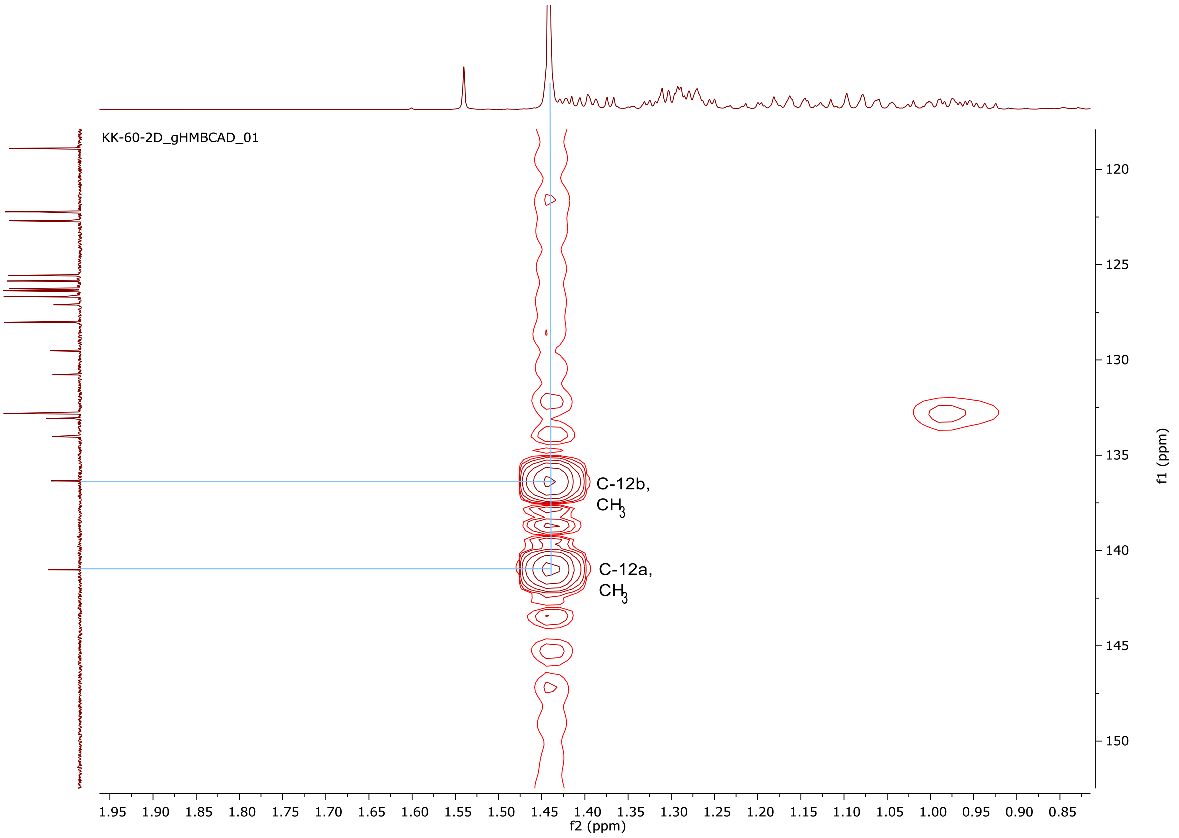
KK-60-2D_gHMBCAD_01
C-12b, CH3
C-12a, CH3
f2 (ppm)
f1 (ppm)

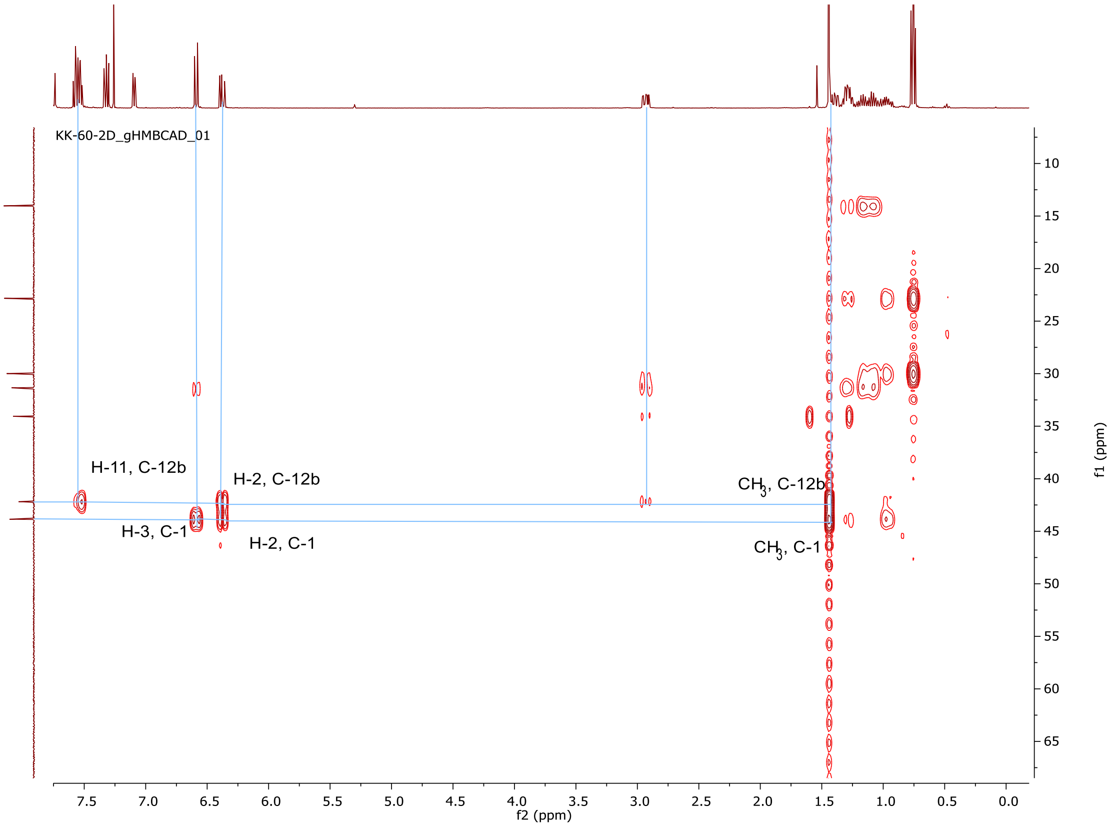

Figure S28. HMBC NMR spectra of **3a** in $CDCl_3$.

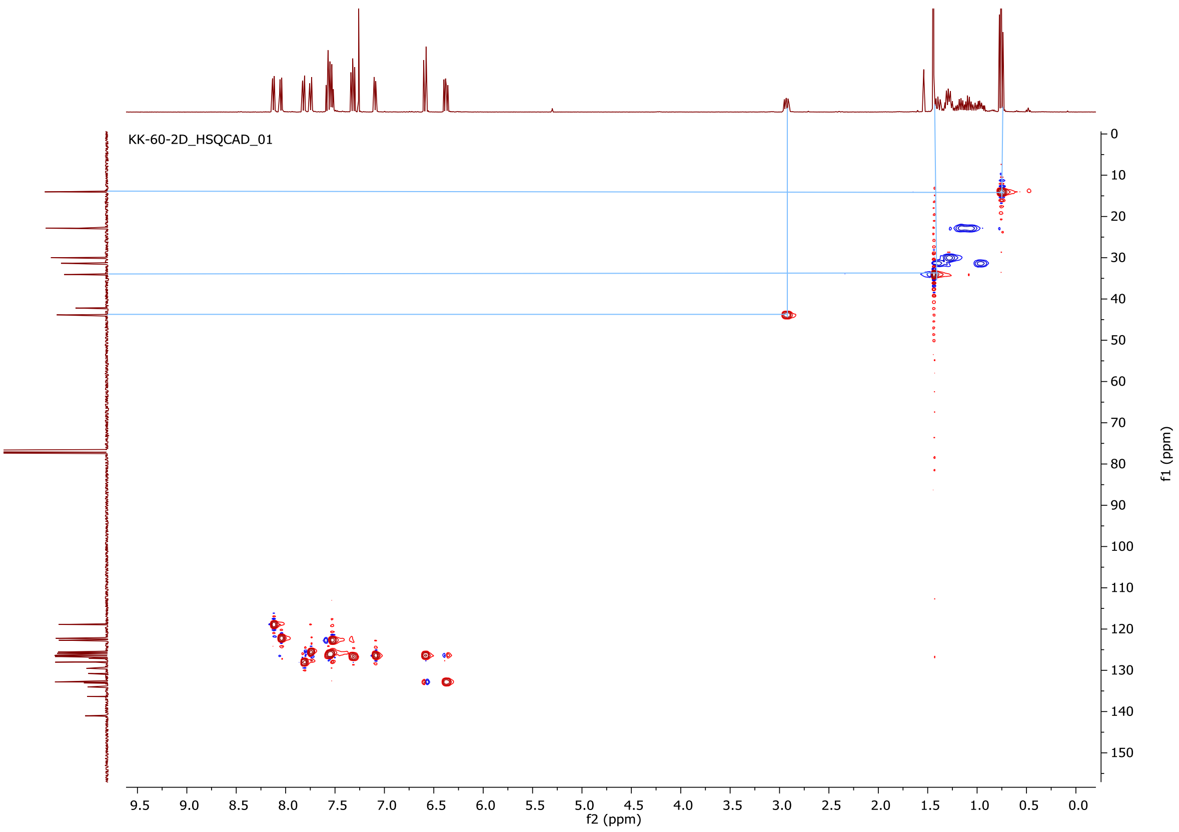
KK-60-2D_HSQCAD_01
f2 (ppm)
f1 (ppm)

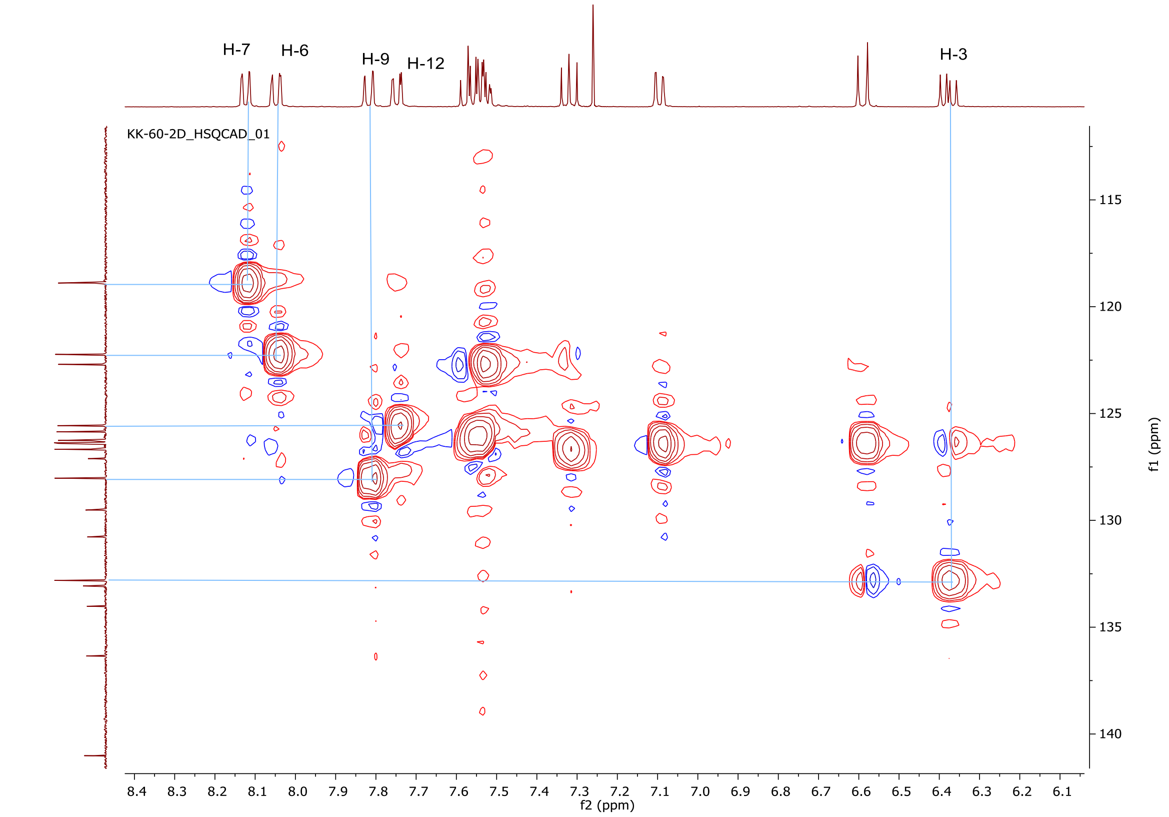
H-7
H-6
H-9
H-12
H-3
KK-60-2D_HSQCAD_01
f2 (ppm)
f1 (ppm)

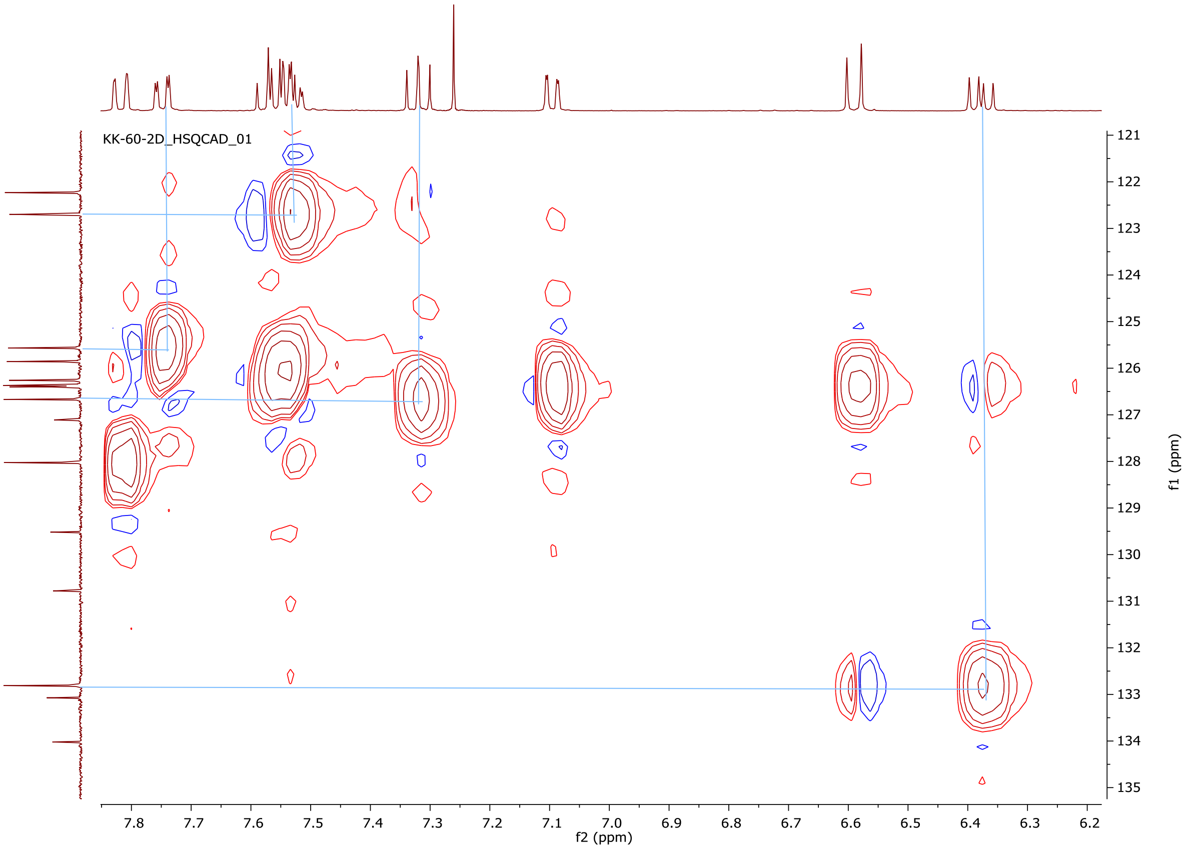

Figure S29. HMBC NMR spectra of **3a** in $CDCl_3$.

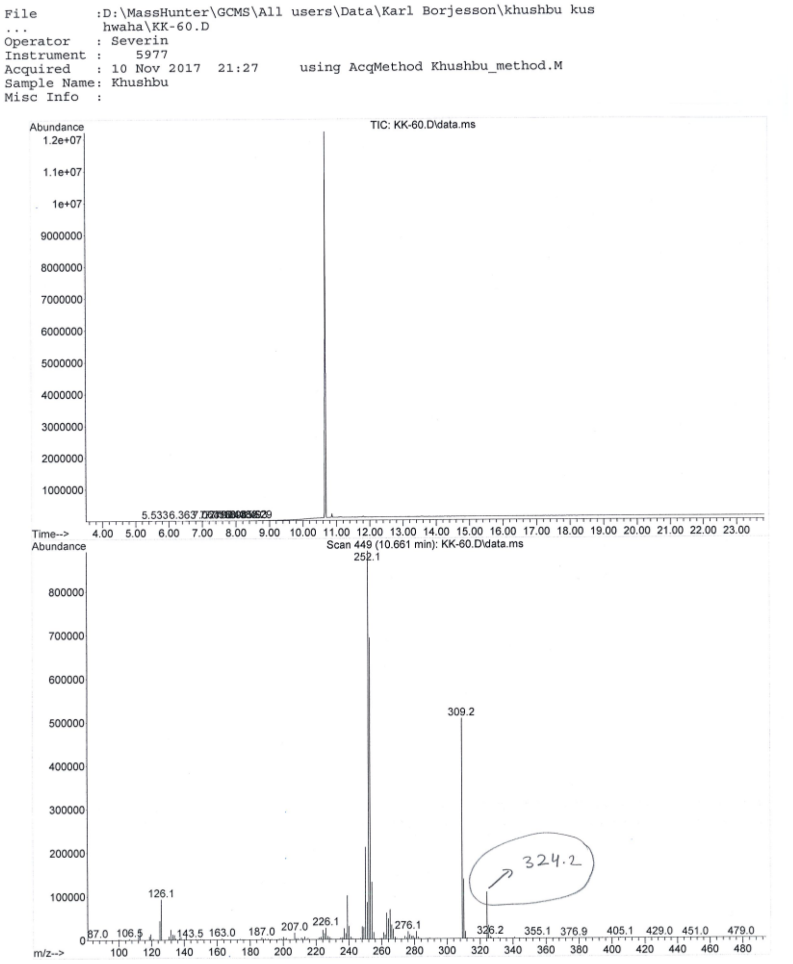

Figure S30. GC-MS spectra of **3a**.

6. Low temperature NMR for 2a

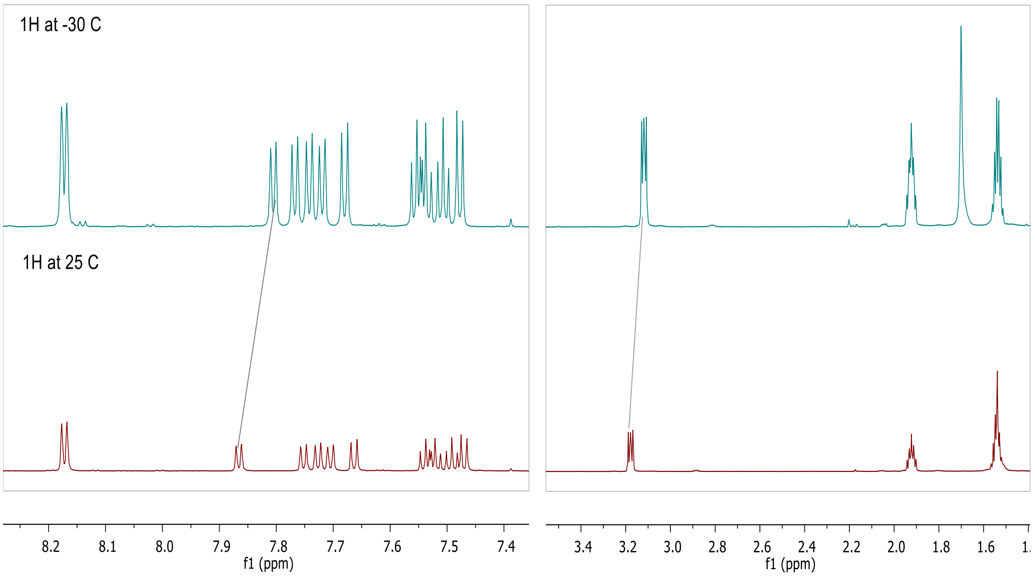

Figure S31. ^{1}H NMR of *n*-butylperylene at -30 °C (up) and 25 °C (down).

7. Single crystal X-ray of 2c

Method and data: X-Ray crystallographic data was obtained on Single Crystal X-ray diffractometer Bruker D8 VENTURE which is equipped with the PHOTON 100CMOS detector. The diffractometer is equipped with Incotec Cu K_α (λ = 1.54184 Å) or Mo K_α (λ = 0.71073 Å) microfocus X-ray sources, and a Cryojet low-temperature device. The cryojet was used to lower the temperature to 100K for data collection. A small crystal of about 0,1 * 0.05 * 0.03 mm in size was used for collecting the single crystal data. The crystal was gently mounted on a string loop and studied at cryo conditions. A full sphere of data to 0.65 Å resolution was collected with the Mo radiation source. In total about 20h of data collection, no change or physical deformation was observed in the mounted crystal.

The data were processed using APEX3 software. Unit cell dimensions were refined using the entire data sets. The structure was solved (direct methods) and refined (full-matrix least-squares on *F*2) using SHELXL. Non-hydrogen atoms were refined anisotropically, while hydrogen atoms were introduced at calculated positions as riding on their corresponding carbon atoms and refined isotropically. Geometric calculations were carried out using the Shelx, PLATON and Olex 2[4] software packages.

Phase data

**Formula sum**	C96 H80
**Formula weight**	1233.69.g/mol
**Crystal system**	monoclinic
**Space-group**	P 1 21/n 1 (14)
**Cell parameters**	a=9.1323(5).Å b=11.9121(7) Å c=15.1512(9) Å β=105.422(1)°
**Cell ratio**	a/b=0.7666 b/c=0.7862 c/a=1.6591
**Cell volume**	1588.87(16) Å^3
**Calc. density**	1.28926.g/cm^3
**Wyckoff sequence**	e44

Atom	Wyck.	Site	x/a	y/b	z/c	U [Å^2]
C1	4e	1	0.61865	0.60368	0.27461	
C2	4e	1	0.49360	0.53290	0.23089	
C3	4e	1	0.44258	0.53780	0.13626	

H3	4e	1	0.36810	0.48544	0.10524	0.020
C4	4e	1	0.49744	0.61769	0.08472	
H4	4e	1	0.45876	0.61898	0.02000	0.024
C5	4e	1	0.60569	0.69342	0.12668	
H5	4e	1	0.63689	0.75054	0.09185	0.024
C6	4e	1	0.67128	0.68659	0.22222	
C7	4e	1	0.79337	0.75716	0.26593	
H7	4e	1	0.82769	0.81326	0.23157	0.025
C8	4e	1	0.86238	0.74539	0.35706	
H8	4e	1	0.94276	0.79452	0.38603	0.028
C9	4e	1	0.81547	0.66124	0.40833	
H9	4e	1	0.86514	0.65413	0.47167	0.024
C10	4e	1	0.69802	0.58811	0.36853	
C11	4e	1	0.52532	0.42762	0.37646	
C12	4e	1	0.65955	0.49046	0.41717	
C13	4e	1	0.75024	0.45786	0.50211	
H13	4e	1	0.84228	0.49711	0.52776	0.024
C14	4e	1	0.70976	0.36873	0.55096	
H14	4e	1	0.77390	0.34823	0.60901	0.026
C15	4e	1	0.57770	0.31113	0.51504	
H15	4e	1	0.54901	0.25212	0.54919	0.024
C16	4e	1	0.48387	0.33864	0.42777	
C17	4e	1	0.34712	0.27993	0.38966	
H17	4e	1	0.32097	0.21731	0.42119	0.023
C18	4e	1	0.25267	0.31239	0.30819	
H18	4e	1	0.15951	0.27342	0.28533	0.022
C19	4e	1	0.28833	0.40233	0.25618	
C20	4e	1	0.43103	0.45380	0.28675	
C21	4e	1	0.16561	0.44119	0.17301	
H21A	4e	1	0.17719	0.39893	0.11901	0.019
H21B	4e	1	0.18325	0.52146	0.16237	0.019
C22	4e	1	0.00073	0.42776	0.17854	
H22	4e	1	-0.02187	0.34588	0.18119	0.021

C23	4e	1	-0.10706	0.47747	0.09259	
H23A	4e	1	-0.21230	0.46664	0.09500	0.032
H23B	4e	1	-0.09150	0.43975	0.03831	0.032
H23C	4e	1	-0.08651	0.55789	0.08933	0.032
C24	4e	1	-0.02488	0.48441	0.26320	
H24A	4e	1	0.00331	0.56382	0.26353	0.037
H24B	4e	1	0.03779	0.44769	0.31823	0.037
H24C	4e	1	-0.13225	0.47822	0.26236	0.037

Anisotropic displacement parameters, in Å^2

Atom	U_{11}	U_{22}	U_{33}	U_{12}	U_{13}	U_{23}
C1	0.01366	0.01311	0.01806	0.00252	0.00641	-0.00184
C2	0.01252	0.01238	0.01713	0.00362	0.00446	-0.00026
C3	0.01408	0.01750	0.01720	0.00149	0.00323	-0.00037
C4	0.01945	0.02417	0.01548	0.00413	0.00475	0.00390
C5	0.02103	0.01909	0.02322	0.00212	0.01035	0.00520
C6	0.01602	0.01591	0.02238	0.00202	0.00884	-0.00041
C7	0.02134	0.01670	0.02835	-0.00287	0.01250	-0.00186
C8	0.02016	0.02156	0.02828	-0.00758	0.01079	-0.00946
C9	0.01889	0.02358	0.01785	-0.00248	0.00547	-0.00643
C10	0.01376	0.01661	0.01704	0.00147	0.00662	-0.00413
C11	0.01453	0.01323	0.01510	0.00320	0.00566	-0.00155
C12	0.01562	0.01683	0.01447	0.00271	0.00518	-0.00339
C13	0.01801	0.02304	0.01758	0.00167	0.00251	-0.00413
C14	0.02400	0.02475	0.01446	0.00694	0.00207	0.00144
C15	0.02473	0.01890	0.01826	0.00530	0.00741	0.00372
C16	0.01834	0.01528	0.01846	0.00430	0.00714	0.00093
C17	0.02162	0.01463	0.02350	0.00068	0.00900	0.00439
C18	0.01543	0.01607	0.02323	-0.00166	0.00474	-0.00016
C19	0.01521	0.01323	0.01615	0.00217	0.00492	-0.00168
C20	0.01340	0.01201	0.01524	0.00233	0.00382	-0.00193
C21	0.01437	0.01544	0.01600	-0.00082	0.00238	-0.00130

C22	0.01427	0.01843	0.01779	-0.00030	0.00237	-0.00222
C23	0.01575	0.02393	0.02213	0.00027	0.00098	-0.00080
C24	0.02020	0.03252	0.02146	0.00500	0.00437	-0.00657

8. DSC of 2a:2d and 2b:2d mixtures

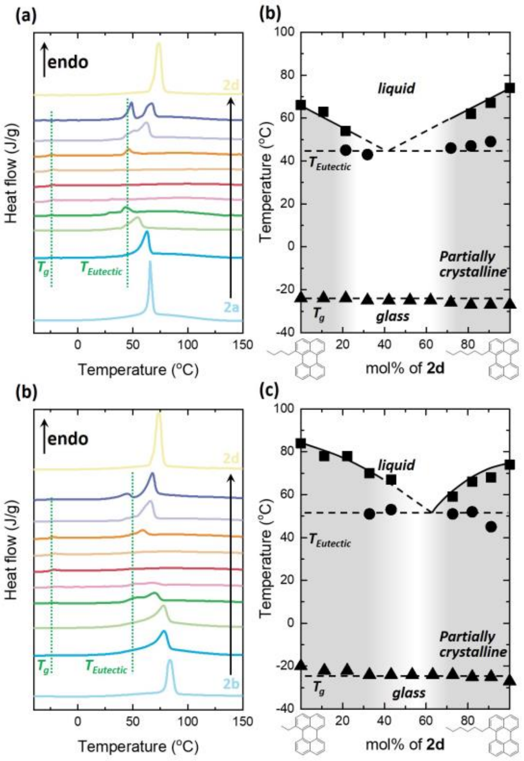

Figure S32. DSC thermogram representing the initial heating half-cycle for blends of **2a**:**2d** (a) and **2b**:**2d** (c). Glass transition (T_g) and eutectic transition ($T_{eutectic}$) temperatures are marked by vertical dotted lines. Schematic showing a phase diagram for blends of **2a**:**2d** (b) and **2b**:**2d** (d). The liquidus (solid lines) are sketched following the melting temperature (squares) of each blend. The glass transition and eutectic transition (dashed lines) are sketched following the enthalpy relaxation peak of glass transition (triangles) and the peak temperature for the eutectic point (circles), respectively. The lightly dotted area indicates the region where the blend remains liquid.

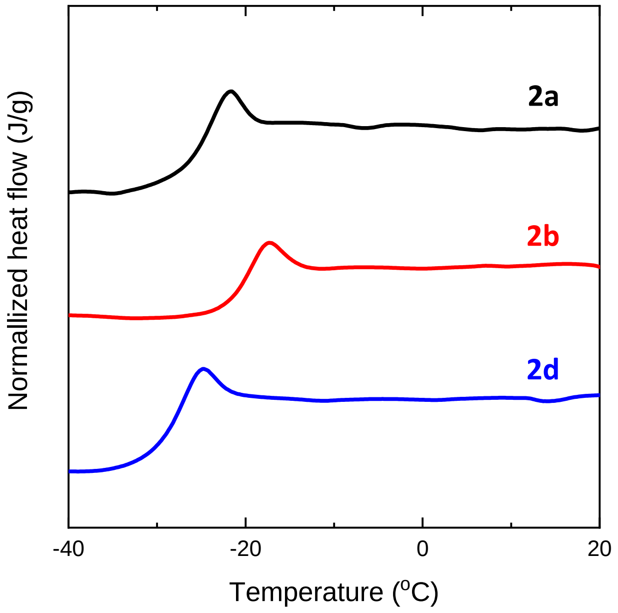

Figure S33. DSC thermogram representing the second heating cycle of the neat materials (**2a**, **2b** and **2d**) collected from solution cast from DCM after kept at -50 °C for 30 min, highlighting the T_g of the three neat materials. The heat flow is normalized to the total sample weight.

Table S3: Measured melting points using DSC.

Entry	onset	peak	offset
**1**	276	278	281
**2a**	63	70	74
**2b**	78	82	86
**2c**	114	117	120
**2d**	66	71	75
**2e**	50	56	61
**2f**	116	121	126
**2g**	127	131	134
**2h**	217	222	225
**2i**	321	341	360
**2j**	130	146	150

9. References

1. H. E. Zieger, *J. Org. Chem.* 1966, **31**, 2977.
2. M. Strauch, PhD Thesis, Göttingen, Georg-August-Universitat (Germany) Synthesen und photophysikalische Untersuchungen von Derivaten des Perylens, 1990.
3. C. Zimmermann, F. Willig, S. Ramakrishna, B. Burfeindt, B. Pettinger, R. Eichberger, W. Storck, *J. Phys. Chem. B* 2001, **105**, 9245.
4. O. V. Dolomanov, R. J. Bourhis, J. A. K. Gildea, H. Puschmann, *J. Appl. Cryst.* 2009, **42**, 339.
